# New ionizable lipids for non-viral mRNA delivery with secondary amine cyclic ether head groups†

**DOI:** 10.1039/d5md00115c

**Published:** 2025-05-27

**Authors:** Eric L. Dane, Aditya R. Pote, Martin Hemmerling, Werngard Czechtizky, Liping Zhou, Annette Bak

**Affiliations:** a Advanced Drug Delivery, Pharmaceutical Sciences, R&D, AstraZeneca Boston MA USA eric.dane@astrazeneca.com; b Medicinal Chemistry, Research and Early Development, Respiratory and Immunology, BioPharmaceuticals R&D, AstraZeneca Gothenburg Sweden

## Abstract

Lipid nanoparticles (LNPs) are the most widely used non-viral delivery approach for messenger ribonucleic acid (mRNA). Among the different components in an LNP, the ionizable lipid plays critical roles in interacting with the mRNA cargo and facilitating delivery to the cytosol, as well as influencing the LNP's tissue tropism *via* the protein corona. To date the most successful ionizable lipids have relied on a tertiary amine head group as the site of protonation. We hypothesized that potent ionizable lipids based on a secondary amine could be discovered using a design, make, test and analyze (DMTA) cycle approach. Starting from a lead lipid with a secondary amine cyclic ether head group, we optimized delivery efficiency by systematically modifying the lipid linker length, tail symmetry, tail branching pattern, and head group structure. The mRNA-LNPs formulated with these lipids were evaluated *in vivo* by quantifying liver protein expression. Using this rational lipid design strategy, we identified many candidates that outperformed the benchmark lipid (MC3), supporting the further development of this ionizable lipid class. Notably, several structure activity relationships (SARs) that highlight how sensitive ionizable lipid activity is to relatively minor structural changes are reported.

## Abstract 2

From a library of ionizable lipids based on a secondary amine, we identify high performers and report key structure–activity relationships driving lipid nanoparticle-based mRNA delivery.

## Graphical abstract

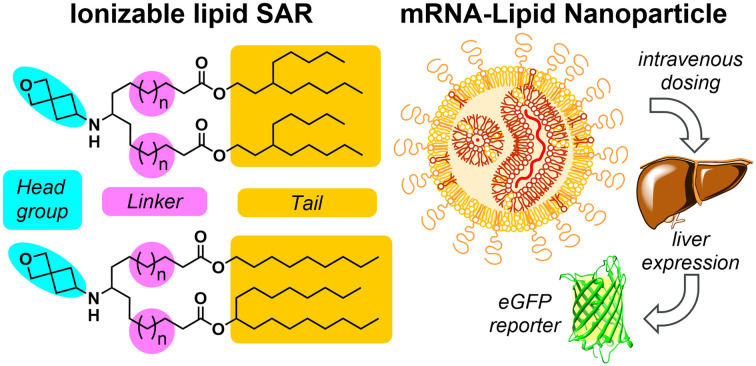

## Introduction

Messenger RNA (mRNA) as a therapeutic modality^1^ has great potential, both to prevent diseases when used as a vaccine^2^ and to treat illnesses in areas such as immune-oncology,^3^ protein replacement,^4,5^*in vivo* CAR T cell therapy,^6^ and genomic medicine.^7^ However, full realization of this potential hinges on the development of safe and effective approaches to deliver mRNA to the cytosol of the desired cells for translation into functional protein.^8^ Lipid nanoparticles (LNPs) are the most clinically advanced non-viral delivery approach for mRNA therapies and have many advantages, including being synthetic in origin, the potential for being re-dosable, and demonstrated clinical success in COVID-19 vaccines and human gene editing trials.^9,10^ A typical LNP has four components: an ionizable lipid, cholesterol, a helper lipid and a polyethylene glycol (PEG) lipid. The ionizable lipid, which complexes mRNA during formulation and then promotes endosomal escape upon cellular uptake, is an essential component that influences efficacy and toxicity.^10^ Significant efforts have been dedicated to the design of novel ionizable lipids, as documented in many publications and patents.^11^ The choice of ionizable lipid has also been shown to influence mRNA delivery in unexpected and exciting ways, such as leading to novel tissue tropism^12^ or improving vaccine immunogenicity.^13^

Notable ionizable lipids that have been used successfully in the clinic and in commercially available drug products include DLin-MC3-DMA (MC3),^14^ ALC-0315,^15^ and SM-102,^16^ with the first being used in Onpattro® (patisiran)^17^ to deliver siRNA to the liver by intravenous (i.v.) dosing and the latter two examples being used for intramuscular (i.m.) COVID-19 vaccinations, respectively in Comirnaty® (BNT162b2)^18^ and SpikeVax® (mRNA-1273).^19^ Additionally, i.v.-administered LNPs using the ionizable lipid LP01 have shown recent success in late-stage human trials delivering gene editing machinery to hepatocytes for treatment of transthyretin amyloidosis (TTR).^20^ Many current efforts in the field are focused on developing ionizable lipids to see if performance and tolerability can be further improved, especially when targeting delivery to tissues outside the liver or muscle.

**Fig. 1:**
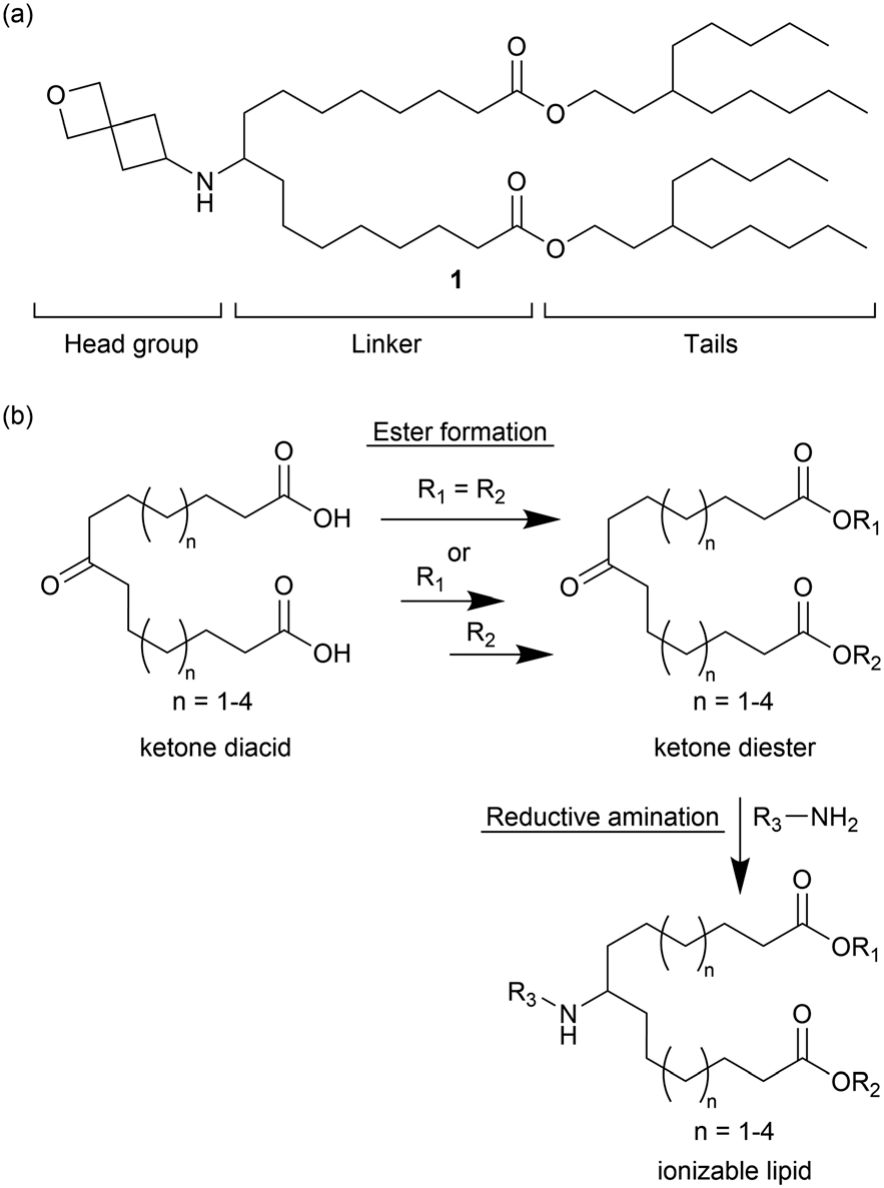
(a) Example of a secondary amine ionizable lipid with the substructures of the lipid labeled. (b) General synthetic approach for preparing lipids.

A wide breadth of ionizable lipid structures has been explored and therefore generalizations are challenging; however, it can be useful to view the lipid as having three parts: head, linker, and tail (Fig. 1a).^21^ Generally, the head group contains one or more ionizable amines that electrostatically complex the mRNA cargo when protonated. It has been shown that within an LNP the extent of amine protonation of the ionizable lipid as a function of pH influences the efficiency of endosomal escape and delivery to the cytosol.^14^ In contrast to the more polar head group, the linker and tail regions are generally hydrophobic, and their structure plays a key role in how lipids pack within LNPs. The addition of functional groups, such as esters, disulfides or silicon ethers, that can act as sites of lipid biodegradability has been a successful strategy to improve the tolerability of newer generations of ionizable lipids.^22–25^ As with amine ionization, lipid packing within LNPs has been shown to influence delivery efficiency.^26^ The addition or removal of substructures such as *cis*-double bonds or hydrocarbon branching has been used to modulate the assembly of lipids within LNPs.^27,28^

Our understanding of how ionizable lipid structure influences LNP-mediated mRNA delivery is growing but still incomplete. The exploration of new classes of lipids that differ in significant ways from the most studied examples offers the potential for improved performance and also provides opportunities for better mechanistic understanding of the delivery process. To date, the most successful lipids, including those we have highlighted, rely on a tertiary amine as the site of protonation. As part of our efforts to discover new ionizable lipids based on previously unexplored structures with the potential for improved or unique performance, we hypothesized that lipids based on a secondary amine could be optimized to match or outperform the delivery efficiency of the well-characterized benchmark tertiary amine lipid, MC3. Lipids based on secondary amines attracted our attention for several reasons. First, there is a greater opportunity to develop new intellectual property because they have been less explored. Second, in contrast to a tertiary amine, a secondary amine can act as a hydrogen bond donor in the neutral state due to the presence of the N–H bond. Headgroups capable of hydrogen bond donation have been posited to increase LNP performance and stability.^29^ Finally, in aqueous solutions secondary amines are generally stronger bases than tertiary amines with an inherently different geometry around the nitrogen,^30^ and thus we reasoned that the SAR of the substituents on the amine, for example relating to the optimal size and the electron-withdrawing *versus* electron-donating character, may be significantly different relative to the SAR previously observed for tertiary amine lipids. Herein, we report our synthesis and evaluation of a library of secondary amine lipids for mRNA-loaded LNPs. Through systematic variations of key structural elements including the linker, tail, and head group, we elucidated clear structure activity relationships that can guide future design of high-performing ionizable lipids.

## Results and discussion

As part of an effort to identify active lipids containing an ionizable secondary amine, preliminary screening (data not shown) indicated that lipid 1 (Fig. 1a) could promote effective encapsulation of mRNA in LNPs and subsequent intracellular delivery. Notably, lipid 1 contained a spirocyclic oxetane head group derived from 2-oxaspiro[3.3]heptan-6-amine. Oxetanes have been extensively explored for use in drug discovery because of their ability to replace *gem*-dimethyl and carbonyl groups with metabolically more stable and polar, solubility-enhancing functionalities, but are not common in ionizable lipids.^31^ We reasoned that the compact and conformationally constrained spirocyclic oxetane substructure had the correct polarity, size, and electron-withdrawing properties to promote the needed amine ionization and lipid packing behavior for efficient nucleic acid delivery.

To understand the influence of lipid structure on excipient performance, we undertook a study of how linker carbon chain length, tail architecture, and head group structure affected *in vivo* mRNA delivery using a design, make, test and analyse (DMTA) cycle.^32^ The design strategy focused on systematically varying structural elements within the lipid by making relatively minor changes guided by results from previous DMTA cycles. The general synthetic approach for making these lipids is shown in Fig. 1b. Using a series of ketone diacids that vary in linker length from *n* = 1–4, we prepared lipids with symmetric tails (R_1_ = R_2_) using a single diesterification reaction and lipids with asymmetric tails (R_1_ ≠ R_2_) using sequential mono esterification reactions. Notably, preparation of the ketone diacids was easily scalable as they could be purified by recrystallization. To prepare diesters for asymmetric tailed lipids, the ketone diacid (1 equiv.) was reacted with heptadecan-9-ol (0.5–0.6 equiv.) as the limiting reagent to promote formation of the mono esterification product. Both the desired mono- and undesired diesterification products were observed in the crude reaction mixture, but could be readily separated using flash silica chromatography. Yields for monoesterification products varied from 28 to 44% based on the alcohol. Conversion of the second carboxylic acid into an ester proceeded as expected and gave yields ranging from 40–84%. Subsequently, the head group was installed *via* a reductive amination of a primary amine and the final lipid was purified to greater than 95% purity based on UPLC-CAD analysis.

We first tested whether candidate lipids led to high quality LNPs under a set of standardized formulation conditions using a modified mRNA cargo encoding an eGFP reporter protein. The lipid nanoparticle composition, N/P ratio, and formulation process were held constant in order to focus specifically on the effect of the ionizable lipid. The LNP composition was ionizable lipid/cholesterol/DSPC/DMPE-PEG2000 (50/38.5/10/1.5 mol%) and the ratio between the nitrogen atoms on the ionizable lipid and phosphorus atoms of the mRNA (N/P ratio) was 3 : 1. Lipids that formed LNPs with a high (>85%) encapsulation efficiency (% EE) of mRNA and a *Z*-average diameter of less than 100 nm as measured by dynamic light scattering (DLS) (see Table S1†) were prioritized for further evaluation *in vivo* in mice. The LNPs tested were stable in PBS buffer for a minimum of two weeks and did not show significant changes in size or encapsulation over this period (data not shown). Specifically, LNPs were administered intravenously (0.3 mg kg^−1^ RNA dose) *via* tail vein injection and the amount of eGFP expressed in the liver after 24 hours was quantified by ELISA. An MC3 LNP with the same cargo, based on the FDA-approved formulation used in the siRNA therapy Onpattro®,^33^ was included in each study as a benchmark for comparison.

**Fig. 2:**
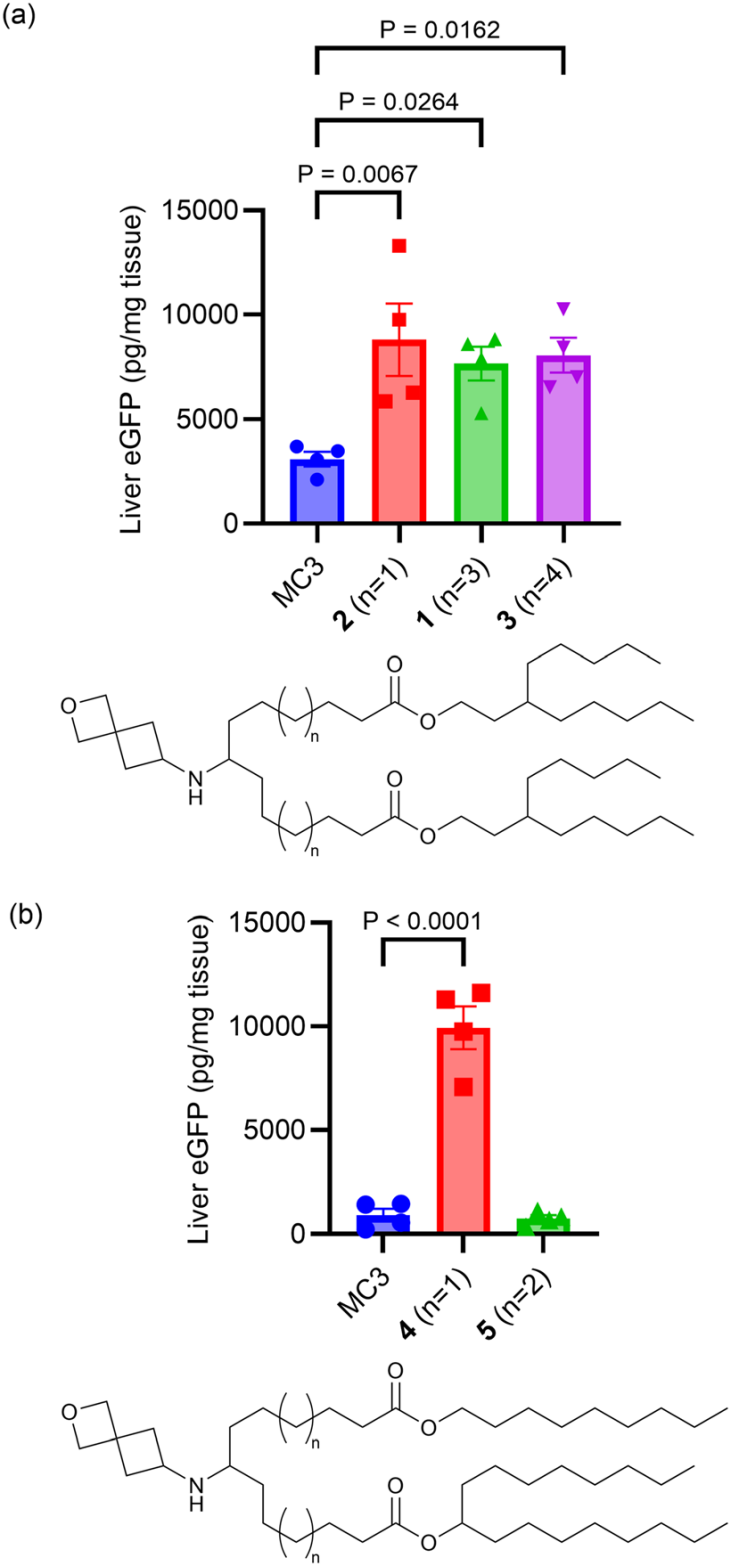
The effects of linker length on protein expression in liver following systemic administration of eGFP mRNA-LNPs containing lipids with symmetrical tails (a) and asymmetrical tails (b) in mice are shown. For (a and b), mice (*N* = 4, BALB/c mice per group) were injected i.v. with 0.3 mg kg^−1^ eGFP mRNA LNPs with terminal collection of liver samples at 24 h post-dose. eGFP levels were quantified by ELISA. Error bars are the standard error of the mean (S.E.M.) and statistical comparisons were based on a one-way ANOVA with Dunnett's test for multiple testing correction comparing each lipid with MC3 evaluated concurrently in the same experiment.

**Fig. 3:**
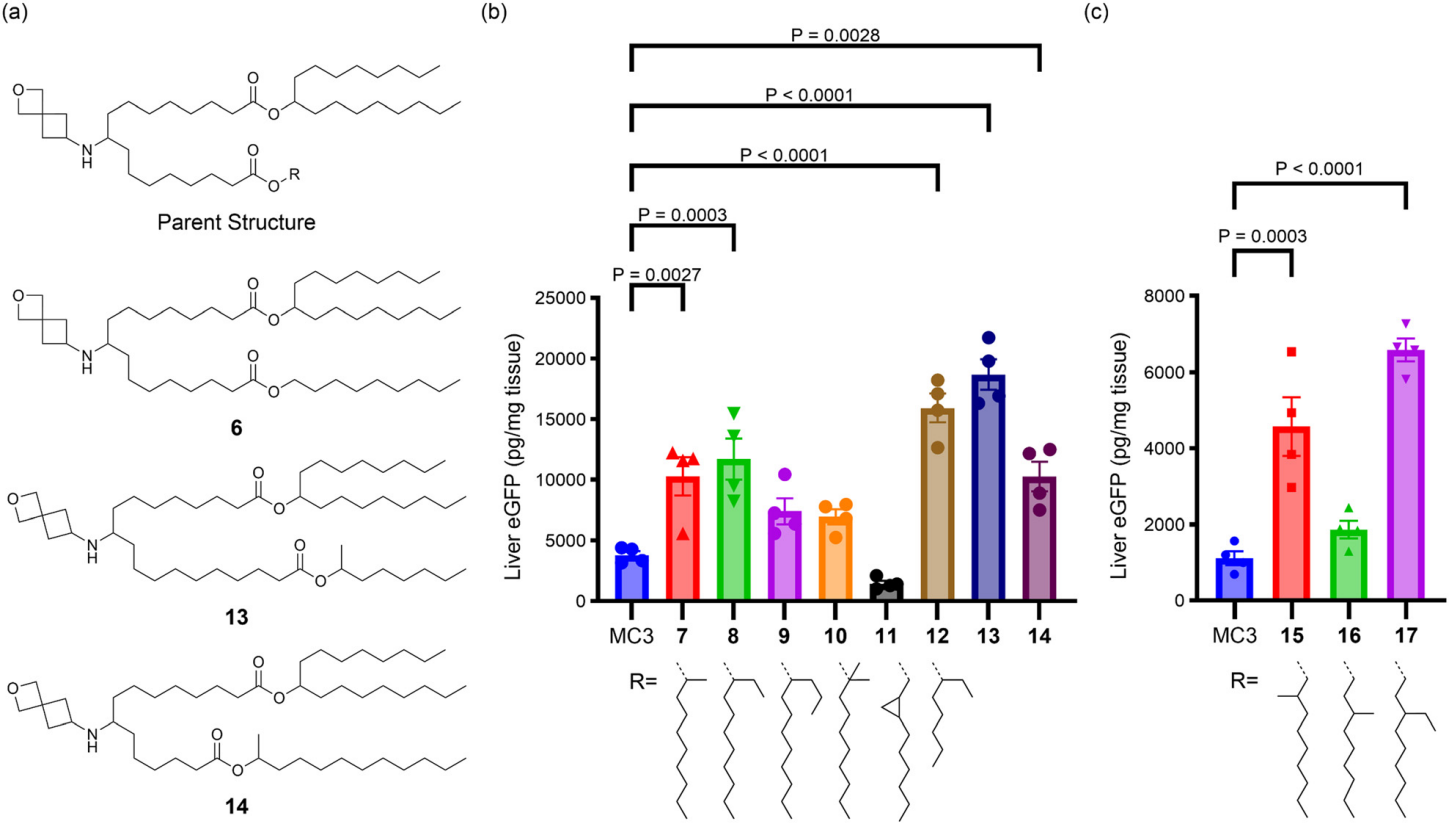
(a) The parent structure of the asymmetrical tail lipids that were tested, along with the structures of 6, 13, and 14. (b and c) The effects of additional branching and changing alcohol and linker length on protein expression in liver following systemic administration of eGFP mRNA-LNPs in mice are shown. For (b and c), mice (*N* = 4, BALB/c mice per group) were injected i.v. with 0.3 mg kg^−1^ eGFP mRNA LNPs with terminal collection of liver samples at 24 h post-dose. eGFP levels were quantified by ELISA. Error bars are the standard error of the mean (S.E.M.) and statistical comparisons were based on a one-way ANOVA with Dunnett's test for multiple testing correction comparing each lipid with MC3 evaluated concurrently in the same experiment.

The linker portion of the lipid joins the head group and the tail regions, and variation in linker length was the first SAR studied. As shown in Fig. 2a, lipids with two identical tails derived from 3-pentyloctan-1-ol gave similar results for linker lengths of *n* = 1 (2), 3 (1), and 4 (3), with all three performing significantly better than the MC3 comparator, demonstrating that this type of lipid with symmetric tails formed from a branched alcohol was relatively insensitive to linker length over the range *n* = 1–4. In contrast, lipids with tails formed from 2 different alcohols (Fig. 2b), the straight-chain primary 1-nonanol and the branched secondary heptadecan-9-ol, showed superior activity when the linker was shorter, as in lipid 4 (*n* = 1) (*P* < 0.0001). However, lipids with longer linkers, such as lipid 5 (*n* = 2) and lipid 6 (*n* = 3, see Fig. 3a and S1†) were much less effective. Because the lipids in both Fig. 2a and b share the same head group and have a total of 26 carbon atoms in the tail region, the effect of linker length variation on hydrophobicity was similar between both sets of compounds, suggesting that the difference in behaviour between the symmetric and asymmetric tail lipids was not driven by this property. Notably, the symmetric tail lipids had an additional branching point in the tail in comparison to the asymmetric tail lipids, and thus we reasoned that having fewer branching points in the tail makes the asymmetric tail lipid packing more sensitive to the effect of increasing linker length.

The importance and role of branching points in the tail alcohols was further explored to improve the activity of asymmetric tail lipids with longer linkers (*n* > 1). As shown in Fig. 3 for lipids 7–17, the R-group was varied while holding the heptadecan-9-ol tail, the linker length (equivalent to *n* = 3 in Fig. 2), and head group constant. In contrast to lipid 6, where R was an unsubstituted nonyl chain and which demonstrated low *in vivo* activity relative to MC3 (Fig. S1†), lipid 7 with a methyl group and lipid 8 with an ethyl group in the 1-position of the nonyl chain outperformed the MC3 benchmark (3-fold expression *vs.* MC3, *P* = 0.0027 and 0.0003, respectively), whereas a propyl group in this position (9) did not lead to a statistically significant improvement over the benchmark. As shown in Fig. 3c, lipid 15 with the methyl group in the 2-position of the nonyl chain was significantly better than the MC3 comparator (4-fold expression *vs.* MC3, *P* = 0.0003), but 16 with the methyl group in the 3-position was not. However, replacing the methyl with an ethyl group in the 3-position led to an improved lipid, 17 (6-fold expression *vs.* MC3, *P* < 0.0001). Addition of *gem*-dimethyl substitution at the 1-position (10) or a fused cyclopropyl ring connected at the 2- and 3-positions (11) did not lead to lipids that were more active than the benchmark. Taken together, these observations highlight that adding a branching point to the unsubstituted nonyl chain of lipid 6 has the potential to greatly improve the activity of lipids with linkers of *n* > 1 (as n is defined in Fig. 2). A methyl group was most effective in the 1- or 2-position of the nonyl chain, but not as effective in the 3-position, whereas an ethyl group appeared effective in both the 1- and 3-positions based on the lipids tested.

In addition to looking at the placement of the carbon branching, we were interested to probe how sensitive nonsymmetric tail lipids were to alcohol chain length. In lipid 12, it was observed that the use of 1-methyl-hexan-1-ol led to a potent lipid (4-fold increase of expression *vs.* MC3, *P* < 0.0001). To explore this approach further, lipids 13 and 14 were synthesized with nonidentical linker lengths allowing for the alcohol length to be modulated within compounds that are structural isomers of lipids 7, 15, and 16. The synthesis of 13 and 14 required an alternative synthetic approach that is described in the ESI.† As compared to lipid 7, in lipid 13 a C_2_H_4_ fragment is moved from the tail to the linker region, whereas in lipid 14, a C_2_H_4_ fragment is moved from the linker to the tail region. Compared to MC3, both 13 (5-fold increase in expression *vs.* MC3, *P* < 0.0001) and 14 (3-fold increase in expression *vs.* MC3, *P* = 0.0028) performed well, with 13 trending toward higher protein expression. These results suggest varying linker and alcohol tail length simultaneously may be a strategy to further improve lipid activity. Additionally, variation of the alcohol tail length may be a strategy to control lipid biodegradability as it has been reported that the combination of a longer linker and shorter alcohol tail can lead to more rapid lipid biodegradations, a property that is associated with improved safety.^16,22^

**Fig. 4:**
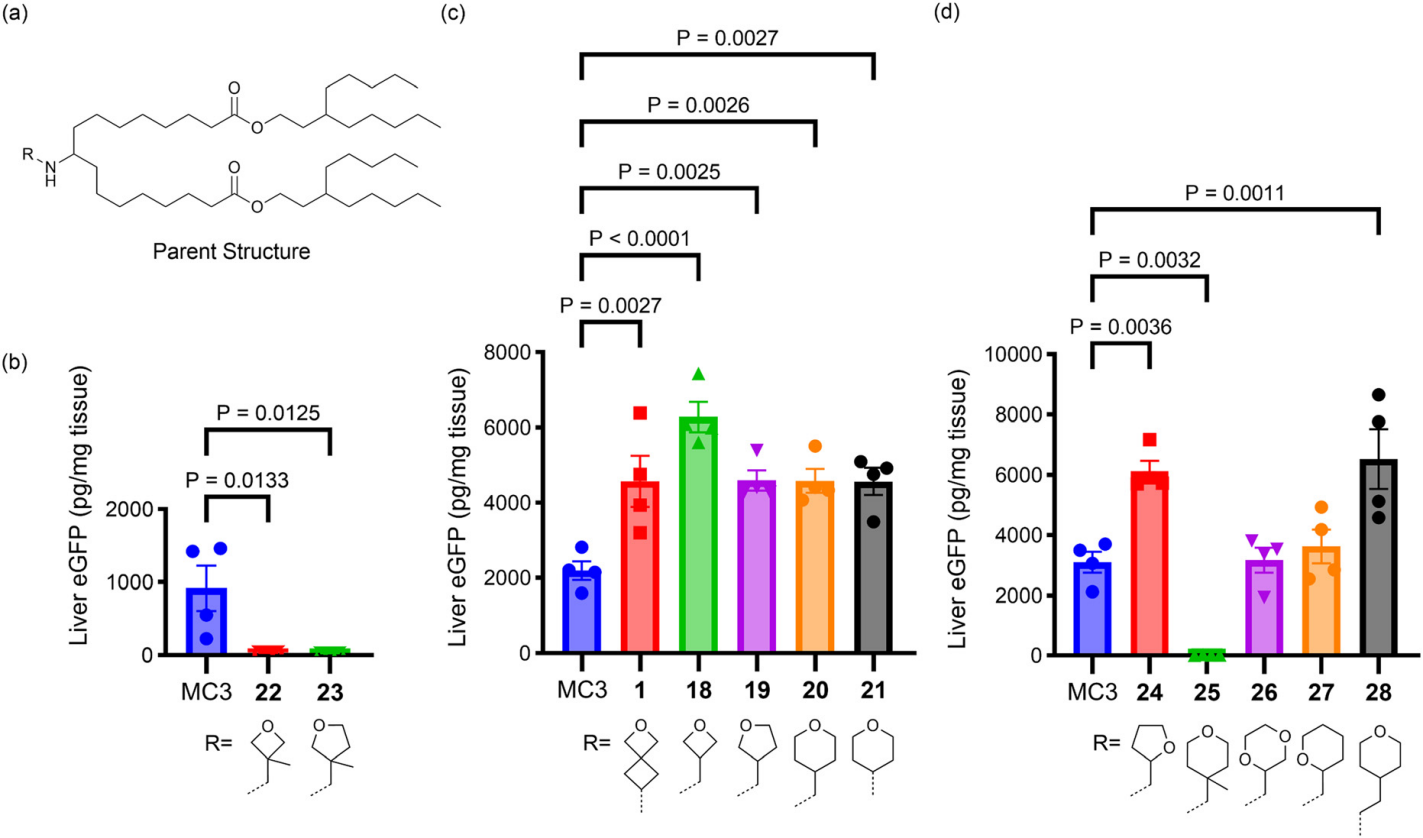
(a) The parent structure of the lipids tested. (b–d) The effects of head group structure on protein expression in liver following systemic administration of eGFP mRNA-LNPs in mice are shown. For (b–d), mice (*N* = 4, BALB/c mice per group) were injected i.v. with 0.3 mg kg^−1^ eGFP mRNA LNPs with terminal collection of liver samples at 24 h post-dose. eGFP levels were quantified by ELISA. Error bars are the standard error of the mean (S.E.M.) and statistical comparisons were based on a one-way ANOVA with Dunnett's test for multiple testing correction comparing each lipid with MC3 evaluated concurrently in the same experiment.

Having seen the importance of both tail and linker structure on lipid performance, our attention turned to understanding how varying head group structure influenced lipid activity while holding the linker and tails constant, as shown in Fig. 4 (lipids 1, 18–28). All of the head groups investigated had an oxygen-containing, saturated heterocycle ranging in ring size from four to six, with each ring size having examples of lipids with improved activity relative to the MC3 benchmark. In addition to the parent 2-oxaspiro[3.3]heptan-6-amine head group (1), an oxetane connected to the amine nitrogen through a methylene spacer at the 3-position (18) was also effective, suggesting that the rigidity imparted by the bispiro linkage was not essential for the lipid activity. Furthermore, lipids with five (19, 24)- and six (20, 21, 28)-member cyclic ethers displayed efficient mRNA delivery. On the other hand, some head groups led to lower activity. For example, when the nitrogen was connected to a tetrahydropyranyl (27) or a 1,4-dioxanyl (26) ring at the 2-position *via* a methylene linker the resulting lipids did not outperform MC3. A dramatic loss in activity was observed with methyl substitution to form a quaternary carbon at the connecting position of the head group ring, as in 22, 23, and 25 in comparison to the non-methylated parent structures 18, 19, and 20, respectively. As the electronic effect of methyl substitution on amine basicity is expected to be relatively minor, we propose that the effect of methyl substitution on head group size and hydrophobicity are responsible for the decreased lipid performance.

The *in vivo* evaluation of our novel lipids measured protein production at a single time point (24 h), which was chosen for our SAR studies based on known mRNA-LNP expression kinetics to report on the period of greatest protein translation.^34^ However, future studies exploring the time course of protein expression, the ability to delivery diverse mRNA cargoes, and biodistribution will be needed to fully understand how LNPs prepared with the lead lipids reported here compare to other high-performing LNP formulations.

In order to gain insight on lipid safety, mechanism of action, and performance when given by other routes of administration, we explored selected lipids in additional experiments. The *in vitro* cytotoxicity of LNPs prepared from lipids 8, 18, 20, and 22 were compared to MC3 LNPs using a lactate dehydrogenase (LDH)-based assay in a Huh-7-derived cell line (Fig. S2†). In the dose range tested (0.25–1.0 μg mL^−1^ mRNA), all of the LNPs had a minimal effect on cell viability, with cell viabilities above 80% at all doses. None of the novel lipids showed increased cytotoxicity relative to MC3. We also sought to use *in vitro* studies to shed light on why some of our best performing lipids, such as 8 and 18, outperformed the MC3 benchmark, whereas other lipids, such as 22, performed poorly. Although *in vitro* measures such as protein production or LNP uptake often fail to strongly correlate with *in vivo* activity, choosing the right cell models and using multiparametric measurements are essential for improving this correlation.^32^ While *in vitro* models are useful for comparing different LNP chemistries, they can lead to weak correlations with *in vivo* results and possibly misleading conclusions if only a few factors are considered in assessing correlation efficiency. However, it has been proposed that LNPs demonstrating improved endosomal escape efficiency could lead to higher *in vivo* potency.^35^ Therefore, we used a Huh-7 cell line engineered to express mCherry-Galectin 9 (GAL9) to monitor the ability of LNPs to induce endosomal membrane disruption by observing GAL9 puncta formation using confocal fluorescence microscopy.^36^ In order to normalize the degree of endosomal disruption to the amount of LNP internalized, the mRNA was labeled with a Cy5-fluorophore and the ratio of GAL9 puncta to Cy5 puncta was quantified. LNPs prepared using the lipids 8 and 18 demonstrated significantly higher levels of endosomal disruption in agreement with their superiority over MC3 *in vivo*, whereas the poor performing lipid 22 was comparable to MC3 (Fig. S3†). These results suggest that lipids 8 and 18 outperform MC3 *in vivo* at least in part due to an increased efficiency of endosomal membrane disruption and escape.

Finally, we compared LNPs prepared using lipids 1 and 20 to a benchmark SM-102 LNP when administered intramuscularly (Fig. S4†). As in the i.v.-dosed studies, the mRNA cargo expressed eGFP and the dose was 0.3 mg kg^−1^. After 24 hours, the amount of eGFP protein produced in the injected thigh muscle from LNPs made with 1 and 20 was similar to the SM-102 LNP, with the latter group trending higher. At the same time point, the liver expression of eGFP was significantly higher for SM-102 relative to 1, but was not statistically different from 20. These results suggest that the lead lipids identified in this work have the potential to be dosed *via* other routes of administration beyond i.v. and can effectively deliver mRNA to cells outside the liver.

## Conclusions

Our hypothesis that ionizable lipids based on a secondary amine could be optimized to match or outperform the delivery efficiency of a representative tertiary amine benchmark (MC3) was confirmed by our identification of multiple lead candidates. These lead secondary amine lipids demonstrated superior mRNA delivery and translation in the liver, highlighting their potential as high-performing alternatives to tertiary amine lipids. This finding marks an important step forward in expanding the chemical space of ionizable lipids. While these initial results are encouraging, further studies of these lipids will be required to understand whether secondary amine head groups have unique advantages relative to tertiary amines. The formation of *N*-nitroso amine impurities, which can be potent mutagens, is a concern for amine-containing compounds, especially secondary amines.^37^ Whether these impurities represent a significant concern for secondary amine ionizable lipids will depend on if they are formed to an appreciable extent during synthesis and LNP formulation and what levels are of concern given the LNP dosing amount and frequency.

The systematic design and testing of this library allowed us to draw several nonobvious lessons to inform the design of future candidates as summarized in Table 1. For example, we found that the activity of symmetrical tail lipids derived from identical branched alcohols was relatively insensitive to linker length over the range tested (*n* = 1–4), whereas lipids with one unbranched tail and one branched tail only showed good activity with the shorter linker length (*n* = 1). We suspected that addition of a branching point in the tail could rescue activity for longer linker length lipids (*n* > 1) and observed that methyl or ethyl substituents led to significantly improved activity. Finally, we observed that cyclic ether head groups with 4- to 6-membered ring sizes were tolerated and that the spirocyclic oxetane was not essential for activity. However, addition of a methyl group at the site of ring attachment led to much lower activity for all ring sizes tested. Taken together, these SAR observations highlight that ionizable lipid activity can be sensitive to relatively small changes in chemical structure. However, other structural modifications, such as changing linker length or varying the cyclic ether ring size were tolerated when examining lipids with symmetrical tails. Further understanding will likely require both additional biophysical characterization of lipid packing and ionization behavior, as well as improved LNP modeling to fully describe ionizable lipid SAR. Clinically-relevant mRNA cargos, as opposed to the tool mRNA we employed, may be more complex and challenging to deliver, but the highly active lipids identified are excellent candidates for further testing in mRNA-based therapies.

**Table 1:** Key SAR lessons

Property	SAR observation	Representative examples
Linker length	For symmetrical tail lipids, the linker lengths tested (*n* = 1–4) did not impact performance, with all leading to high protein expression *in vivo*	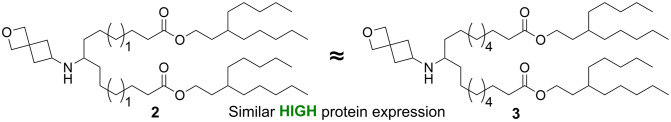
	For asymmetrical tail lipids, the shorter linker length (*n* = 1) outperformed longer linker lengths (*n* = 2–3) significantly	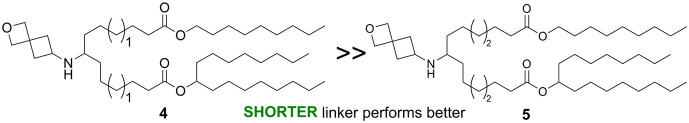
Tail branching	Asymmetrical tail lipids with additional branching outperformed lipids with an unbranched tail	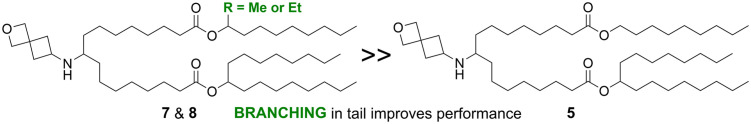
Head group size and branching	Cyclic ether ring sizes of 4 to 6 performed well, but the addition of a methyl group caused a dramatic loss in activity	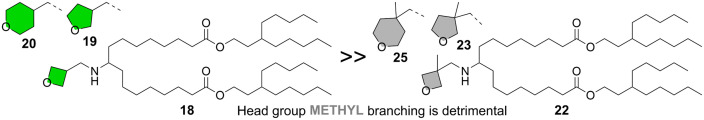

## Author contributions

E. L. D. contributed to writing and editing the manuscript, data curation, data analysis, and visualization. A. R. P. contributed to writing and editing the manuscript, investigation, methodology, and data analysis. M. H. and W. C. contributed to conceptualization and writing – review & editing. L. Z. contributed to supervision, data analysis and writing – review & editing. A. B. contributed to conceptualization, supervision and writing – review & editing.

## Conflicts of interest

All authors were at the time of the study employees of AstraZeneca and A. R. P., M. H., and W. C. are inventors on a patent application (WO 2023/089522 (A1)) related to this work.

## Supplementary Material

MD-016-D5MD00115C-s001

## Data Availability

The data supporting this article have been included as part of the manuscript or included in the ESI.†

## References

[cit1] Huang X., Kong N., Zhang X., Cao Y., Langer R., Tao W. (2022). Nat. Med..

[cit2] Barbier A. J., Jiang A. Y., Zhang P., Wooster R., Anderson D. G. (2022). Nat. Biotechnol..

[cit3] Pastor F., Berraondo P., Etxeberria I., Frederick J., Sahin U., Gilboa E., Melero I. (2018). Nat. Rev. Drug Discovery.

[cit4] Collén A., Bergenhem N., Carlsson L., Chien K. R., Hoge S., Gan L.-M., Fritsche-Danielson R. (2022). Nat. Rev. Drug Discovery.

[cit5] Davies J. C., Polineni D., Boyd A. C., Donaldson S., Gill D. R., Griesenbach U., Hyde S. C., Jain R., McLachlan G., Mall M. A., Alton E. W. (2024). Am. J. Respir. Crit. Care Med..

[cit6] Parayath N. N., Stephan S. B., Koehne A. L., Nelson P. S., Stephan M. T. (2020). Nat. Commun..

[cit7] van Haasteren J., Li J., Scheideler O. J., Murthy N., Schaffer D. V. (2020). Nat. Biotechnol..

[cit8] Yanez Arteta M., Kjellman T., Bartesaghi S., Wallin S., Wu X., Kvist A. J., Dabkowska A., Székely N., Radulescu A., Bergenholtz J., Lindfors L. (2018). Proc. Natl. Acad. Sci. U. S. A..

[cit9] Cullis P. R., Felgner P. L. (2024). Nat. Rev. Drug Discovery.

[cit10] Hou X., Zaks T., Langer R., Dong Y. (2021). Nat. Rev. Mater..

[cit11] Zhang Y., Sun C., Wang C., Jankovic K. E., Dong Y. (2021). Chem. Rev..

[cit12] Qiu M., Tang Y., Chen J., Muriph R., Ye Z., Huang C., Evans J., Henske E. P., Xu Q. (2022). Proc. Natl. Acad. Sci. U. S. A..

[cit13] Hassett K. J., Benenato K. E., Jacquinet E., Lee A., Woods A., Yuzhakov O., Himansu S., Deterling J., Geilich B. M., Ketova T., Mihai C., Lynn A., McFadyen I., Moore M. J., Senn J. J., Stanton M. G., Almarsson Ö., Ciaramella G., Brito L. A. (2019). Mol. Ther.--Nucleic Acids.

[cit14] Jayaraman M., Ansell S. M., Mui B. L., Tam Y. K., Chen J., Du X., Butler D., Eltepu L., Matsuda S., Narayanannair J. K., Rajeev K. G., Hafez I. M., Akinc A., Maier M. A., Tracy M. A., Cullis P. R., Madden T. D., Manoharan M., Hope M. J. (2012). Angew. Chem., Int. Ed..

[cit15] Ansell S. M. and Du X., US10166298B2, 2016

[cit16] Sabnis S., Kumarasinghe E. S., Salerno T., Mihai C., Ketova T., Senn J. J., Lynn A., Bulychev A., McFadyen I., Chan J., Almarsson Ö., Stanton M. G., Benenato K. E. (2018). Mol. Ther..

[cit17] Akinc A., Maier M. A., Manoharan M., Fitzgerald K., Jayaraman M., Barros S., Ansell S., Du X., Hope M. J., Madden T. D., Mui B. L., Semple S. C., Tam Y. K., Ciufolini M., Witzigmann D., Kulkarni J. A., van der Meel R., Cullis P. R. (2019). Nat. Nanotechnol..

[cit18] Polack F. P., Thomas S. J., Kitchin N., Absalon J., Gurtman A., Lockhart S., Perez J. L., Pérez Marc G., Moreira E. D., Zerbini C., Bailey R., Swanson K. A., Roychoudhury S., Koury K., Li P., Kalina W. V., Cooper D., Frenck, Jr. R. W., Hammitt L. L., Türeci Ö., Nell H., Schaefer A., Ünal S., Tresnan D. B., Mather S., Dormitzer P. R., Şahin U., Jansen K. U., Gruber W. C. (2020). N. Engl. J. Med..

[cit19] Baden L. R., El Sahly H. M., Essink B., Kotloff K., Frey S., Novak R., Diemert D., Spector S. A., Rouphael N., Creech C. B., McGettigan J., Khetan S., Segall N., Solis J., Brosz A., Fierro C., Schwartz H., Neuzil K., Corey L., Gilbert P., Janes H., Follmann D., Marovich M., Mascola J., Polakowski L., Ledgerwood J., Graham B. S., Bennett H., Pajon R., Knightly C., Leav B., Deng W., Zhou H., Han S., Ivarsson M., Miller J., Zaks T. (2021). N. Engl. J. Med..

[cit20] Finn J. D., Smith A. R., Patel M. C., Shaw L., Youniss M. R., van Heteren J., Dirstine T., Ciullo C., Lescarbeau R., Seitzer J., Shah R. R., Shah A., Ling D., Growe J., Pink M., Rohde E., Wood K. M., Salomon W. E., Harrington W. F., Dombrowski C., Strapps W. R., Chang Y., Morrissey D. V. (2018). Cell Rep..

[cit21] Xu Y., Golubovic A., Xu S., Pan A., Li B. (2023). J. Mater. Chem. B.

[cit22] Maier M. A., Jayaraman M., Matsuda S., Liu J., Barros S., Querbes W., Tam Y. K., Ansell S. M., Kumar V., Qin J., Zhang X., Wang Q., Panesar S., Hutabarat R., Carioto M., Hettinger J., Kandasamy P., Butler D., Rajeev K. G., Pang B., Charisse K., Fitzgerald K., Mui B. L., Du X., Cullis P., Madden T. D., Hope M. J., Manoharan M., Akinc A. (2013). Mol. Ther..

[cit23] Whitehead K. A., Dorkin J. R., Vegas A. J., Chang P. H., Veiseh O., Matthews J., Fenton O. S., Zhang Y., Olejnik K. T., Yesilyurt V., Chen D., Barros S., Klebanov B., Novobrantseva T., Langer R., Anderson D. G. (2014). Nat. Commun..

[cit24] Qiu M., Li Y., Bloomer H., Xu Q. (2021). Acc. Chem. Res..

[cit25] Holland R., Lam K., Jeng S., McClintock K., Palmer L., Schreiner P., Wood M., Zhao W., Heyes J. (2024). ACS Nano.

[cit26] Philipp J., Dabkowska A., Reiser A., Frank K., Krzysztoń R., Brummer C., Nickel B., Blanchet C. E., Sudarsan A., Ibrahim M., Johansson S., Skantze P., Skantze U., Östman S., Johansson M., Henderson N., Elvevold K., Smedsrød B., Schwierz N., Lindfors L., Rädler J. O. (2023). Proc. Natl. Acad. Sci. U. S. A..

[cit27] Hashiba K., Sato Y., Taguchi M., Sakamoto S., Otsu A., Maeda Y., Shishido T., Murakawa M., Okazaki A., Harashima H. (2023). Small Sci..

[cit28] Zhang X., Zhao W., Nguyen G. N., Zhang C., Zeng C., Yan J., Du S., Hou X., Li W., Jiang J., Deng B., McComb D. W., Dorkin R., Shah A., Barrera L., Gregoire F., Singh M., Chen D., Sabatino D. E., Dong Y. (2020). Sci. Adv..

[cit29] Cornebise M., Narayanan E., Xia Y., Acosta E., Ci L., Koch H., Milton J., Sabnis S., Salerno T., Benenato K. E. (2022). Adv. Funct. Mater..

[cit30] Pearson R. G., Vogelsong D. C. (1958). J. Am. Chem. Soc..

[cit31] Wuitschik G., Carreira E. M., Wagner B., Fischer H., Parrilla I., Schuler F., Rogers-Evans M., Müller K. (2010). J. Med. Chem..

[cit32] Bak A., Zhou L., Rejman J., Yanez Arteta M., Nilsson G., Ashford M. (2025). Expert Opin. Drug Delivery.

[cit33] Schoenmaker L., Witzigmann D., Kulkarni J. A., Verbeke R., Kersten G., Jiskoot W., Crommelin D. J. A. (2021). Int. J. Pharm..

[cit34] Pardi N., Tuyishime S., Muramatsu H., Kariko K., Mui B. L., Tam Y. K., Madden T. D., Hope M. J., Weissman D. (2015). J. Controlled Release.

[cit35] Chatterjee S., Kon E., Sharma P., Peer D. (2024). Proc. Natl. Acad. Sci. U. S. A..

[cit36] Munson M. J., O'Driscoll G., Silva A. M., Lázaro-Ibáñez E., Gallud A., Wilson J. T., Collén A., Esbjörner E. K., Sabirsh A. (2021). Commun. Biol..

[cit37] Tuesuwan B., Vongsutilers V. (2021). J. Pharm. Sci..

