# Supplementary material for: New ionizable lipids for non-viral mRNA delivery with secondary amine cyclic ether head groups
Source: RSC Med Chem. 2025 May 27;16(7):3273–80. doi: 10.1039/d5md00115c (PMC12107392; doi:10.1039/d5md00115c)

Supplementary Information (SI) for RSC Medicinal Chemistry.
This journal is © The Royal Society of Chemistry 2025

Supplementary Information for:

New ionizable lipids for non-viral mRNA delivery with secondary amine cyclic ether head groups

Eric L Dane,*[‡a] Aditya R Pote,[‡a] Martin Hemmerling,[b] Werngard Czechtizky,[b] Liping Zhou,[a] and Annette Bak[a]

[a.] *Advanced Drug Delivery, Pharmaceutical Sciences, R&D, AstraZeneca, Boston, MA, USA.*

[b.] *Medicinal Chemistry, Research and Early Development, Respiratory and Immunology, BioPharmaceuticals R&D, AstraZeneca, Gothenburg, Sweden.*

**Email:* eric.dane@astrazeneca.com

[‡] E.L.D and A.R.P contributed equally.

Table of Contents

Section	*Pages*
Table S1	2
Figure S1	3
Figure S2	4
Figure S3	5
Figure S4	6
LNP Experimental Methods	7-8
Synthetic Methods	9-41
References	41
^{1}H and HSQC NMR Spectra	42-69

Table S1. Characterization of LNP Encapsulation Efficiency (%EE) and Size

Figure	Compound	%EE	D_Z (nm)	D_N (nm)
2a	**MC3**	98	79	63
	**1**	91	68	52
	**2**	93	66	49
	**3**	95	77	61
2b	**MC3**	98	88	71
	**4**	92	75	57
	**5**	94	71	52
3b	**MC3**	98	74	61
	**7**	95	72	58
	**8**	95	70	56
	**9**	97	65	51
	**10**	96	65	50
	**11**	95	67	53
	**12**	93	73	54
	**13**	93	77	62
	**14**	96	73	58
3c	**MC3**	98	82	66
	**15**	95	90	63
	**16**	94	84	66
	**17**	97	80	65
4c	**MC3**	99	86	72
	**1**	97	84	68
	**18**	99	70	54
	**19**	99	79	58
	**20**	96	81	62
	**21**	96	73	58
4b	**MC3**	98	88	71
	**22**	97	78	63
	**23**	93	82	65
4d	**MC3**	98	79	63
	**24**	95	76	61
	**25**	92	81	63
	**26**	93	81	61
	**27**	87	77	59
	**28**	94	77	54

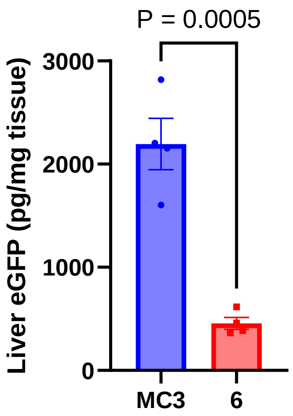

Figure S1. Protein expression in liver following systemic administration of eGFP mRNA-LNPs prepared using MC3 or lipid **6** in mice are shown. Mice (N = 4, BALB/c mice per group) were injected i.v. with 0.3 mg kg^{-1} eGFP mRNA LNPs with terminal collection of liver samples at 24 h post-dose. eGFP levels were quantified by ELISA. Error bars are the standard error of the mean (S.E.M.) and statistical comparisons were based on an unpaired t test.

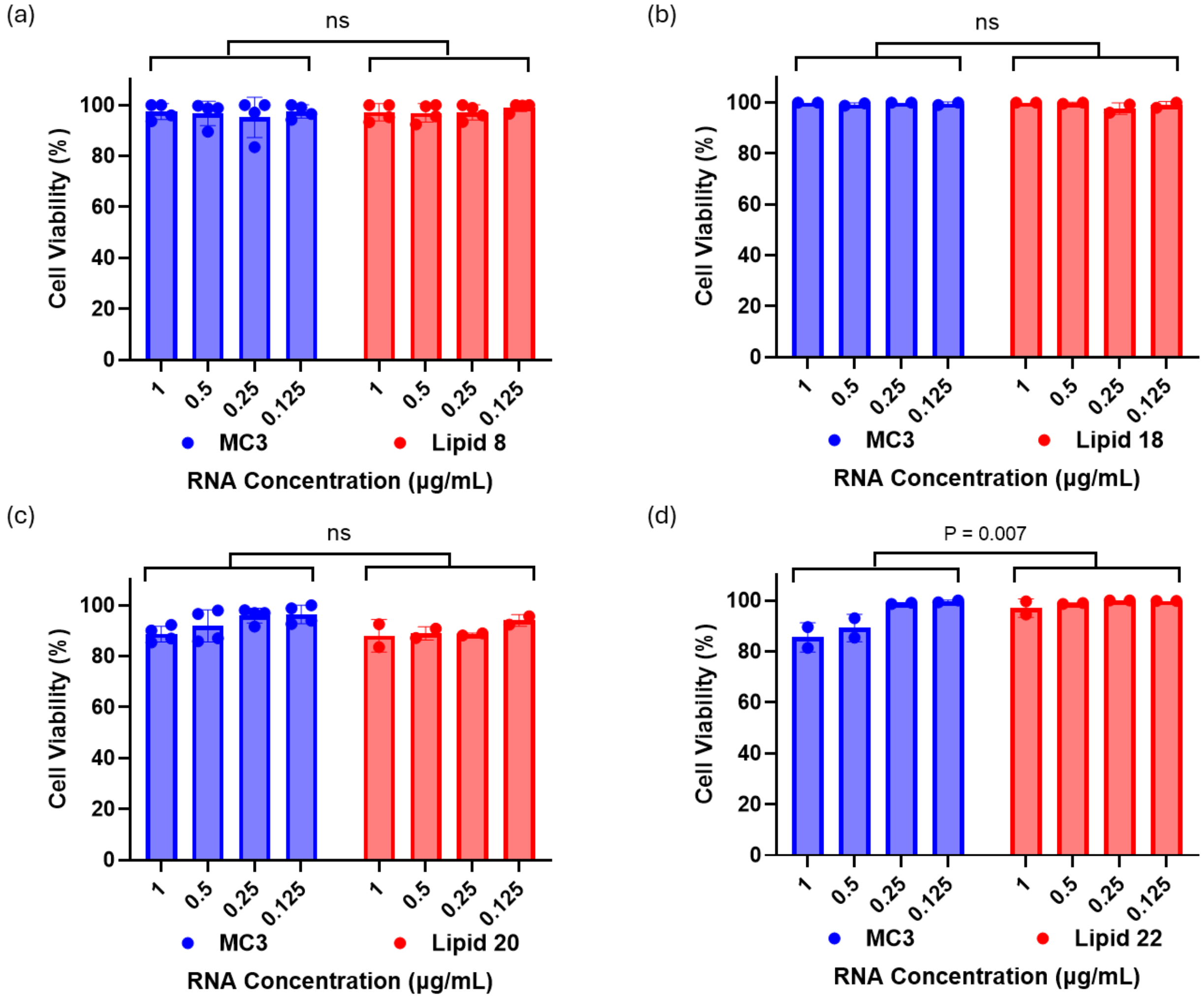

Figure S2. The effects of LNPs prepared using lipids **8**, **18**, **20**, and **22** on cell viability in comparison to MC3 LNPs were measured in Huh-7-mCherry-Galectin 9 cells using a lactate dehydrogenase (LDH) based cytotoxicity assay, as shown in graphs a.-d. Under these conditions, both the benchmark MC3 and the new lipid LNPs showed minimal effects on cell viability after 24 hours. Each data point represents a unique formulation prepared using the NanoFormHT formulation platform. Each lipid is compared to an MC3 benchmark LNP prepared and tested concurrently in the same assay. Additional experimental details are provided in the methods section. Error bars are the standard deviation (S.D.) and statistical comparisons were based on an ordinary two-way ANOVA.

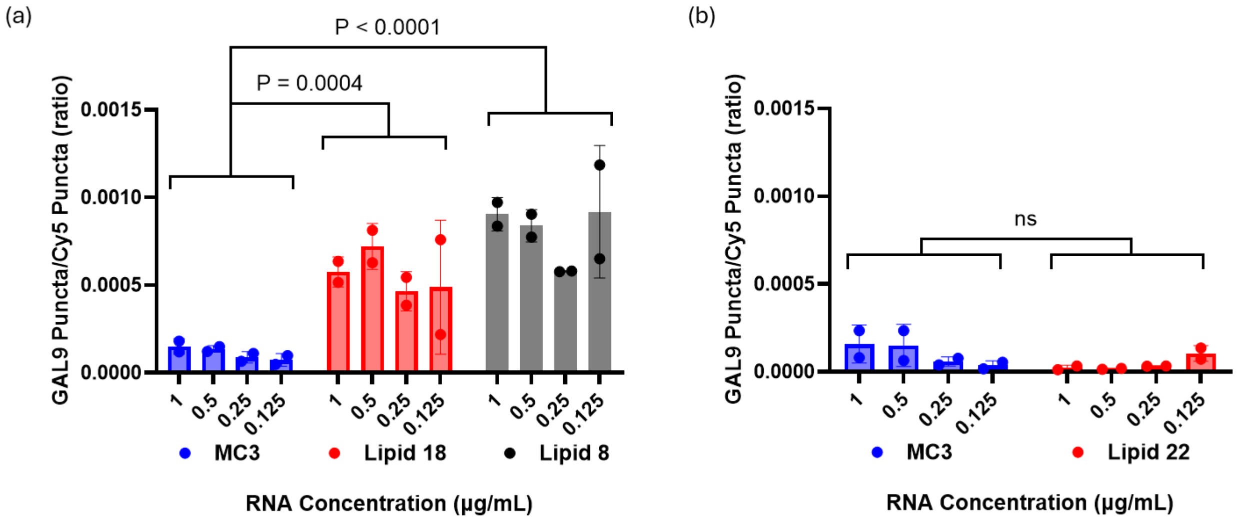

Figure S3. The ability of LNPs prepared using lipids **8** and **18** (a) or lipid **22** (b) encapsulating a Cy5-labled mRNA to induce endosomal disruption in comparison to MC3 LNPs were measured in Huh-7 cells expressing an mCherry-Galectin 9 (GAL9) reporter protein. In order to normalize the effect of endosomal disruption to the amount of LNP uptake, we report the ratio of GAL9 puncta, which are indicative of endosomal membrane damage, to Cy5 puncta, which are proportional to the amount of Cy5-mRNA internalized, which were measured via confocal fluorescence microscopy. Lipids **8** and **18**, which performed well *in vivo*, showed higher levels of endosomal disruption as compared to MC3 tested concurrently. In contrast, Lipid **22**, which performed very poorly in vivo, displayed low levels of endosomal disruption. Each data point represents a unique formulation prepared using the NanoFormHT formulation platform. Additional experimental details are provided in the methods section. Error bars are the standard deviation (S.D.) and statistical comparisons were based on an ordinary two-way ANOVA comparing each lipid with MC3 with Dunnett's test for multiple testing correction.

(a)

LNP composition	Ionizable lipid	N/P ratio	PEG lipid	%EE	D_Z (nm)	D_N (nm)
Ionizable lipid/cholesterol /DSPC/PEG lipid (50/38.5/10/1.5 mol%)	SM-102	6:1	DMG-PEG2k	98	73	49
	**1**	3:1	DMPE-PEG2k	97	84	68
	**20**	3:1	DMPE-PEG2k	96	81	62

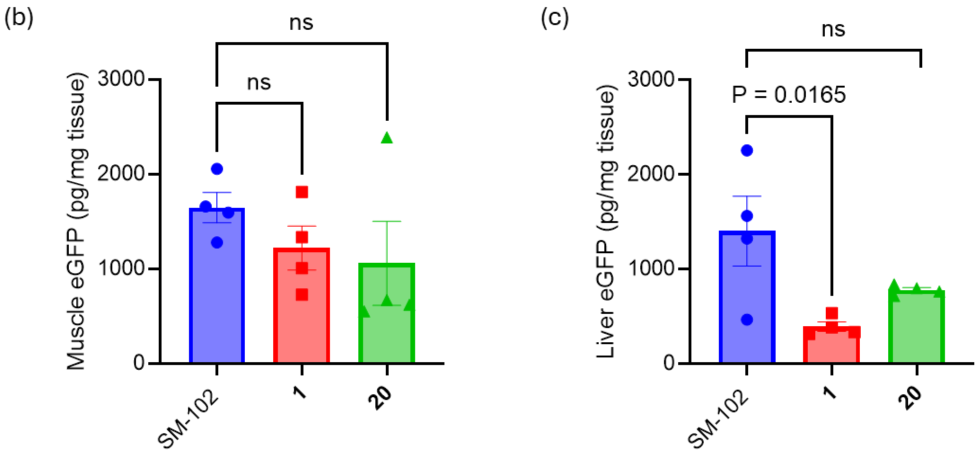

Figure S4. LNP formulations (a) prepared using SM-102, lipid **1**, and lipid **20** delivering eGFP-expressing mRNA were evaluated for protein expression in muscle tissue (b) and liver tissue (c) 24 hours after intramuscular administration. SM-102 LNPs were prepared using an N/P ratio of 6:1 and using 1.5 mol% DMG-PEG2k to approximate the composition used in SpikeVax® (mRNA-1273). For (b,c), mice (N = 4, BALB/c mice per group) were injected intramuscularly with 0.3 mg kg^{-1} eGFP mRNA LNPs with terminal collection of the injected muscle tissue and liver samples at 24 h post-dose. The eGFP levels were quantified by ELISA. Error bars are the standard error of the mean (S.E.M.) and statistical comparisons were based on a one-way ANOVA with Dunnett's test for multiple testing correction comparing each lipid with SM-102.

LNP Experimental Methods

Materials

The benchmark lipids DLin-MC3-DMA and SM-102 were synthesized by AstraZeneca. The sources of additional materials are described in context below.

Lipid Nanoparticle Preparation

The four different lipid components: ionizable lipid; cholesterol (Sigma-Aldrich); DSPC (distearoyl phosphatidyl choline, Avanti Polar Lipids Inc); and DMPE-PEG2k (PM-020CN, NOF America Corporation) were dissolved in ethanol (EtOH) and combined. The ratio of lipids in all experiments was ionizable lipid/cholesterol/DSPC/PEG lipid (50/38.5/10/1.5 mol%). The total concentration of lipids in ethanol was 12.5 mM. A solution of eGFP mRNA (TriLink Biotechnologies) was prepared in 50 mM citrate buffer (pH 3). The mRNA and lipid solutions were mixed in a NanoAssemblr (Precision Nanosystems, Vancouver, BC, Canada) microfluidic mixing system at a mixing ratio of Aq:EtOH = 3:1 and a constant flow rate of 12 mL/min. The mRNA in citrate buffer solution was prepared such that at the time of mixing the ratio between the nitrogen atoms on the ionizable lipid and phosphorus atoms on the mRNA chain (N/P ratio) was 3:1. The first 0.2-0.35 mL and the last 0.05-0.1 mL of the LNP suspension prepared were discarded. Formulated LNPs were dialyzed overnight in PBS (pH 7.4) at 4 °C.

Lipid Nanoparticle Characterization

The size of the mRNA lipid nanoparticles was determined by dynamic light scattering measurements using a Zetasizer Nano ZS from Malvern Instruments Ltd, giving directly the z-average particle diameter. The number-weighted particle size distributions and averages were calculated using a particle refractive index of 1.45. The final mRNA concentration and encapsulation efficiency percentage (%EE) was measured by Quant-it Ribogreen Assay Kit (ThermoFischer Scientific Inc.) using Triton X-100 to disrupt the LNPs. The mRNA encapsulation efficiency was determined according to the following equation: %EE = [1-(non-encapsulated mRNA/total mRNA)] x 100.

Animals

All the procedures related to animal handling, care, and the treatment were performed according to guidelines approved by the Institutional Animal Care and Use Committee (IACUC) of Pharmaron (Beijing) Inc., Beijing, P.R. China following the guidance of the Association for Assessment and Accreditation of Laboratory Animal Care (AAALAC). Female BALB/c mice were purchased (SPF (Beijing) Laboratory Animal Technology Co. Ltd.) and on arrival were caged in groups of 4 on corncob bedding, with normal diet and were provided tap drinking water ad libitum that was purified and autoclaved before being offered to the animal. The environment was maintained at a target temperature of 22 ± 3°C and relative humidity of 40-80%, with a 12-hour light/dark cycle. Animals were acclimatized to the housing conditions for at least 7 days prior to any experimental procedures and were approximately 6-8 weeks of age at the start of dosing. Animals were assigned to respective groups such that the mean body weight for each treatment group was identical with an N=4 for each treatment group.

LNP Administration and Tissue Collection

For all LNPs dosed intravenously: Each mouse was removed from its cage and restrained and then dosed with a slow IV bolus of a formulation in the lateral tail vein at a dose level of 0.3 mg/kg. All animals were sacrificed after 24 hours and the liver collected for further analysis. For all LNPs dosed intramuscularly: Each mouse was removed from the cage and restrained, and then dosed in the caudal thigh area by slowly

injecting a formulation into the muscle with a dose volume of 50 µL at a dose level of 0.3 mg/kg. Muscle surrounding the injection site and liver were collected for further analysis 24 hours following injection.

eGFP ELISA

Absolute quantification of eGFP protein was done using ELISA (GFP SimpleStep ELISA kit, Abcam #ab171581) according to the manufacturer's instruction.

Formulation of LNPs (NanoFormHT). For high-throughput formulation of nanoparticles, a 384-well source plate was prepared containing both mixed lipid solutions and mixed mRNA/buffer solutions using the Dragonfly liquid-handling robot dispenser (SPT Labtech). Lipid stock solutions were prepared in absolute ethanol, while the Cy5-labelled mRNA cargo (TriLink Biotechnologies) was prepared in 50 mM Citrate pH 3 buffer (TekNova). The lipid mixtures were prepared at a N/P ratio = 3:1. To formulate the nanoparticles, the ethanolic lipid solutions and aqueous mRNA solutions were mixed (1:3) using the Bravo liquid handling robot (VWorks software v12.2.0.1306) in conjunction with a custom 3D printed microfluidics device.[1] After the mixing process, all LNPs were diluted in a 1:1 ratio with PBS at pH 7.4.

NanoProfiler assay. Huh-7-mCherry-Galectin 9 reporter cells were established and validated internally.[2] Cells were maintained in a complete medium of DMEM+Glutamax (Gibco: 31966-021) + 10% FBS, at 37°C in a humidified incubator with 5% CO_2. For experiments, Huh-7 were actively growing and split directly into PhenoPlate 384-well (Revvity: 6057302) at 3500 cells/well. Cells were grown for 24 h and dosed with LNPs at mRNA concentrations (1, 0.5, 0.25 and 0.125 µg/ml of encapsulated mRNA) using an Echo (Labcyte) dispenser to directly transfer to well. Cells were incubated a further 24 h. At assay endpoint, supernatant was collected for the cell death assay. Cells were washed with PBS, fixed using paraformaldehyde 4.2%, and nuclei were counter-stained with Hoechst. Cells were then imaged on a CV7000 spinning disk confocal microscope (Yokogawa, Tokyo, Japan) in a humidified environmental chamber maintained at 37°C and supplemented with 5% CO2. The acquired images were analyzed using Columbus software (version 2.9.1.532, PerkinElmer) to segment cellular structures and determine cellular uptake and endosomal escape events. Data were normalized across independent screening experiments using internal controls.

Cell death assay. CyQUANT™ LDH Cytotoxicity Assay (Invitrogen: C20303) was used to determine the % of cell death post LNP treatment, according to manufacturer instructions. Briefly, 10 µL of cell supernatant at endpoint were mixed with 10 µL of resuspended reagent and incubated for 10 min at RT protected from light. 10 µL of ready-to-use stop solution was added. Fluorescence was measured by using an excitation of 560 nm and an emission of 590 nm. The percentage of cell death was calculated and expressed as a percentage of the positive control, which were non-treated lysed cells. Cell viability was obtained by subtracting the percentage of cell death from 100%.

Synthetic Methods and Compound Characterization

General Experimental Procedures: All reactions were performed in dry glassware fitted with rubber septa or screw cap under a positive pressure of argon or nitrogen, unless otherwise noted. Air- and moisture-sensitive liquids were transferred via syringe or stainless-steel cannula. Solutions were concentrated by rotary evaporation at or below 40°C. Commercial solvents and reagents were used as received, unless otherwise noted.

^{1}H NMR: 500 MHz; probe: 5mm Bruker Smart probe with ATM+Z PABBO 500S1-BBF-H-D; magnet: ASCEND™ 500; Console: AVANCE Neo 500; Auto Sampler: SampleXpress™60; software: Topspin 4. Proton chemical shifts are expressed in parts per million (ppm, δ scale) and are referenced to residual protium in the NMR solvent (Chloroform-*d*: δ 7.26, Methanol-*d*4: δ 3.31, DMSO-*d*6: δ 2.50). Data are represented as follows: chemical shift, multiplicity (s = singlet, d = doublet, t = triplet, q = quartet, dd = doublet of doublets, dt = doublet of triplets, m = multiplet, br = broad, app = apparent), integration, and coupling constant (*J*) in Hertz (Hz).

LCMS: UPLC-MS was carried out using a Waters Acquity UPLC and Waters SQD mass spectrometer (column temp 30°C, UV detection = 210-400nm, mass spec = ESI with positive/negative switching) at a flow rate of 1 mL/min using a solvent gradient of 2 to 98% B over 1.5 mins (total runtime with equilibration back to starting conditions 2 min), where A = 0.1% formic acid in water and B = 0.1% formic acid in acetonitrile (for acid work) or A = 0.1% ammonium hydroxide in water and B =acetonitrile (for base work). For acid analysis the column used was Waters Acquity HSS T3, 1.8 mm, 2.1 x 30 mm, for base analysis the column used was Waters Acquity BEH C18, 1.7 mm, 2.1 x 30mm.

Abbreviations

AcOH – Acetic acid
DCM – Dichloromethane
DMAP - N,N-dimethylpyridin-4-amine
DMSO - Dimethylsulfoxide
EtOAc - Ethyl acetate
EDC.HCl - 3-(((ethylimino)methylene)amino)-N,N-dimethylpropan-1-amine hydrochloride
1,2-DCE - 1,2-Dichloroethane
NMP - N-Methyl-2-pyrrolidone
MeOH - Methanol
RT - Room temperature
TEA - Triethylamine
TFAA - Trifluoroacetic anhydride
DIPEA - N-ethyl-N-isopropylpropan-2-amine

Synthesis of Lipids 1, 18-28

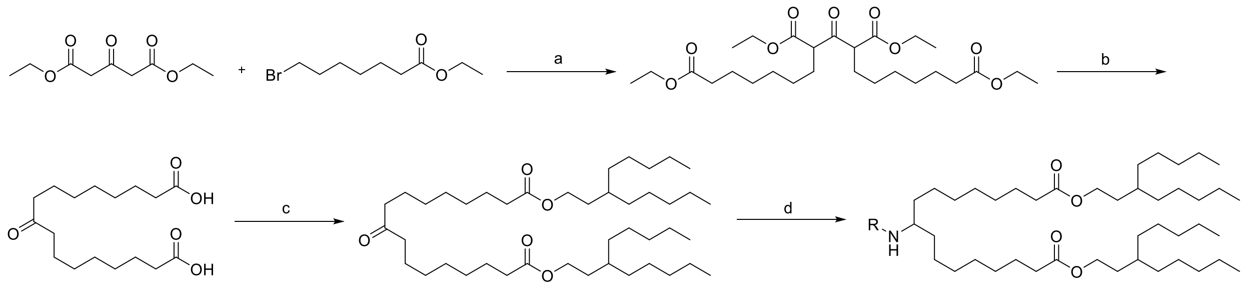

Reactants/Reagents: a) NaOEt, EtOH; b) conc. HCl, AcOH; c) 3-pentyloctan-1-ol, EDC.HCl, DIPEA, DMAP, DCM; d) R-NH_2, $NaBH(OAc)_3$, AcOH (as indicated), 1,2-DCE: NMP (4:1)

Step a) Sodium ethanolate (2.52 g, 37.09 mmol) was added portion-wise to a stirred solution of diethyl 3-oxopentanedioate (4.49 mL, 24.73 mmol) in absolute (99.5%) ethanol (14 mL) at 25°C over a period of 10 minutes under argon. The resulting suspension was stirred at 81 °C for 1 hour. To the reaction mixture ethyl 7-bromoheptanoate (12.05 mL, 61.82 mmol) was added dropwise and the suspension was stirred at 81 °C for an additional 18 hours. The reaction mixture was cooled to RT and concentrated under reduced pressure to dryness and redissolved in DCM (50 mL), and extracted 3 times with water (50 mL), and washed with sat. aq. NaCl (50 mL). The organic layer was dried over $MgSO_4$, filtered and concentrated under reduced pressure to afford crude product. The resulting residue was purified by flash silica chromatography, elution gradient 10 to 50% EtOAc in hexanes. Product fractions were concentrated under reduced pressure to dryness to afford **tetraethyl 8-oxopentadecane-1,7,9,15-tetracarboxylate** (8.10 g, 63.6%) as a pale-yellow oil. ^{1}H NMR (500 MHz, Chloroform-*d*) δ ppm 1.2 - 1.4 (m, 24 H) 1.5 - 1.9 (m, 8 H) 2.2 - 2.3 (m, 4 H) 3.4 - 3.5 (m, 2 H) 4.1 - 4.2 (m, 8 H).

Step b) Hydrogen chloride (33.3 mL, 406.06 mmol) was added slowly to a stirred solution of tetraethyl 8-oxopentadecane-1,7,9,15-tetracarboxylate (8.1 g, 15.74 mmol) in acetic acid (20 mL) at 25°C. The resulting solution was stirred at 102 °C for 18 hours w/ reflux condenser and an outlet to remove excess of HCl gas. The reaction was cooled to RT and poured on ice-water (50 mL) and was allowed to sit for 30 min. The precipitate was collected by filtration, washed with cold water (3 x 20 mL) and dried under vacuum to afford 9-oxoheptadecanedioic acid (crude) as a pale yellow solid. The crude product was purified by crystallization from acetone to afford **9-oxoheptadecanedioic acid** (1.381 g, 27.9%) as a white solid.[3] ^{1}H NMR (500 MHz, DMSO-*d*6) δ ppm 1.2 (br s, 12 H) 1.4 (dt, *J*=18.6, 6.7 Hz, 8 H) 2.2 (t, *J*=7.2 Hz, 4 H) 2.3 - 2.4 (m, 4 H) 11.8 - 12.1 (m, 2 H); $C_{17}H_{30}O_5$ *m/z* calcd. 314.209 observed 313.1 $[M-H]^-$ (LCMS).

Step c) 3-(((ethylimino)methylene)amino)-N,N-dimethylpropan-1-amine hydrochloride (690 mg, 3.60 mmol) was added in one portion to a stirred solution of 9-oxoheptadecanedioic acid (419 mg, 1.33 mmol), 3-pentyloctan-1-ol (641 mg, 3.20 mmol), N,N-dimethylpyridin-4-amine (32.6 mg, 0.27 mmol) and N-ethyl-N-isopropylpropan-2-amine (836 µl, 4.80 mmol) in DCM (20 mL) at 25°C under argon. The resulting solution was stirred at 25 °C for 50 hours. The reaction mixture was diluted with DCM (25 mL), water (10 mL) and sat. aq. NH_4Cl (10 mL). The layers were separated, and the aqueous layer was extracted three times with DCM (25 mL). The combined organic layers were dried over $MgSO_4$, filtered and concentrated under reduced pressure to dryness to afford crude product. The resulting residue was purified by flash silica chromatography, elution gradient 10 to 40% EtOAc in hexanes. Product fractions were concentrated under reduced pressure to dryness to afford **bis(3-pentyloctyl) 9-oxoheptadecanedioate** (0.820 g, 91%)

as a pale-yellow oil. ^{1}H NMR (500 MHz, Chloroform-*d*) δ ppm 0.9 - 0.9 (m, 12 H) 1.2 - 1.3 (m, 46 H) 1.5 - 1.7 (m, 12 H) 2.3 (t, *J*=7.6 Hz, 4 H) 2.4 (t, *J*=7.4 Hz, 4 H) 4.1 (t, *J*=7.1 Hz, 4 H).

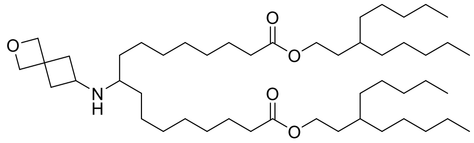

1

Lipid 1, Step d) Sodium triacetoxyhydroborate (193 mg, 0.91 mmol) was added in one portion to a stirred solution of 2-oxaspiro[3.3]heptan-6-aminium chloride (131 mg, 0.87 mmol) and bis(3-pentyloctyl) 9-oxoheptadecanedioate (247 mg, 0.36 mmol) in 1,2-DCE (6 mL) and NMP (1.5 mL) at 25°C under argon. The resulting solution was stirred at 25 °C for 30 hours. The reaction mixture was diluted with DCM (15 mL) and water (5 mL) with sat. aq. Na_2CO_3 (10 mL). The layers were separated, and the aqueous layer was extracted with DCM (3 x 20 mL). The combined organic layers were dried over $MgSO_4$, filtered and concentrated under reduced pressure to dryness to afford crude product. The resulting residue was purified by flash silica chromatography, elution gradient 0 to 40% of 20% MeOH/DCM (w/ 1% NH_4OH) in DCM. Product fractions were concentrated under reduced pressure to dryness to afford **1** (bis(3-pentyloctyl) 9-((2-oxaspiro[3.3]heptan-6-yl)amino)heptadecanedioate, 0.139 g, 49.2 %) as a colorless oil. ^{1}H NMR (500 MHz, Methanol-*d*4) δ ppm 0.9 (t, *J*=7.0 Hz, 12 H) 1.3 (br s, 52 H) 1.4 (br s, 2 H) 1.5 - 1.7 (m, 9 H) 2.0 (td, *J*=9.1, 2.8 Hz, 2 H) 2.3 - 2.3 (m, 4 H) 2.5 - 2.6 (m, 3 H) 3.2 (t, *J*=7.8 Hz, 1 H) 4.1 (t, J=6.7 Hz, 4 H) 4.6 (s, 2 H) 4.7 (s, 2 H); $C_{49}H_{93}NO_5$ *m/z* calcd. 776.285 observed 776.7 $[M+H]^+$ (LCMS).

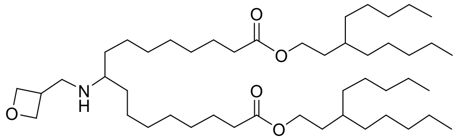

18

Lipid 18, Step d) Sodium triacetoxyhydroborate (65.2 mg, 0.31 mmol) was added in one portion to a stirred solution of bis(3-pentyloctyl) 9-oxoheptadecanedioate (69.6 mg, 0.10 mmol) and oxetan-3-ylmethanaminium chloride (38.0 mg, 0.31 mmol) in 1,2-DCE (2 mL) and NMP (0.5 mL) under argon. The resulting solution was stirred at 25 °C for 50 hours. The reaction mixture was diluted with DCM (15 mL), water (5 mL) and sat. aq. Na_2CO_3 (5 mL). The layers were separated, and the aqueous layer was extracted three times with DCM (15 mL). The combined organic layers were dried over $MgSO_4$, filtered and concentrated under reduced pressure to dryness to afford crude product. The resulting residue was purified by flash silica chromatography, elution gradient 0 to 50% of 20% of MeOH/DCM (w/ 1% NH_4OH) in DCM. Product fractions were concentrated under reduced pressure to dryness to afford **bis(3-pentyloctyl) 9-((oxetan-3-ylmethyl)amino)heptadecanedioate** (0.048 g, 62.8%) as a colorless oil. ^{1}H NMR (500 MHz, Methanol-*d*4) δ ppm 0.9 (t, *J*=7.1 Hz, 12 H) 1.3 - 1.4 (m, 48 H) 1.4 - 1.5 (m, 6 H) 1.6 - 1.7 (m, 8 H) 2.3 (t, *J*=7.4 Hz, 4 H) 2.5 (t, *J*=5.9 Hz, 1 H) 2.9 (d, *J*=7.5 Hz, 2 H) 3.1 - 3.1 (m, 1 H) 4.1 (t, *J*=6.8 Hz, 4 H) 4.4 (t, *J*=6.0 Hz, 2 H) 4.8 - 4.8 (m, 2 H); $C_{47}H_{91}NO_5$ *m/z* calcd. 749.690 observed 750.6 $[M+H]^+$ (LCMS).

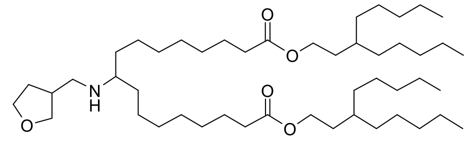

19

Lipid 19, Step d) Sodium triacetoxyhydroborate (32.9 mg, 0.16 mmol) was added in one portion to a stirred solution of bis(3-pentyloctyl) 9-oxoheptadecanedioate (58.6 mg, 0.09 mmol), (tetrahydrofuran-3-yl)methanamine (14.25 µl, 0.13 mmol) and acetic acid (181 µl, 0.18 mmol) in 1,2-DCE (2 mL) and NMP (0.5 mL) under argon. The resulting solution was stirred at 25 °C for 18 hours. The reaction mixture was diluted with DCM (15 mL), water (5 mL) and sat. aq. Na_2CO_3 (5 mL). The layers were separated, and the aqueous layer was extracted three times with DCM (15 mL). The combined organic layers were dried over $MgSO_4$, filtered and evaporated to dryness to afford crude product. The resulting residue was purified by flash silica chromatography, elution gradient 0 to 50% of 20% MeOH/DCM (w/ 1% NH_4OH) in DCM. Product fractions were concentrated under reduced pressure to dryness to afford **19** (bis(3-pentyloctyl) 9-(((tetrahydrofuran-3-yl)methyl)amino)heptadecanedioate, 0.017 g, 26.1%) as a colorless oil. ^{1}H NMR (500 MHz, Methanol-*d*4) δ ppm 0.9 (t, *J*=7.1 Hz, 12 H) 1.3 - 1.4 (m, 48 H) 1.4 - 1.5 (m, 6 H) 1.5 - 1.7 (m, 9 H) 2.0 - 2.1 (m, 1 H) 2.3 - 2.3 (m, 4H) 2.3 - 2.4 (m, 1 H) 2.5 (br t, *J*=5.9 Hz, 1 H) 2.6 - 2.6 (m, 2 H) 3.4 - 3.5 (m, 1 H) 3.7 - 3.8 (m, 1 H) 3.8 - 3.9 (m, 2 H) 4.1 (t, *J*=6.8 Hz, 4 H); $C_{48}H_{93}NO_5$ *m/z* calcd. 763.705 observed 764.8 $[M+H]^+$ (LCMS).

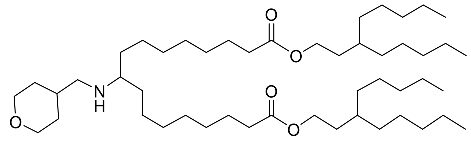

20

Lipid 20, Step d) Sodium triacetoxyhydroborate (35.2 mg, 0.17 mmol) was added in one portion (after 10 min) to a stirred solution of bis(3-pentyloctyl) 9-oxoheptadecanedioate (62.7 mg, 0.09 mmol), (tetrahydro-2H-pyran-4-yl)methanamine (15.64 µL, 0.14 mmol) and acetic acid (194 µL, 0.19 mmol) in 1,2-DCE (2 mL) and NMP (0.5 mL) under argon. The resulting solution was stirred at 25 °C for 40 hours. The reaction mixture was diluted with DCM (15 mL), water (5 mL) and sat. aq. Na_2CO_3 (5 mL). The layers were separated, and the aqueous layer was extracted three times with DCM (3 x 15 mL). The combined organic layers were dried over $MgSO_4$, filtered and concentrated under reduced pressure to dryness to afford crude product. The resulting residue was purified by flash silica chromatography, elution gradient 0 to 40% of 20% MeOH/DCM (w/ 1% NH_4OH) in DCM. Product fractions were concentrated under reduced pressure to dryness to afford **20** (bis(3-pentyloctyl) 9-(((tetrahydro-2H-pyran-4-yl)methyl)amino) heptadecanedioate, 0.034 g, 47.7%) as a colorless oil. ^{1}H NMR (500 MHz, Methanol-*d*4) δ ppm 0.9 (t, *J*=7.0 Hz, 12 H) 1.2 - 1.4 (m, 52 H) 1.4 - 1.5 (m, 6 H) 1.5 - 1.8 (m, 9 H) 2.3 (t, *J*=7.3 Hz, 4 H) 2.5 - 2.6 (m, 3 H) 3.4 - 3.5 (m, 2 H) 3.9 (dd, *J*=11.0, 4.0 Hz, 2 H) 4.1 (t, *J*=6.7 Hz, 4 H); $C_{49}H_{95}NO_5$ *m/z* calcd. 777.721 observed 778.7 $[M+H]^+$ (LCMS).

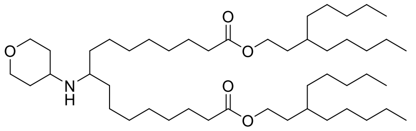

21

Lipid 21, Step d) Sodium triacetoxyhydroborate (78 mg, 0.37 mmol) was added in one portion (after 10 min) to a stirred solution of bis(3-pentyloctyl) 9-oxoheptadecanedioate (83 mg, 0.12 mmol) and tetrahydro-2H-pyran-4-aminium chloride (50.5 mg, 0.37 mmol) in 1,2-DCE (2 mL) and NMP (0.5 mL) under argon. The resulting solution was stirred at 25 °C for 50 hours. The reaction mixture was diluted with DCM (10 mL), water (5 mL) and sat. aq. Na_2CO_3 (5 mL). The layers were separated, and the aqueous layer was extracted with DCM (3 x 10 mL). The combined organic layers were dried over $MgSO_4$, filtered and concentrated under reduced pressure to dryness to afford crude product. The resulting residue was purified 2 times by flash silica chromatography, elution gradient 0 to 50% of 20% MeOH/DCM (w/ 1% NH_4OH) in DCM. Product fractions were concentrated under reduced pressure to dryness to afford **21** (bis(3-pentyloctyl) 9-((tetrahydro-2H-pyran-4-yl)amino)heptadecanedioate**,** 28.0 mg, 30.0%) as a colorless oil. ^1H NMR (500 MHz, Methanol-*d*4) δ ppm 0.9 (t, *J*=7.1 Hz, 12 H) 1.3 - 1.5 (m, 56 H) 1.6 (br d, J=6.6 Hz, 8 H) 1.8 (br dd, *J*=12.7, 2.0 Hz, 2 H) 2.3 (t, *J*=7.3 Hz, 4 H) 2.7 - 2.9 (m, 2 H) 3.4 (td, *J*=11.8, 1.8 Hz, 2 H) 3.9 (br dd, *J*=12.0, 2.5 Hz, 2 H) 4.1 (t, *J*=6.7 Hz, 4 H); $C_{48}H_{93}NO_5$ *m/z* calcd. 763.705 observed 764.8 $[M+H]^+$ (LCMS).

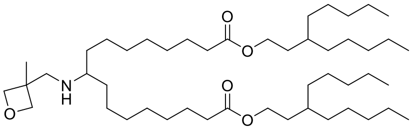

22

Lipid 22, Step d) Sodium triacetoxyhydroborate (44.7 mg, 0.21 mmol) was added in one portion to a stirred solution of (3-methyloxetan-3-yl)methanamine (0.019 mL, 0.19 mmol), acetic acid (0.012 mL, 0.21 mmol) and bis(3-pentyloctyl) 9-oxoheptadecanedioate (47.7 mg, 0.07 mmol) in 1,2-DCE (2 mL) and NMP (0.5 mL) at 25°C under argon. The resulting solution was stirred at 25 °C for 30 hours. The reaction mixture was diluted with DCM (15 mL) and water (5 mL) with sat. aq. Na_2CO_3 (10 mL). The layers were separated, and the aqueous layer was extracted with DCM (3 x 15 mL). The combined organic layers were dried over $MgSO_4$, filtered and concentrated under reduced pressure to dryness to afford crude product. The resulting residue was purified 2 times by flash silica chromatography, elution gradient 0 to 25% of 20% MeOH/DCM (w/ 1% NH_4OH) in DCM. Product fractions were concentrated under reduced pressure to dryness to afford **22** (bis(3-pentyloctyl) 9-(((3-methyloxetan-3-yl)methyl)amino)heptadecanedioate, 21.2 mg, 39.5%) as a colorless oil. ^1H NMR (500 MHz, Methanol-*d*4) δ ppm 0.9 (t, *J*=7.1 Hz, 12 H) 1.3 (br s, 51 H) 1.4 - 1.5 (m, 6 H) 1.6 - 1.6 (m, 8 H) 2.3 (s, 4 H) 2.5 - 2.5 (m, 1 H) 2.8 - 2.8 (m, 2 H) 4.1 - 4.1 (m, 4 H) 4.4 (s, 2 H) 4.4 - 4.5 (m, 2 H); $C_{48}H_{93}NO_5$ *m/z* calcd. 763.705 observed 764.6 $[M+H]^+$ (LCMS).

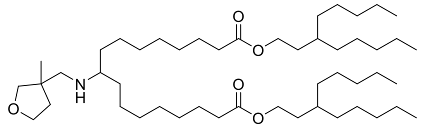

23

Lipid 23, Step d) Sodium triacetoxyhydroborate (38.2 mg, 0.18 mmol) was added in one portion to a stirred solution of (3-methyltetrahydrofuran-3-yl)methanamine (0.194 mL, 0.17 mmol), acetic acid (0.011 mL, 0.19 mmol) and bis(3-pentyloctyl) 9-oxoheptadecanedioate (43.7 mg, 0.06 mmol) in 1,2-DCE (2 mL) and NMP (0.5 mL) at 25°C under argon. The resulting solution was stirred at 25 °C for 30 hours. The reaction mixture was diluted with DCM (15 mL) and water (5 mL) with sat. aq. Na_2CO_3 (10 mL). The layers were separated, and the aqueous layer was extracted with DCM (3 x 15 mL). The combined organic layers were dried over $MgSO_4$, filtered and concentrated under reduced pressure to dryness to afford crude product. The resulting residue was purified by flash silica chromatography, elution gradient 0 to 35% of 20% MeOH/DCM (w/ 1% NH_4OH) in DCM. Product fractions were concentrated under reduced pressure to dryness to afford **23** (bis(3-pentyloctyl)9-(((3-methyltetrahydrofuran-3-l)methyl)amino)heptadecanedioate, 24.3 mg, 48.5%) as a colorless oil. ^{1}H NMR (500 MHz, Methanol-*d*4) δ ppm 0.9 (t, *J*=6.9 Hz, 12 H) 1.1 (s, 3 H) 1.3 (br s, 48 H) 1.4 (br s, 6 H) 1.5 - 1.7 (m, 9 H) 1.8 - 1.9 (m, 1 H) 2.3 (t, *J*=7.2 Hz, 4 H) 2.4 - 2.5 (m, 1 H) 2.6 (s, 2 H) 3.4 - 3.4 (m, 1 H) 3.6 - 3.6 (m, 1 H) 3.8 - 3.9 (m, 2 H) 4.1 (s, 4 H); $C_{49}H_{95}NO_5$ *m/z* calcd. 777.721 observed 778.9 $[M+H]^+$ (LCMS).

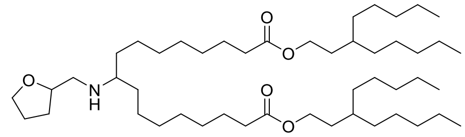

24

Lipid 24, Step d) Sodium triacetoxyhydroborate (43.0 mg, 0.20 mmol) was added in one portion to a stirred solution of (tetrahydrofuran-2-yl)methanamine (0.020 mL, 0.20 mmol), acetic acid (0.012 mL, 0.22 mmol) and bis(3-pentyloctyl) 9-oxoheptadecanedioate (49.2 mg, 0.07 mmol) in 1,2-DCE (2 mL) and NMP (0.5 mL) at 25°C under argon. The resulting solution was stirred at 25 °C for 30 hours. The reaction mixture was diluted with DCM (15 mL) and water (5 mL) with sat. aq. Na_2CO_3 (10 mL). The layers were separated, and the aqueous layer was extracted with DCM (3 x 15 mL). The combined organic layers were dried over $MgSO_4$, filtered and concentrated under reduced pressure to dryness to afford crude product. The resulting residue was purified by flash silica chromatography, elution gradient 0 to 35% of 20% MeOH/DCM (w/ 1% NH_4OH) in DCM. Product fractions were concentrated under reduced pressure to dryness to afford **24** (bis(3-pentyloctyl) 9-(((tetrahydrofuran-2-yl)methyl)amino)heptadecanedioate, 22.6 mg, 40.8%) as a colorless oil. ^{1}H NMR (500 MHz, Methanol-*d*4) δ ppm 0.9 (t, *J*=6.9 Hz, 12 H) 1.2 - 1.4 (m, 49 H) 1.4 - 1.5 (m, 6 H) 1.5 - 1.7 (m, 9 H) 1.9 - 2.0 (m, 2 H) 2.0 - 2.1 (m, 1 H) 2.3 (s, 4 H) 2.5 - 2.6 (m, 2 H) 2.7 - 2.8 (m, 1 H) 3.8 (br d, *J*=7.3 Hz, 1 H) 3.8 (br d, *J*=7.6 Hz, 1 H) 4.0 (br d, *J*=5.6 Hz, 1 H) 4.1 - 4.1 (m, 4 H); $C_{48}H_{93}NO_5$ *m/z* calcd. 763.705 observed 764.9 $[M+H]^+$ (LCMS).

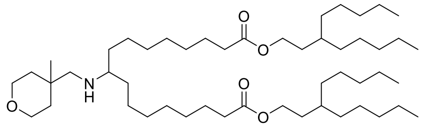

25

Lipid 25, Step d) Sodium triacetoxyhydroborate (38.1 mg, 0.18 mmol) was added in one portion to a stirred solution of (4-methyltetrahydro-2H-pyran-4-yl)methanamine (0.217 mL, 0.17 mmol), acetic acid (0.011 mL, 0.19 mmol) and bis(3-pentyloctyl) 9-oxoheptadecanedioate (43.6 mg, 0.06 mmol) in 1,2-DCE (2 mL) and NMP (0.5 mL) at 25°C under argon. The resulting solution was stirred at 25 °C for 30 hours. The reaction mixture was diluted with DCM (15 mL) and water (5 mL) with sat. aq. Na_2CO_3 (10 mL). The layers were separated, and the aqueous layer was extracted with DCM (3 x 15 mL). The combined organic layers were dried over $MgSO_4$, filtered and concentrated under reduced pressure to dryness to afford crude product. The resulting residue was purified by flash silica chromatography, elution gradient 0 to 25% of 20% MeOH/DCM (w/ 1% NH_4OH) in DCM. Product fractions were concentrated under reduced pressure to dryness to afford **25** (bis(3-pentyloctyl) 9-(((4-methyltetrahydro-2H-pyran-4-yl)methyl)amino)heptadecanedioate, 13.9 mg, 27.3%) as a colorless dry film. ^{1}H NMR (400 MHz, Methanol-*d*4) δ ppm 0.9 (t, J=7.0 Hz, 12 H) 1.0 (s, 3 H) 1.3 (br d, *J*=16.6 Hz, 50 H) 1.4 (br d, J=4.3 Hz, 6 H) 1.6 (br d, *J*=6.5 Hz, 10 H) 2.3 (s, 4 H) 2.4 - 2.5 (m, 3 H) 3.7 (s, 4 H) 4.1 - 4.2 (m, 4 H); $C_{50}H_{97}NO_5$ *m/z* calcd. 791.737 observed 792.8 $[M+H]^+$ (LCMS).

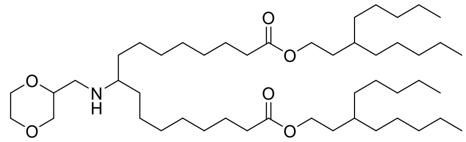

26

Lipid 26, Step d) Sodium triacetoxyhydroborate (36.6 mg, 0.17 mmol) was added in one portion to a stirred solution of (1,4-dioxan-2-yl)methanamine (0.017 mL, 0.16 mmol), acetic acid (9.88 µl, 0.17 mmol) and bis(3-pentyloctyl) 9-oxoheptadecanedioate (39.1 mg, 0.06 mmol) in 1,2-DCE (2 mL) and NMP (0.5 mL) at 25°C under argon. The resulting solution was stirred at 25 °C for 30 hours. The reaction mixture was diluted with DCM (15 mL) and water (5 mL) with sat. aq. Na_2CO_3 (10 mL). The layers were separated, and the aqueous layer was extracted with DCM (3 x 15 mL). The combined organic layers were dried over $MgSO_4$, filtered and concentrated under reduced pressure to dryness to afford crude product. The resulting residue was purified by flash silica chromatography, elution gradient 0 to 35% of 20% MeOH/DCM (w/ 1% NH_4OH) in DCM. Product fractions were concentrated under reduced pressure to dryness to afford **26** (bis(3-pentyloctyl) 9-(((1,4-dioxan-2-yl)methyl)amino)heptadecanedioate, 23.10 mg, 51.4%) as a colorless oil. ^{1}H NMR (500 MHz, Methanol-*d*4) δ ppm 0.9 (t, *J*=7.1 Hz, 12 H) 1.3 - 1.4 (m, 48 H) 1.4 - 1.5 (m, 6 H) 1.6 - 1.6 (m, 8 H) 2.3 (t, *J*=7.3 Hz, 4 H) 2.5 - 2.6 (m, 2 H) 2.6 (s, 3 H) 3.3 - 3.3 (m, 1 H) 3.6 (s, 1 H) 3.6 - 3.8 (m, 4 H) 3.8 - 3.8 (m, 1 H) 4.1 (s, 4 H); $C_{48}H_{93}NO_6$ *m/z* calcd. 779.700 observed 780.9 $[M+H]^+$ (LCMS).

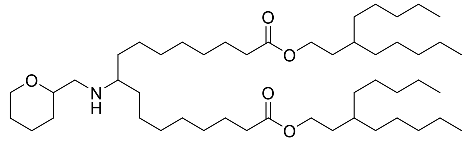

27

Lipid 27, Step d) Sodium triacetoxyhydroborate (32.1 mg, 0.15 mmol) was added in one portion to a stirred solution of (tetrahydro-2H-pyran-2-yl)methanaminium chloride (20.07 mg, 0.13 mmol) and bis(3-pentyloctyl) 9-oxoheptadecanedioate (42.8 mg, 0.06 mmol) in 1,2-DCE (2 mL) and NMP (0.5 mL) at 25°C under argon. The resulting solution was stirred at 25 °C for 30 hours. The reaction mixture was diluted with DCM (15 mL) and water (5 mL) with sat. aq. Na_2CO_3 (10 mL). The layers were separated, and the aqueous layer was extracted with DCM (3 x 15 mL). The combined organic layers were dried over $MgSO_4$, filtered and concentrated under reduced pressure to dryness to afford crude product. The resulting residue was purified by flash silica chromatography, elution gradient 0 to 30% of 20% MeOH/DCM (w/ 1% NH_4OH) in DCM. Product fractions were concentrated under reduced pressure to dryness to afford **27** (bis(3-pentyloctyl) 9-(((tetrahydro-2H-pyran-2-yl)methyl)amino)heptadecanedioate, 17.40 mg, 35.5%) as a colorless oil. ^{1}H NMR (500 MHz, Methanol-*d*4) δ ppm 0.9 (t, *J*=6.9 Hz, 12 H) 1.2 - 1.4 (m, 51 H) 1.4 - 1.5 (m, 6 H) 1.5 - 1.6 (m, 12 H) 1.9 (br s, 1 H) 2.3 (t, *J*=7.3 Hz, 4 H) 2.5 - 2.6 (m, 2 H) 2.7 - 2.7 (m, 1 H) 3.5 (br t, *J*=9.4 Hz, 2 H) 4.0 (br d, *J*=11.1 Hz, 1 H) 4.1 - 4.1 (m, 4 H); $C_{49}H_{95}NO_5$ *m/z* calcd. 777.721 observed 778.9 $[M+H]^+$ (LCMS).

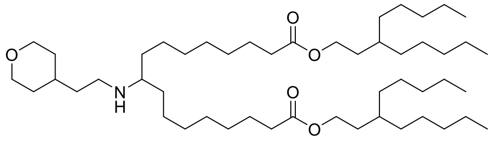

28

Lipid 28, Step d) Sodium triacetoxyhydroborate (43.7 mg, 0.21 mmol) was added in one portion to a stirred solution of 2-(tetrahydro-2H-pyran-4-yl)ethan-1-amine (0.026 mL, 0.19 mmol), acetic acid (0.012 mL, 0.21 mmol) and bis(3-pentyloctyl) 9-oxoheptadecanedioate (46.7 mg, 0.07 mmol) in 1,2-DCE (2 mL) and NMP (0.5 mL) at 25°C under argon. The resulting solution was stirred at 25 °C for 30 hours. The reaction mixture was diluted with DCM (15 mL) and water (5 mL) with sat. aq. Na_2CO_3 (10 mL). The layers were separated, and the aqueous layer was extracted with DCM (3 x 15 mL). The combined organic layers were dried over $MgSO_4$, filtered and concentrated under reduced pressure to dryness to afford crude product. The resulting residue was purified by flash silica chromatography, elution gradient 0 to 30% of 20% MeOH/DCM (w/ 1% NH_4OH) in DCM. Product fractions were concentrated under reduced pressure to dryness to afford **28** (bis(3-pentyloctyl) 9-((2-(tetrahydro-2H-pyran-4-yl)ethyl)amino)heptadecanedioate, 23.2 mg, 43%) as a colorless oil. ^{1}H NMR (400 MHz, Methanol-*d*4) δ ppm 0.9 (t, *J*=7.0 Hz, 12 H) 1.3 (br s, 50 H) 1.4 - 1.5 (m, 8 H) 1.6 (br d, *J*=6.6 Hz, 11 H) 2.3 (s, 4 H) 2.5 - 2.6 (m, 1 H) 2.6 - 2.7 (m, 2 H) 3.4 - 3.5 (m, 2 H) 3.9 - 4.0 (m, 2 H) 4.1 - 4.1 (m, 4 H); $C_{50}H_{97}NO_5$ *m/z* calcd. 791.737 observed 792.8 $[M+H]^+$ (LCMS).

Lipid 2 Synthesis:

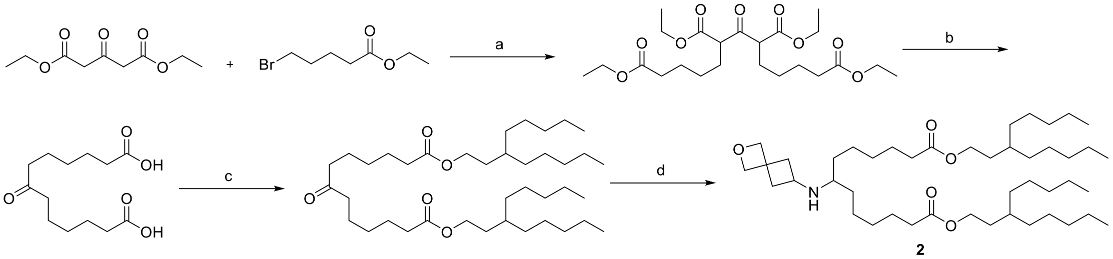

Reactants/Reagents: a) NaOEt, EtOH; b) conc. HCl, AcOH; c) 3-pentyloctan-1-ol, EDC.HCl, DIPEA, DMAP, DCM; d) 2-oxaspiro[3.3]heptan-6-aminium chloride, $NaBH(OAc)_3$, 1,2-DCE: NMP (4:1)

a) Sodium ethanolate (1.111 g, 16.32 mmol) was added portion-wise to a stirred solution of diethyl 3-oxopentanedioate (1.977 mL, 10.88 mmol) in ethanol (absolute, 99.5%) (10 mL) at 81°C under argon. The resulting solution was stirred at 81 °C for 1 hour. To it was added ethyl 5-bromopentanoate (3.87 mL, 24.48 mmol) dropwise and stirred at 81 °C for 18 hours. The reaction mixture was cooled and the solvent was evaporated. The reaction mixture was diluted with DCM (25 mL) and washed with sat. aq. NaCl (25 mL). The organic layer was dried over $MgSO_4$, filtered and concentrated under reduced pressure to dryness to afford crude product. The resulting residue was purified by flash silica chromatography, elution gradient 0 to 60% EtOAc in hexanes. Product fractions were concentrated under reduced pressure to afford **tetraethyl 6-oxoundecane-1,5,7,11-tetracarboxylate** (3.28 g, 66%) as a pale yellow liquid. ^1H NMR (500 MHz, Chloroform-*d*) δ ppm 1.2 - 1.4 (m, 16 H) 1.6 - 1.7 (m, 4 H) 1.8 - 2.0 (m, 4 H) 2.3 - 2.4 (m, 4 H) 4.1 - 4.3 (m, 10 H).

b) Hydrogen chloride (17.65 mL, 214.99 mmol) was added slowly to a stirred solution of tetraethyl 6-oxoundecane-1,5,7,11-tetracarboxylate (3.821 g, 8.33 mmol) in acetic acid (8.5 mL) at 25°C. The resulting solution was stirred at 102 °C for 18 hours w/ reflux condenser and a vent to remove excess of HCl gas. Reaction was cooled to RT and poured the reaction mixture on ice-water (50 mL) and was allowed to sit for 30 min. The precipitate was collected by filtration, washed with cold water (3 x 20mL) and dried under vacuum to afford crude material as a yellow solid. The crude product was purified by crystallization from acetone to afford **7-oxotridecanedioic acid** (0.283 g, 13%) as a white powder. ^1H NMR (500 MHz, DMSO-*d*6) δ ppm 1.2 - 1.2 (m, 4 H) 1.4 (m, 8 H) 2.2 (t, *J*=7.3 Hz, 4 H) 2.4 (t, J=7.3 Hz, 4 H); $C_{13}H_{22}O_5$ *m/z* calcd. 258.147 observed 257.1 $[M-H]^-$ (LCMS).

c) 3-(((ethylimino)methylene)amino)-N,N-dimethylpropan-1-amine hydrochloride (204 mg, 1.06 mmol) was added in one portion to a stirred solution of 7-oxotridecanedioic acid (101.8 mg, 0.39 mmol), 3-pentyloctan-1-ol (197 mg, 0.99 mmol), N-ethyl-N-isopropylpropan-2-amine (275 µL, 1.58 mmol) and N,N-dimethylpyridin-4-amine (9.63 mg, 0.08 mmol) in DCM (7 mL) at 0°C under argon. The resulting solution was stirred at 25 °C for 24 hours. The reaction mixture was quenched with sat aq. NH_4Cl (15 mL), extracted with EtOAc (3 x 20 mL), the organic layer was dried over $MgSO_4$, filtered and concentrated under reduced pressure to dryness to afford colorless oil. The resulting residue was purified by flash silica chromatography, elution gradient 0 to 40% EtOAc in hexanes. Product fractions were concentrated under reduced pressure to dryness to afford **bis(3-pentyloctyl) 7-oxotridecanedioate** (0.227 g, 92%) as a

colorless oil. ^{1}H NMR (500 MHz, Chloroform-*d*) δ ppm 0.9 (t, *J*=7.1 Hz, 12 H) 1.2 - 1.4 (m, 36 H) 1.4 (br d, *J*=5.0 Hz, 2 H) 1.5 - 1.7 (m, 12 H) 2.3 (t, *J*=7.5 Hz, 4 H) 2.4 (t, *J*=7.5 Hz, 4 H) 4.1 (t, *J*=7.1 Hz, 4 H).

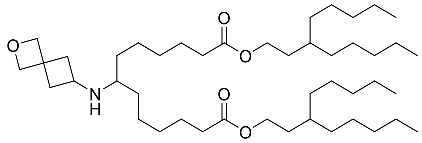

2

d) Sodium triacetoxyhydroborate (44.2 mg, 0.21 mmol) was added in one portion to a stirred solution of bis(3-pentyloctyl) 7-oxotridecanedioate (52 mg, 0.08 mmol), and 2-oxaspiro[3.3]heptan-6-aminium chloride (30.0 mg, 0.20 mmol) in NMP (0.500 mL) and 1,2-DCE (2 mL) at 25°C under argon. The resulting suspension was stirred at 25 °C for 40 hours. The reaction mixture was diluted with DCM (15 mL), water (5 mL) and sat. aq. Na_2CO_3 (10 mL). The layers were separated, and the aqueous layer was extracted with DCM (3 x 15 mL). The combined organic layers were dried over $MgSO_4$, filtered and concentrated under reduced pressure to dryness to afford crude product. The resulting residue was purified by flash silica chromatography, elution gradient 0 to 50% of 20%MeOH/DCM (w/ 1% NH_4OH) in DCM. Product fractions were concentrated under reduced pressure to dryness to afford **2** (bis(3-pentyloctyl) 7-((2-oxaspiro[3.3]heptan-6-yl)amino)tridecanedioate, 0.025 g, 42%) as a colorless oil. ^{1}H NMR (500 MHz, Methanol-*d4*) δ ppm 0.9 - 1.0 (m, 12 H) 1.3 - 1.4 (m, 44 H) 1.4 - 1.5 (m, 2 H) 1.5 - 1.7 (m, 8 H) 1.9 - 2.0 (m, 2 H) 2.3 (t, *J*=7.3 Hz, 4 H) 2.4 - 2.6 (m, 3 H) 3.2 (t, *J*=7.7 Hz, 1 H) 4.1 (t, *J*=6.8 Hz, 4 H) 4.6 (s, 2 H) 4.7 (s, 2 H); $C_{48}H_{93}NO_5$ *m/z* calcd. 719.643 observed 720.7 $[M+H]^+$ (LCMS).

Lipid 3 Synthesis:

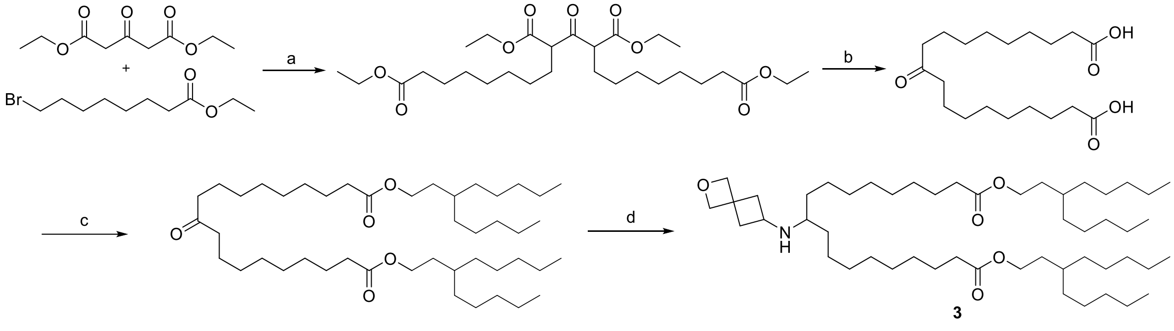

Reagents: a) NaOEt, EtOH; b) conc. HCl, AcOH; c) 3-pentyloctan-1-ol, EDC.HCl, DIPEA, DMAP, DCM; d) 2-oxaspiro[3.3]heptan-6-aminium chloride, $NaBH(OAc)_3$, 1,2-DCE: NMP (4:1)

a) Sodium ethanolate (2.52 g, 37.09 mmol) was added portion-wise to a stirred solution of diethyl 3-oxopentanedioate (4.49 mL, 24.73 mmol) in ethanol (absolute, 99.5%) (15 mL) at 25 C over a period of 10 minutes under argon. The resulting suspension was stirred at 81 °C for 1 hour. To the reaction mixture ethyl 8-bromooctanoate (12.53 ml, 59.35 mmol) was added dropwise and the suspension was stirred at 81 °C for a further 18 hours. The reaction mixture was cooled to RT and concentrated under reduced pressure to dryness and redissolved in DCM (50 mL), and washed sequentially with water (50 mL), sat. aq. sodium chloride (50 mL). The organic layer was dried over $MgSO_4$, filtered and concentrated under reduced pressure to afford crude product. The resulting residue was purified by flash silica

chromatography, elution gradient 0 to 50% EtOAc in hexanes. Product fractions were concentrated under reduced pressure to dryness to afford **tetraethyl 9-oxoheptadecane-1,8,10,17-tetracarboxylate** (8.80 g, 66%) as a pale yellow oil. ^{1}H NMR (500 MHz, Chloroform-*d*) δ ppm 1.2 - 1.4 (m, 28 H) 1.6 - 1.7 (m, 4 H) 1.7 - 1.9 (m, 4 H) 2.2 - 2.3 (m, 4 H) 4.1 - 4.2 (m, 10 H).

b) Hydrogen chloride (34.4 mL, 418.34 mmol) was added slowly to a stirred solution of tetraethyl tetraethyl 9-oxoheptadecane-1,8,10,17-tetracarboxylate (8.8 g, 16.21 mmol) in acetic acid (21 mL) at 25°C. The resulting solution was stirred at 102 °C for 18 hours w/ reflux condenser and a vent to remove excess HCl gas. Reaction was cooled to RT and poured on ice-water (40 mL) and was allowed to sit for 30 min. The precipitate was collected by filtration, washed with cold water (3 x 20 mL) and dried under vacuum to afford crude product as a pale yellow solid. The crude product was purified by crystallization from acetone to afford **10-oxononadecanedioic acid** (1.96 g, 35%). ^{1}H NMR (500 MHz, DMSO-*d*6) δ ppm 1.2 (br s, 16 H) 1.4 - 1.5 (m, 8 H) 2.2 (t, *J*=7.3 Hz, 4 H) 2.3 - 2.4 (m, 4 H) 12.0 (br s, 2 H); $C_{19}H_{34}O_5$ *m/z* calcd. 342.241 observed 341.2 $[M-H]^-$ (LCMS).

c) 3-(((ethylimino)methylene)amino)-N,N-dimethylpropan-1-amine hydrochloride (272 mg, 1.42 mmol) was added in one portion to a stirred solution of 10-oxononadecanedioic acid (180 mg, 0.53 mmol), N,N-dimethylpyridin-4-amine (9.63 mg, 0.08 mmol), 3-pentyloctan-1-ol (253 mg, 1.26 mmol) and N-ethyl-N-isopropylpropan-2-amine (330 µl, 1.89 mmol) in DCM (8 mL) at 0°C under argon. The resulting solution was stirred at 25 °C for 18 hours. The reaction mixture was diluted with DCM (15 mL) and water (15 mL). The layers were separated, and the aqueous layer was extracted with DCM (3 x 20 mL). The combined organic layers were washed with 0.5 M citric acid (15 mL) and sat**.** aq**.** NaCl (15 mL). The organic layer was dried over $MgSO_4$, filtered and concentrated under reduced pressure to dryness to afford crude product. The resulting residue was purified by flash silica chromatography, elution gradient 0 to 30% hexanes in EtOAc. Product fractions were concentrated under reduced pressure to dryness to afford **bis(3-pentyloctyl) 10-oxononadecanedioate** (0.343 g, 92%) as a colorless oil. ^{1}H NMR (500 MHz, Chloroform-*d*) δ ppm 0.9 (t, *J*=7.1 Hz, 12 H) 1.2 - 1.4 (m, 48 H) 1.4 (br s, 2 H) 1.5 - 1.6 (m, 12 H) 2.3 (t, *J*=7.5 Hz, 4 H) 2.3 - 2.4 (m, 4 H) 4.0 - 4.1 (m, 4 H).

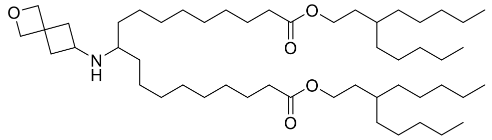

3

d) Sodium triacetoxyhydroborate (54.4 mg, 0.26 mmol) was added in one portion to a stirred solution of bis(3-pentyloctyl) 10-oxononadecanedioate (75.7 mg, 0.11 mmol) and 2-oxaspiro[3.3]heptan-6-aminium chloride (33.6 mg, 0.22 mmol) in 1,2-DCE (2.4 mL) and NMP (0.6 mL) at 25°C under argon. The resulting suspension was stirred at 25 °C for 40 hours. The reaction mixture was diluted with DCM (10 mL), water (5 mL) and sat. aq. Na_2CO_3 (5 mL). The layers were separated, and the aqueous layer was extracted with DCM (3 x 10 mL). The combined organic layer was dried over $MgSO_4$, filtered and concentrated under reduced pressure to dryness to afford crude product. The resulting residue was purified by flash silica chromatography, elution gradient 0 to 35% of 20% MeOH/DCM (w/ 1% NH_4OH) in DCM. Product fractions were concentrated under reduced pressure to dryness to afford **3** (bis(3-pentyloctyl) 10-((2-oxaspiro[3.3]heptan-6-yl)amino)nonadecanedioate, 0.033 g, 38.0%) as a colorless oil. ^{1}H NMR (500 MHz,

Methanol-*d*4) δ ppm 0.9 (t, *J*=7.0 Hz, 12 H) 1.2 - 1.4 (m, 56 H) 1.4 - 1.5 (m, 2 H) 1.5 - 1.6 (m, 8 H) 2.0 (td, *J*=9.1, 2.8 Hz, 2 H) 2.3 (t, *J*=7.3 Hz, 4 H) 2.4 - 2.6 (m, 3 H) 3.2 (br t, *J*=7.8 Hz, 1 H) 4.1 (t, *J*=6.7 Hz, 4 H) 4.6 (s, 2 H) 4.7 (s, 1 H); $C_{51}H_{97}NO_5$ *m/z* calcd. 803.737 observed 804.8 $[M+H]^+$ (LCMS).

Lipid 4 Synthesis:

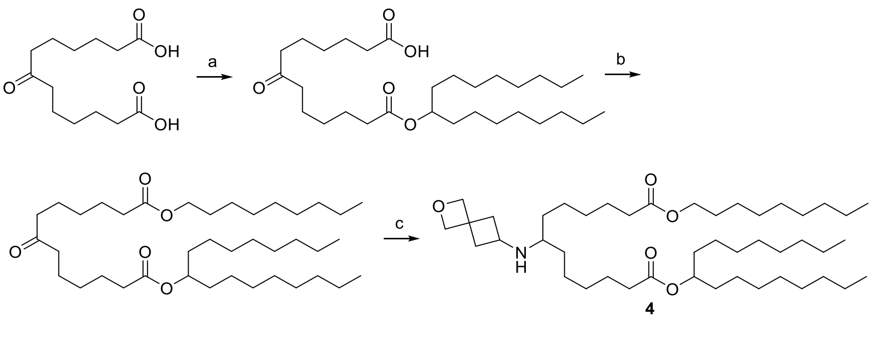

Reagents: a) heptadecan-9-ol, EDC.HCl, DIPEA, DMAP, DCM; b) nonan-1-ol, EDC.HCl, DIPEA, DMAP, DCM; c) 2-oxaspiro[3.3]heptan-6-aminium chloride, $NaBH(OAc)_3$, 1,2-DCE: NMP (4:1)

a) 3-(((ethylimino)methylene)amino)-N,N-dimethylpropan-1-amine hydrochloride (202 mg, 1.05 mmol) was added in one portion to a stirred solution of 7-oxotridecanedioic acid (181.4 mg, 0.70 mmol), N-ethyl-N-isopropylpropan-2-amine (367 µL, 2.11 mmol), heptadecan-9-ol (120 mg, 0.47 mmol) and N,N-dimethylpyridin-4-amine (18.02 mg, 0.15 mmol) in DCM (9 mL) at 0°C under argon. The resulting solution was stirred at 25 °C for 18 hours. The reaction mixture was concentrated under reduced pressure to dryness and redissolved in EtOAc (15 mL), water (10 mL) and 5% citric acid solution (5 mL). The layers were separated, and the aqueous layer was extracted with EtOAc (3 x 20 mL). The combined organic layers were dried over $MgSO_4$, filtered and concentrated under reduced pressure to dryness to afford crude product. The resulting residue was purified by flash silica chromatography, elution gradient 0 to 40% EtOAc in hexanes. Product fractions were concentrated under reduced pressure to dryness to afford **13-(heptadecan-9-yloxy)-7,13-dioxotridecanoic acid** (0.102 g, 29.2%) as a colorless oil. ^1H NMR (500 MHz, Chloroform-*d*) δ ppm 0.9 (t, *J*=6.9 Hz, 6 H) 1.2 - 1.3 (m, 21 H) 1.3 - 1.4 (m, 7 H) 1.5 - 1.6 (m, 4 H) 1.6 - 1.7 (m, 8 H) 2.3 (t, *J*=7.5 Hz, 2 H) 2.3 - 2.5 (m, 6 H) 4.8 - 4.9 (m, 1 H); $C_{30}H_{56}O_5$ m/z calcd. 496.413 observed 495.5 [M-H]- (LCMS).

b) 3-(((ethylimino)methylene)amino)-N,N-dimethylpropan-1-amine hydrochloride (83 mg, 0.43 mmol) was added in one portion to a stirred solution of 13-(heptadecan-9-yloxy)-7,13-dioxotridecanoic acid (102 mg, 0.21 mmol), nonan-1-ol (64.2 µL, 0.37 mmol), N,N-dimethylpyridin-4-amine (5.02 mg, 0.04 mmol) and N-ethyl-N-isopropylpropan-2-amine (118 µL, 0.68 mmol) in DCM (6 mL) at 0°C under nitrogen. The resulting solution was allowed to come to RT and stirred at 25 °C for 30 hours. The reaction mixture was diluted with DCM (15 mL) and water (15 mL). The layers were separated, and the aqueous layer was extracted with DCM (3 x 15 mL). The combined organic layers were washed with 5% citric acid solution (10 mL). The organic layer was dried over $MgSO_4$, filtered and concentrated under reduced pressure to dryness to afford crude product. The resulting residue was purified by flash silica chromatography, elution gradient 0 to 40% EtOAc in hexanes. Product fractions were concentrated under reduced pressure to dryness to afford **1-(heptadecan-9-yl) 13-nonyl 7-oxotridecanedioate** (0.085 g, 66.5%) as a colorless oil. ^1H NMR (500 MHz, Chloroform-*d*) δ ppm 0.9 (t, J=6.9 Hz, 9 H) 1.2 - 1.4 (m, 40 H) 1.5 - 1.6 (m, 4 H) 1.6 - 1.7 (m, 10 H) 2.3 (q, J=7.1 Hz, 4 H) 2.4 (t, J=7.4 Hz, 4 H) 4.1 (t, J=6.7 Hz, 2 H) 4.8 - 4.9 (m, 1 H).

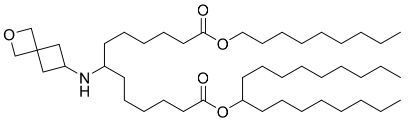

4

c) Sodium triacetoxyhydroborate (37.6 mg, 0.18 mmol) was added in one portion to a stirred suspension of 1-(heptadecan-9-yl) 13-nonyl 7-oxotridecanedioate (40.9 mg, 0.07 mmol), and 2-oxaspiro[3.3]heptan-6-aminium chloride (23.57 mg, 0.16 mmol) in 1,2-DCE (2 mL) and NMP (0.5 mL) under nitrogen. The resulting suspension was stirred at 25 °C for 40 hours. The reaction mixture was diluted with DCM (15 mL), water (5 mL) and sat. aq. Na_2CO_3 (10 mL). The layers were separated, and the aqueous layer was extracted with DCM (3 x 15 mL). The combined organic layers were dried over $MgSO_4$, filtered and concentrated under reduced pressure to dryness to afford crude product. The resulting residue was purified by flash silica chromatography, elution gradient 0 to 50% of 20% MeOH/DCM (w/ 1% NH_4OH) in DCM. Product fractions were concentrated under reduced pressure to dryness to afford **4** (1-(heptadecan-9-yl) 13-nonyl 7-((2-oxaspiro[3.3]heptan-6-yl)amino)tridecanedioate, 0.023 g, 49.3%) as a colorless oil. ^{1}H NMR (500 MHz, Methanol-*d4*) δ ppm 0.9 (t, *J*=6.6 Hz, 9 H) 1.3 - 1.4 (m, 48 H) 1.5 - 1.6 (m, 4 H) 1.6 - 1.7 (m, 6 H) 1.9 - 2.0 (m, 2 H) 2.3 (br t, *J*=7.2 Hz, 4 H) 2.4 - 2.5 (m, 1 H) 2.5 - 2.6 (m, 2 H) 3.2 (t, *J*=7.9 Hz, 1 H) 4.1 (t, *J*=6.6 Hz, 2 H) 4.6 (s, 2 H) 4.7 (s, 2 H) 4.9 - 4.9 (m, 1 H); $C_{45}H_{85}NO_5$ *m/z* calcd. 719.643 observed 720.8 $[M+H]^+$ (LCMS).

Lipid 5 Synthesis

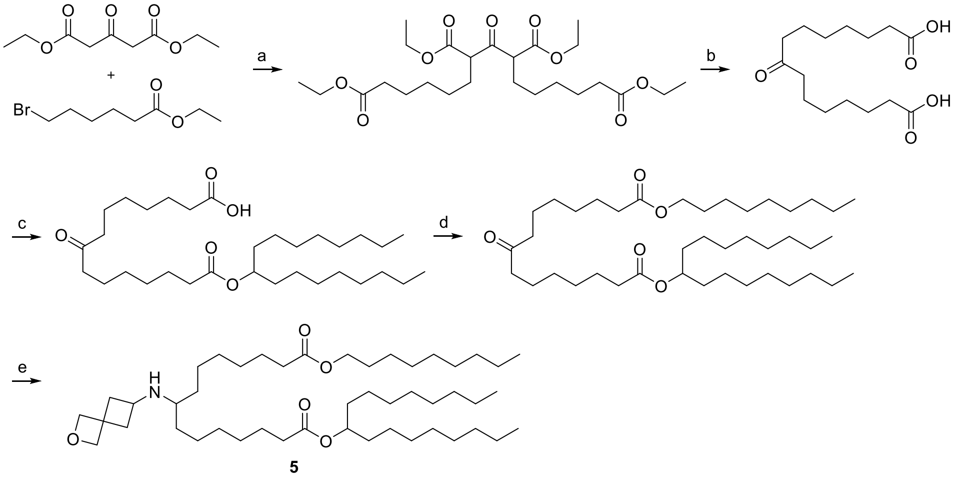

Reactants/Reagents: a) NaOEt, EtOH; b) conc. HCl, AcOH; c) heptadecan-9-ol, EDC.HCl, DIPEA, DMAP, DCM; d) nonan-1-ol, EDC.HCl, DIPEA, DMAP, DCM; e) 2-oxaspiro[3.3]heptan-6-aminium chloride, $NaBH(OAc)_3$, 1,2-DCE: NMP (4:1)

a) Sodium ethanolate (3.37 g, 49.45 mmol) was added in one portion to stirred diethyl 3-oxopentanedioate (4.49 mL, 24.73 mmol) in EtOH (100 mL) under argon. The resulting mixture was stirred at 80 °C for 1 hour followed by addition of ethyl 6-bromohexanoate (15.51 mL, 86.55 mmol) dropwise. The reaction was refluxed overnight. The reaction mixture was concentrated and diluted with EtOAc (200 mL) and washed with water (200 mL x 2). The organic layer was dried over Na_2SO_4, filtered and evaporated to afford crude product. The crude product was purified by flash silica chromatography, elution gradient 0 to 20% EtOAc in hexanes. Pure fractions were evaporated to dryness to afford **tetraethyl 7-**

oxotridecane-1,6,8,13-tetracarboxylate (7.51 g, 62.4%) as an orange oil. ^{1}H NMR (500 MHz, Chloroform-*d*) 1.20 - 1.41 (20H, m), 1.56 - 1.69 (4H, m), 1.78 - 1.94 (4H, m), 2.22 - 2.35 (4H, m), 3.55 - 3.76 (2H, m), 4.06 - 4.27 (8H, m).

b) Tetraethyl 7-oxotridecane-1,6,8,13-tetracarboxylate (7.5 g, 15.41 mmol) was added in one portion to concentrated hydrochloric acid (50 mL, 15.41 mmol) and acetic acid (25 mL) at 25°C under nitrogen. The resulting mixture was stirred at 100 °C for 15 hours. The solution was cooled to room temperature. The reaction mixture was poured into water and the precipitate was collected by filtration. The crude product was purified by crystallization from acetone to afford **8-oxopentadecanedioic acid** (1.482 g, 33.6%) as a white solid. ^{1}H NMR (500 MHz, DMSO-*d*6) 1.21 (8H, br s), 1.35 - 1.55 (8H, m), 2.17 (4H, t), 2.37 (4H, t), 11.97 (2H, s).

c) 3-(((ethylimino)methylene)amino)-N,N-dimethylpropan-1-amine hydrochloride (0.201 g, 1.05 mmol) was added in one portion to a stirred solution of 9-heptadecanol (0.143 g, 0.56 mmol)),8-oxopentadecanedioic acid (0.200 g, 0.70 mmol), N-ethyl-N-isopropylpropan-2-amine (0.378 mL, 2.17 mmol), and N,N-dimethylpyridin-4-amine (0.017 g, 0.14 mmol) in DCM (5 mL) at 0°C under argon. The resulting solution was stirred at 25 °C for 16 hours. The reaction mixture was concentrated under reduced pressure to dryness and redissolved in EtOAc (20 mL) and sat. aq. $NaHCO_3$ (20 mL). The layers were separated, and the aqueous layer was extracted with EtOAc (3 x 30 mL). The combined organic layers were washed sequentially with 5% citric acid (25 mL) and sat. aq. NaCl (25 mL). The organic layer was dried over $MgSO_4$, filtered and concentrated under reduced pressure to dryness to afford crude product. The resulting residue was purified by flash silica chromatography, elution gradient 0 to 60% EtOAc in hexanes. Product fractions were concentrated under reduced pressure to dryness to afford **15-(heptadecan-9-yloxy)-8,15-dioxopentadecanoic acid** (0.102 g, 27.8%) as a colorless oil. ^{1}H NMR (500 MHz, Chloroform-d) 0.84 - 0.94 (m, 6H), 1.20 - 1.70 (m, 44H), 2.24 - 2.45 (m, 8H), 4.87 (s, 1H).

d) 3-(((ethylimino)methylene)amino)-N,N-dimethylpropan-1-amine hydrochloride (0.054 g, 0.28 mmol) was added in one portion to a stirred solution of 15-(heptadecan-9-yloxy)-8,15-dioxopentadecanoic acid (0.071 g, 0.14 mmol)), nonan-1-ol (0.035 mL, 0.20 mmol), N-ethyl-N-isopropylpropan-2-amine (0.073 mL, 0.42 mmol), and N,N-dimethylpyridin-4-amine (3.31 mg, 0.03 mmol) in DCM (5 mL) at 0°C under argon. The resulting solution was stirred at room temperature for 16 hours. The reaction mixture was concentrated under reduced pressure to dryness and redissolved in EtOAc (20 mL) and sat. aq. $NaHCO_3$ (20 mL). The layers were separated, and the aqueous layer was extracted with EtOAc (3 x 30 mL). The organic layer was dried over $MgSO_4$, filtered and concentrated under reduced pressure to dryness to afford crude product. The resulting residue was purified by flash silica chromatography, elution gradient 0 to 60% EtOAc in hexanes. Product fractions were concentrated under reduced pressure to dryness to afford **1-(heptadecan-9-yl) 15-nonyl 8-oxopentadecanedioate** (0.063 g, 71.5%) as a colorless oil. ^{1}H NMR (500 MHz, Chloroform-*d*) 0.83 - 0.94 (m, 9H), 1.21 - 1.68 (m, 58H), 2.22 - 2.33 (m, 4H), 2.38 (s, 4H), 4.06 (s, 2H), 4.87 (s, 1H).

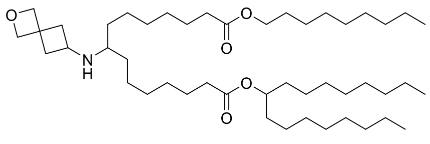

5

e) Sodium triacetoxyborohydride (0.062 g, 0.29 mmol) was added in one portion to a stirred solution of 2-oxaspiro[3.3]heptan-6-amine hydrochloride (0.043 g, 0.29 mmol) and 1-(heptadecan-9-yl) 15-nonyl 8-oxopentadecanedioate (0.063 g, 0.10 mmol) in 1,2-DCE (4 mL) and NMP (1 mL) at 0°C under argon. The resulting solution was stirred at room temperature for 16 hours. The reaction mixture was diluted with DCM (50 mL) and sat. aq. Na_2CO_3 (50 mL). The layers were separated, and the aqueous layer was extracted with DCM (3 x 25 mL). The organic layer was dried over $MgSO_4$, filtered and concentrated under reduced pressure to dryness to afford crude product. The resulting residue was purified by flash silica chromatography, elution gradient 0 to 100% of 20% MeOH/DCM (w/ 1% NH_4OH) in DCM. Product fractions were concentrated under reduced pressure to dryness to afford **5** (1-(heptadecan-9-yl) 15-nonyl 8-((2-oxaspiro[3.3]heptan-6-yl)amino)pentadecanedioate, 0.036 g, 49.7%) as a colorless oil. ^{1}H NMR (500 MHz, Chloroform-*d*) 0.82 - 0.96 (m, 9H), 1.28 (br s, 62H), 1.81 - 1.91 (m, 2H), 2.25 - 2.35 (m, 4H), 2.39 - 2.48 (m, 1H), 2.52 - 2.60 (m, 2H), 3.11 - 3.20 (m, 1H), 4.08 (s, 2H), 4.62 (s, 2H), 4.73 (s, 2H), 4.89 (s, 1H); $C_{47}H_{89}NO_5$ *m/z* calcd. 747.674 observed 748.8 $[M+H]^+$ (LCMS).

Lipid 6 Synthesis

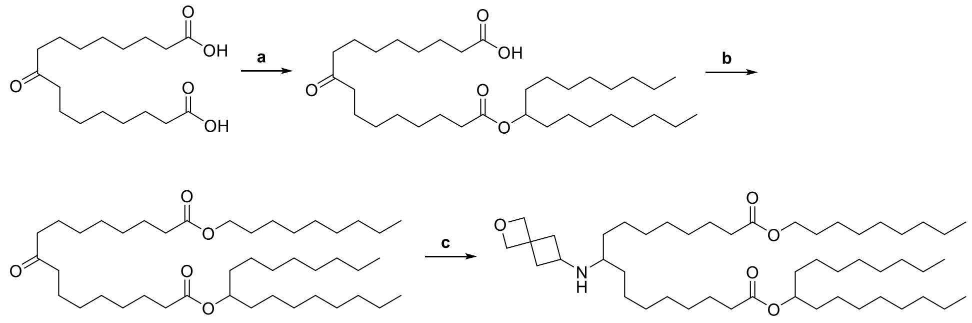

6

Reactants/Reagents: a) heptadecan-9-ol, EDC.HCl, DIPEA, DMAP, DCM; b) nonan-1-ol, EDC.HCl, DIPEA, DMAP, DCM c) 2-oxaspiro[3.3]heptan-6-aminium chloride, $NaBH(OAc)_3$, 1,2-DCE: NMP (4:1)

a) 3-(((ethylimino)methylene)amino)-N,N-dimethylpropan-1-amine hydrochloride (97 mg, 0.50 mmol) was added in one portion to a stirred solution of 9-oxoheptadecanedioic acid (105.6 mg, 0.34 mmol), N-ethyl-N-isopropylpropan-2-amine (176 µL, 1.01 mmol), heptadecan-9-ol (57.5 mg, 0.22 mmol) and N,N-dimethylpyridin-4-amine (8.62 mg, 0.07 mmol) in DCM (6 mL) at 0°C under argon. The resulting solution was stirred at 25 °C for 18 hours. The reaction mixture was concentrated under reduced pressure to dryness and redissolved in EtOAc (15 mL), water (5 mL) and 5% citric acid (10 mL). The layers were separated, and the aqueous layer was extracted three times with EtOAc (20 mL). The combined organic layers were dried over $MgSO_4$, filtered and concentrated under reduced pressure to dryness to afford crude product. The resulting residue was purified by flash silica chromatography, elution gradient 10 to 40% EtOAc in hexanes. Product fractions were concentrated under reduced pressure to dryness to afford **17-(heptadecan-9-yloxy)-9,17-dioxoheptadecanoic acid** (0.083 g, 44.4%) as a colorless oil. ^{1}H NMR (500 MHz, Chloroform-*d*) δ ppm 0.8 - 0.9 (m, 6 H) 1.2 - 1.3 (m, 36 H) 1.5 - 1.6 (m, 8 H) 1.6 - 1.6 (m, 4 H) 2.2 - 2.4 (m, 8 H) 4.8 - 4.9 (m, 1 H).

b) 3-(((ethylimino)methylene)amino)-N,N-dimethylpropan-1-amine hydrochloride (145 mg, 0.76 mmol) was added in one portion to a stirred solution of nonan-1-ol (124 µl, 0.71 mmol), N,N-dimethylpyridin-4-

amine (6.08 mg, 0.05 mmol), 17-(heptadecan-9-yloxy)-9,17-dioxoheptadecanoic acid (131 mg, 0.24 mmol) and N-ethyl-N-isopropylpropan-2-amine (174 µL, 1.00 mmol) in DCM (4.5 mL) at 0°C under argon. The resulting solution was stirred at 25 °C for 18 hours. The reaction mixture was diluted with DCM (10 mL) and sat. aq. NH_4Cl (10 mL). The layers were separated, and the aqueous layer was extracted three times with DCM (15 mL). The combined organic layers were dried over $MgSO_4$, filtered and concentrated under reduced pressure to dryness to afford crude product. The resulting residue was purified by flash silica chromatography, elution gradient 10 to 30% EtOAc in hexanes. Product fractions were concentrated under reduced pressure to afford **1-(heptadecan-9-yl) 17-nonyl 9-oxoheptadecanedioate** (0.135 g, 84%) as a colorless oil. ^{1}H NMR (500 MHz, Chloroform-*d*) δ ppm 0.9 - 0.9 (m, 9 H) 1.2 - 1.3 (m, 26 H) 1.3 (br s, 22 H) 1.5 - 1.7 (m, 14 H) 2.3 (q, *J*=7.3 Hz, 4 H) 2.4 (t, *J*=7.5 Hz, 4 H) 4.1 (t, *J*=6.8 Hz, 2 H) 4.8 - 4.9 (m, 1 H).

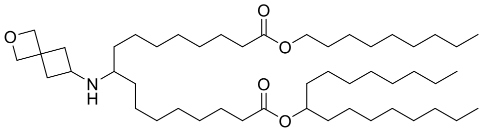

6

c) Sodium triacetoxyhydroborate (35.3 mg, 0.17 mmol) was added in one portion to a stirred solution of 1-(heptadecan-9-yl) 17-nonyl 9-oxoheptadecanedioate (45.3 mg, 0.07 mmol) and 2-oxaspiro[3.3]heptan-6-aminium chloride (23.95 mg, 0.16 mmol) in 1,2-DCE (2 mL) and NMP (0.5 mL) under argon. The resulting solution was stirred at 25 °C for 50 hours. The reaction mixture was diluted with DCM (10 mL), water (5 mL) and sat. aq. Na_2CO_3 (5 mL). The layers were separated, and the aqueous layer was extracted three times with DCM (15 mL). The combined organic layers were dried over $MgSO_4$, filtered and concentrated under reduced pressure to dryness to afford crude product. The resulting residue was purified by flash silica chromatography, elution gradient 0 to 50% of 20% MeOH/DCM (w/ 1% NH_4OH) in DCM. Product fractions were concentrated under reduced pressure to dryness to afford **6** (1-(heptadecan-9-yl) 17-nonyl 9-((2-oxaspiro[3.3]heptan-6-yl)amino)heptadecanedioate, 0.039 g, 75%) as a colorless oil. ^{1}H NMR (500 MHz, Methanol-*d*4) δ ppm 0.9 (br t, *J*=6.5 Hz, 9 H) 1.3 - 1.4 (m, 56 H) 1.5 - 1.6 (m, 4 H) 1.6 (br d, *J*=6.1 Hz, 6 H) 2.0 (br t, *J*=10.2 Hz, 2 H) 2.3 (br t, *J*=7.2 Hz, 4 H) 2.5 - 2.6 (m, 3 H) 3.2 (br t, *J*=7.8 Hz, 1 H) 4.1 (t, *J*=6.5 Hz, 2 H) 4.6 (s, 2 H) 4.7 (s, 2 H) 4.9 - 4.9 (m, 1 H); $C_{49}H_{93}NO_5$ *m/z* calcd. 775.705 observed 776.8 $[M+H]^+$ (LCMS).

Lipid 7 Synthesis

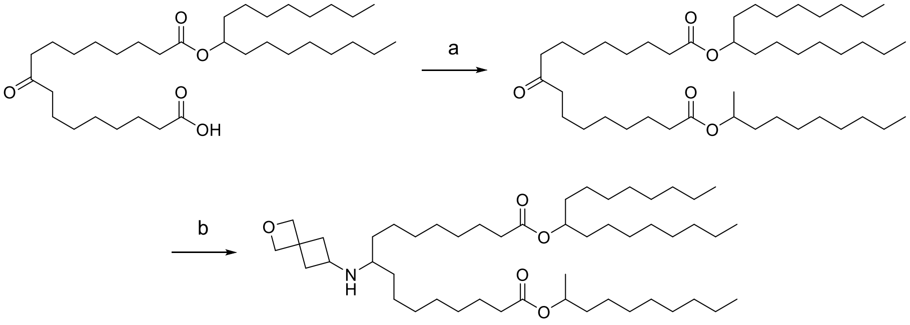

7

Reactants/Reagents: a) 9-heptadecanol, EDC.HCl, DIPEA, DMAP, DCM; b) 2-oxaspiro[3.3]heptan-6-aminium chloride, $NaBH(OAc)_3$, 1,2-DCE: NMP (4:1)

a) 3-(((ethylimino)methylene)amino)-N,N-dimethylpropan-1-amine hydrochloride (0.055 g, 0.29 mmol) was added in one portion to a stirred solution of 15-((2-heptylnonyl)oxy)-8,15-dioxopentadecanoic acid (0.0703 g, 0.14 mmol)), decan-2-ol (0.040 mL, 0.21 mmol), N-ethyl-N-isopropylpropan-2-amine (0.072 mL, 0.41 mmol), and N,N-dimethylpyridin-4-amine (3.36 mg, 0.03 mmol) in DCM (10 mL) at 0°C under argon. The resulting solution was stirred at room temperature for 16 hours. The reaction mixture was concentrated under reduced pressure to dryness and redissolved in EtOAc (20 mL) and sat. aq. $NaHCO_3$ (20 mL). The layers were separated, and the aqueous layer was extracted with EtOAc (3 x 30 mL). The organic layer was dried over $MgSO_4$, filtered and concentrated under reduced pressure to dryness to afford crude product. The resulting residue was purified by flash silica chromatography, elution gradient 0 to 60% EtOAc in hexanes. Product fractions were concentrated under reduced pressure to dryness to afford **1-(decan-2-yl) 15-(2-heptylnonyl) 8-oxopentadecanedioate** (0.041 g, 46.2 %) as a colorless oil. ^{1}H NMR (500 MHz, Chloroform-d, 21°C) δ ppm 0.84 - 0.93 (9H, m), 1.15 - 1.70 (58H, m), 2.23 - 2.32 (4H, m), 2.38 (4H, t), 3.94 - 3.99 (2H, d), 4.81 - 4.95 (1H, m).

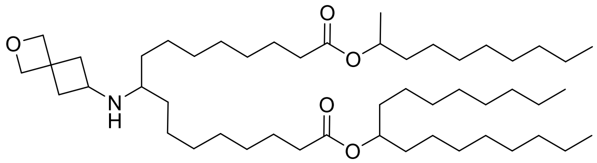

7

b) Sodium triacetoxyborohydride (0.040 g, 0.19 mmol) was added in one portion to a stirred solution of 2-oxaspiro[3.3]heptan-6-amine hydrochloride (0.028 g, 0.19 mmol) and 1-(decan-2-yl) 17-(heptadecan-9-yl) 9-oxoheptadecanedioate (0.044 g, 0.06 mmol) in 1,2-DCE (4 mL) and NMP (1 mL) at 0°C under argon. The resulting solution was stirred at room temperature for 16 hours. The reaction mixture was diluted with DCM (50 mL) and sat. aq. Na_2CO_3 (50 mL). The layers were separated, and the aqueous layer was extracted with DCM (3 x 25 mL). The organic layer was dried over $MgSO_4$, filtered and concentrated under reduced pressure to dryness to afford crude product. The resulting residue was purified by flash silica chromatography, elution gradient 0 to 100% of 20% MeOH/DCM (w/ 1% NH_4OH) in DCM. Product fractions were concentrated under reduced pressure to dryness to afford **7** (1-(decan-2-yl) 17-(heptadecan-9-yl) 9-((2-oxaspiro[3.3]heptan-6-yl)amino)heptadecanedioate, 0.032 g, 63.4 %) as a colorless oil. ^{1} H NMR (500 MHz, Methanol-d4) δ ppm 0.9 (t, *J*=6.9 Hz, 9 H) 1.1 - 1.7 (m, 69 H) 1.9 - 2.0 (m, 2 H) 2.3 - 2.3 (m, 4 H) 2.5 - 2.5 (m, 1 H) 2.5 - 2.6 (m, 2 H) 3.1 - 3.3 (m, 1 H) 4.6 (s, 2 H) 4.7 (s, 2 H) 4.9 - 4.9 (m, 2 H); $C_{57}H_{109}NO_5$ m/z calcd. 789.721 observed 790.7 [M+H]+ (LCMS).

Lipid 8 Synthesis

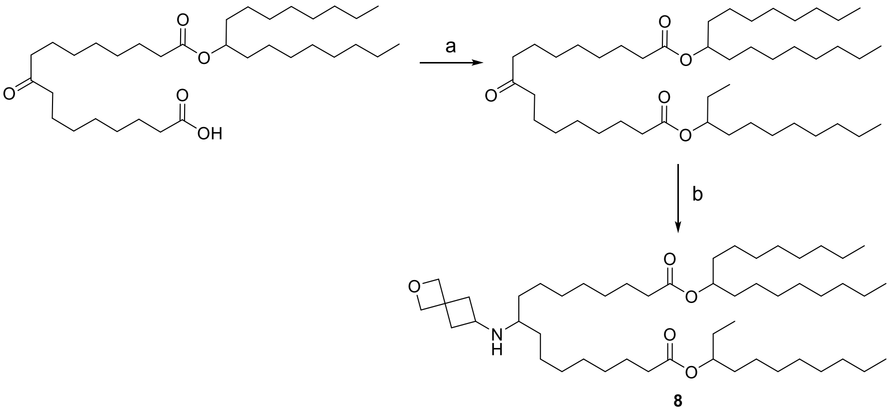

8

Reactants/Reagents: a) undecan-3-ol, EDC.HCl, DIPEA, DMAP, DCM; b) 2-oxaspiro[3.3]heptan-6-aminium chloride, $NaBH(OAc)_3$, 1,2-DCE: NMP (4:1)

a) 3-(((ethylimino)methylene)amino)-N,N-dimethylpropan-1-amine hydrochloride (0.146 g, 0.76 mmol) was added in one portion to a stirred mixture of 17-(heptadecan-9-yloxy)-9,17-dioxoheptadecanoic acid (0.2 g, 0.36 mmol), undecan-3-ol (0.093 g, 0.54 mmol), DIPEA (0.195 mL, 1.12 mmol), and DMAP (8.84 mg, 0.07 mmol) in DCM (5 mL) at 0°C under argon. The resulting mixture was stirred at room temperature for 16 hours. The reaction mixture was diluted with 10% citric acid (25 mL) and DCM (25 mL). The layers were separated, and the aqueous layer was extracted with DCM (4 x 25 mL). The combined organic layers were dried over $MgSO_4$, filtered and concentrated under reduced pressure to dryness to afford crude product. The resulting residue was purified by flash silica chromatography, elution gradient 0 to 40% EtOAc in hexanes. Product fractions were concentrated under reduced pressure to dryness to afford **1-(heptadecan-9-yl) 17-(undecan-3-yl) 9-oxoheptadecanedioate** (0.180 g, 70.4%) as a colorless oil. ^{1}H NMR (500 MHz, Chloroform-d, 27°C) δ ppm 0.89 (9H, t), 1.16 - 1.71 (67H, m), 2.23 - 2.30 (4H, m), 2.38 (4H, t), 4.82 - 4.95 (2H, m).

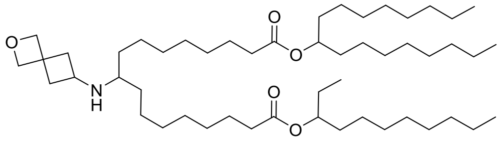

8

b) Sodium triacetoxyborohydride (0.037 g, 0.18 mmol) was added in one portion to a stirred solution of 2-oxaspiro[3.3]heptan-6-amine hydrochloride (0.023 g, 0.15 mmol) and 1-(heptadecan-9-yl) 17-(undecan-3-yl) 9-oxoheptadecanedioate (0.04 g, 0.06 mmol) in 1,2-DCE (2 mL) and NMP (0.5 mL) at 0°C under argon. The resulting solution was stirred at room temperature for 16 hours. The reaction mixture was diluted with DCM (50 mL) and sat. aq. Na_2CO_3 (50 mL). The layers were separated, and the aqueous layer was extracted with DCM (3 x 25 mL). The organic layer was dried over $MgSO_4$, filtered and concentrated under reduced pressure to dryness to afford crude product. The resulting residue was purified by flash silica chromatography, elution gradient 0 to 100% of 20% MeOH/DCM (w/ 1% NH_4OH) in DCM. Product fractions were concentrated under reduced pressure to dryness to afford **8** (1-(heptadecan-9-yl) 17-(undecan-3-yl) 9-((2-oxaspiro[3.3]heptan-6-yl)amino)heptadecanedioate, 0.027 g, 59.3%) as a colorless oil. ^{1}H NMR (500 MHz, Methanol-d4, 27°C) δ ppm 0.87 - 0.96 (12H, m), 1.26 - 1.70 (68H, m), 1.95 - 2.06

(2H, m), 2.29 - 2.37 (4H, m), 2.49 - 2.60 (3H, m), 3.18 - 3.29 (1H, m), 4.58 - 4.63 (2H, s), 4.73 (2H, s), 4.80 - 4.84 (1H, m), 4.87 - 4.94 (1H, m); $C_{51}H_{97}NO_5$ m/z calcd. 803.737 observed 804.72 $[M+H]^+$ (LCMS).

Lipid 9 Synthesis

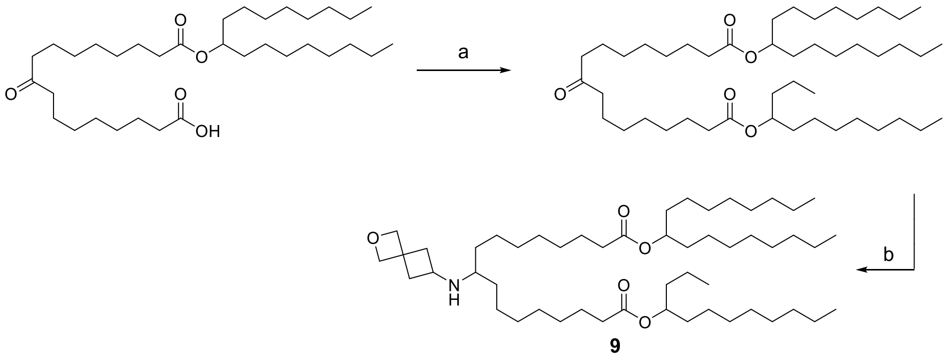

Reactants/Reagents: a) dodecan-4-ol, EDC.HCl, DIPEA, DMAP, DCM; b) 2-oxaspiro[3.3]heptan-6-aminium chloride, $NaBH(OAc)_3$, 1,2-DCE: NMP (4:1)

a) 3-(((ethylimino)methylene)amino)-N,N-dimethylpropan-1-amine hydrochloride (118 mg, 0.62 mmol) was added in one portion to a stirred solution of 17-(heptadecan-9-yloxy)-9,17-dioxoheptadecanoic acid (262 mg, 0.47 mmol), DIPEA (0.289 mL, 1.66 mmol), N,N-dimethylpyridin-4-amine (11.58 mg, 0.09 mmol) and dodecan-4-ol (106 mg, 0.57 mmol) in DCM (10 mL) at 0°C under argon. The resulting solution was stirred at RT for 16 hours. The reaction mixture was diluted with DCM (20 mL) and 10% citric acid solution (25 mL). The organic layer was separated, and the aqueous layer was extracted with DCM (3 x 25 mL). The combined organic layers were washed with sat. aq. NaCl (20 mL). The organic layer was dried over $MgSO_4$, filtered and concentrated under reduced pressure to dryness to afford crude product. The resulting residue was purified by flash silica chromatography, elution gradient 0 to 20% EtOAc in hexanes. Product fractions were concentrated under reduced pressure to dryness to afford **1-(dodecan-4-yl) 17-(heptadecan-9-yl) 9-oxoheptadecanedioate** (213 mg, 62.3%) as a colorless oil. ^1H NMR (500 MHz, Chloroform-d, 27°C) δ ppm 0.82 - 0.98 (12H, m), 1.18 - 1.68 (66H, m), 2.28 (4H, t), 2.34 - 2.43 (4H, t), 4.76 - 4.97 (2H, m).

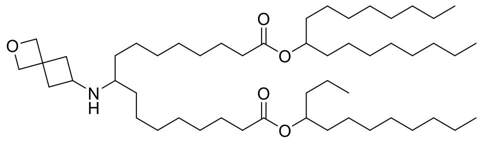

9

b) Sodium triacetoxyborohydride (23.80 mg, 0.11 mmol) was added in one portion to a stirred solution of 2-oxaspiro[3.3]heptan-6-amine hydrochloride (14.94 mg, 0.10 mmol) and 1-(dodecan-4-yl) 17-(heptadecan-9-yl) 9-oxoheptadecanedioate (30 mg, 0.04 mmol) in 1,2-DCE (2 mL) and NMP (0.5 mL) at 0°C under argon. The resulting solution was stirred at room temperature for 16 hours. The reaction mixture was diluted with DCM (50 mL) and sat. aq. Na_2CO_3 (50 mL). The layers were separated, and the aqueous layer was extracted with DCM (3 x 25 mL). The organic layer was dried over $MgSO_4$, filtered and concentrated under reduced pressure to dryness to afford crude product. The resulting residue was purified by flash silica chromatography, elution gradient 0 to 100% of 20% MeOH/DCM (w/ 1% NH_4OH) in

DCM. Product fractions were concentrated under reduced pressure to dryness to afford **9** (1-(dodecan-4-yl) 17-(heptadecan-9-yl) 9-((2-oxaspiro[3.3]heptan-6-yl)amino)heptadecanedioate, 10.5 mg, 30.8 %) as a colorless oil. ^{1}H NMR (500 MHz, Chloroform-d, 27°C) δ ppm 0.91 (12H, m), 1.23 - 1.68 (70H, m), 1.96 - 2.08 (2H, m), 2.25 - 2.36 (4H, t), 2.51 - 2.60 (3H, m), 3.21 - 3.28 (1H, m), 4.57 - 4.62 (2H, s), 4.72 (2H, s), 4.87 - 4.93 (2H, m); $C_{52}H_{99}NO_5$ m/z calcd. 817.752 observed 818.90 [M+H]+ (LCMS).

Lipid 10 Synthesis

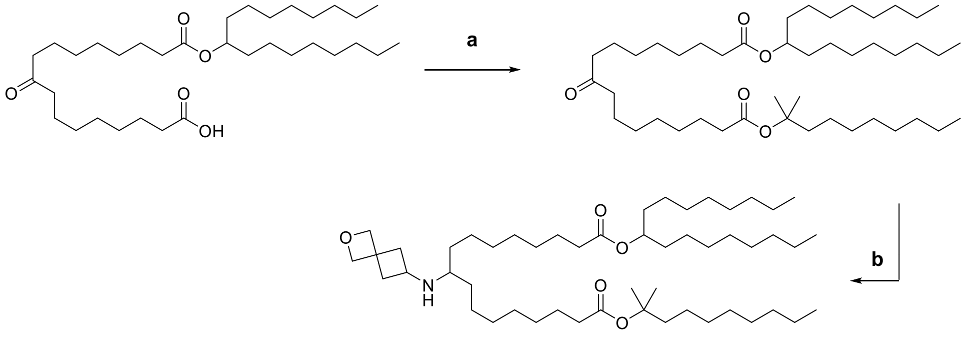

10

Reactants/Reagents: a) 2-methyldecan-2-ol, TFAA; b) 2-oxaspiro[3.3]heptan-6-amine hydrochloride, $NaBH(OAc)_3$, 1,2-DCE: NMP (4:1)

a) To a solution of 17-(heptadecan-9-yloxy)-9,17-dioxoheptadecanoic acid (28 mg, 0.05 mmol) in DCM (2 mL) at 0 °C, TFAA (0.016 mL, 0.11 mmol) was added dropwise. After 2.5 h, 2-methyldecan-2-ol (31.4 mg, 0.18 mmol) was slowly added. After 1 h the reaction was warmed to rt and allowed to stir for 2.5 h. The reaction was quenched with water and extracted with diethyl ether. The organic layer was separated and dried over $MgSO_4$, filtered and concentrated under reduced pressure to dryness to afford crude product. The residue was purified by silica gel chromatography with (0-10%) EtOAc in hexanes to obtain **1-(heptadecan-9-yl) 17-(2-methyldecan-2-yl) 9-oxoheptadecanedioate** (24.5 mg, 68.4%) as a pale yellow oil. ^{1}H NMR (500 MHz, Chloroform-d, 27°C) δ ppm 0.85 - 0.95 (9H, t), 1.22 - 1.37 (48H, m), 1.43 (6H, s), 1.58 (14H, m), 2.18 - 2.24 (2H, t), 2.26 - 2.31 (2H, t), 2.36 - 2.42 (4H, t), 4.82 - 4.95 (1H, m).

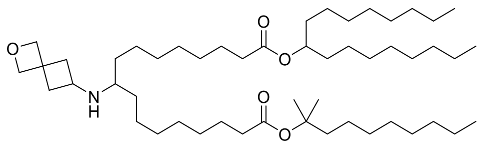

10

b) Sodium triacetoxyborohydride (19.83 mg, 0.09 mmol) was added in one portion to a stirred solution of 2-oxaspiro[3.3]heptan-6-amine hydrochloride (12.44 mg, 0.08 mmol) and 1-(heptadecan-9-yl) 17-(2-methyldecan-2-yl) 9-oxoheptadecanedioate (24.5 mg, 0.03 mmol) in 1,2-DCE (2 mL) and NMP (0.5 mL) at 0°C under argon. The resulting solution was stirred at room temperature for 16 hours. The reaction mixture was diluted with DCM (50 mL) and sat. aq. Na_2CO_3 (50 mL). The layers were separated, and the aqueous layer was extracted with DCM (3 x 25 mL). The organic layer was dried over $MgSO_4$, filtered and concentrated under reduced pressure to dryness to afford crude product. The resulting residue was

purified by flash silica chromatography, elution gradient 0 to 100% of 20% MeOH/DCM (w/ 1% NH_4OH) in DCM. Product fractions were concentrated under reduced pressure to dryness to afford **10** (1-(heptadecan-9-yl) 17-(2-methyldecan-2-yl) 9-((2-oxaspiro[3.3]heptan-6-yl)amino)heptadecanedioate, 18.2 mg, 65.3%) as a colorless oil. ^{1}H NMR (500 MHz, Methanol-d4, 27°C) δ ppm 0.92 (9H, t), 1.25 - 1.70 (70H, m), 1.73 - 1.82 (2H, m), 1.96 - 2.06 (2H, m), 2.20 - 2.26 (2H, m), 2.30 - 2.36 (2H, m), 2.49 - 2.54 (1H, m), 2.54 - 2.60 (2H, m), 3.18 - 3.27 (1H, m), 4.60 (2H, s), 4.73 (2H, s), 4.88 - 4.93 (1H, m); $C_{51}H_{97}NO_5$ m/z calcd. 803.737 observed 803.9 $[M]^+$ (LCMS).

Lipid 11 Synthesis

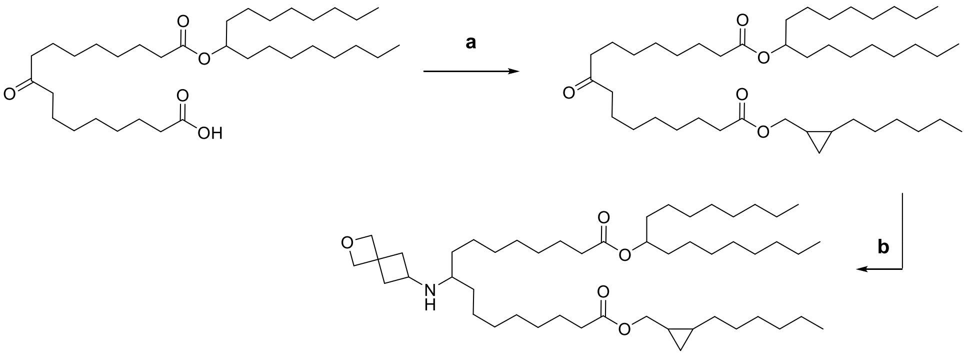

Reactants/Reagents: a) (2-hexylcyclopropyl)methanol, EDC.HCl, DIPEA, DMAP, DCM; b) 2-oxaspiro[3.3]heptan-6-aminium chloride, $NaBH(OAc)_3$, 1,2-DCE: NMP (4:1)

a) 3-(((ethylimino)methylene)amino)-N,N-dimethylpropan-1-amine hydrochloride (90 mg, 0.47 mmol) was added in one portion to a stirred solution of 17-(heptadecan-9-yloxy)-9,17-dioxoheptadecanoic acid (123 mg, 0.22 mmol), N-ethyl-N-isopropylpropan-2-amine (0.081 mL, 0.47 mmol), N,N-dimethylpyridin-4-amine (5.44 mg, 0.04 mmol) and (2-hexylcyclopropyl)methanol (41.7 mg, 0.27 mmol) in DCM (5 mL) at 0°C under argon. The resulting solution was stirred at RT for 16 hours. The reaction mixture was diluted with DCM 20 mL and 10% citric acid solution (25 mL). The organic layer was separated, and the aqueous layer was extracted with DCM (3 x 25 mL). The combined organic layers were washed with sat. aq. NaCl (20 mL). The organic layer was dried over $MgSO_4$, filtered and concentrated under reduced pressure to dryness to afford crude product. The resulting residue was purified by flash silica chromatography, elution gradient 0 to 40% EtOAc in hexanes. Product fractions were concentrated under reduced pressure to dryness to afford **1-(heptadecan-9-yl) 17-((2-hexylcyclopropyl)methyl) 9-oxoheptadecanedioate** (100 mg, 65.0 %) as a colorless oil. ^{1}H NMR (500 MHz, Chloroform-d, 27°C) δ ppm -0.02 - 0.09 (1H, m), 0.70 - 0.80 (1H, m), 0.84 - 0.97 (10H, m), 1.09 - 1.70 (59H, m), 2.24 - 2.46 (8H, m), 3.88 - 4.27 (2H, m), 4.88 (1H, m).

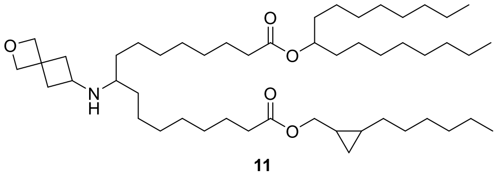

11

b) Sodium triacetoxyborohydride (20.70 mg, 0.10 mmol) was added in one portion to a stirred solution of 2-oxaspiro[3.3]heptan-6-amine hydrochloride (12.99 mg, 0.09 mmol) and 1-(heptadecan-9-yl) 17-((2-hexylcyclopropyl)methyl) 9-oxoheptadecanedioate (25 mg, 0.04 mmol) in 1,2-DCE (2 mL) and NMP (0.5 mL) at 0°C under argon. The resulting solution was stirred at room temperature for 16 hours. The reaction mixture was diluted with DCM (50 mL) and sat. aq. Na_2CO_3 (50 mL). The layers were separated, and the aqueous layer was extracted with DCM (3 x 25 mL). The organic layer was dried over MgSO4, filtered and concentrated under reduced pressure to dryness to afford crude product. The resulting residue was purified by flash silica chromatography, elution gradient 0 to 100% of 20% MeOH/DCM (w/ 1% NH_4OH) in DCM. Product fractions were concentrated under reduced pressure to afford **11** (1-(heptadecan-9-yl) 17-((2-hexylcyclopropyl)methyl) 9-((2-oxaspiro[3.3]heptan-6-yl)amino)heptadecanedioate**,** 10.4 mg, 36.5%) as a colorless oil. ^{1}H NMR (500MHz, Methanol-*d*4) -0.01 - 0.08 (m, 1H), 0.71 - 0.80 (m, 1H), 0.85 - 0.96 (m, 10H), 1.10 - 1.70 (m, 63H), 1.96 - 2.08 (m, 2H), 2.31 (d, *J* = 7.3 Hz, 4H), 2.50 - 2.62 (m, 3H), 3.25 (br t, *J* = 7.9 Hz, 1H), 3.85 - 3.96 (m, 1H), 4.20 - 4.29 (m, 1H), 4.59 (s, 2H), 4.72 (s, 2H), 4.87 (br d, J = 2.3 Hz, 1H);$C_{50}H_{93}NO_5$ m/z calcd. 787.705 observed 788.9 $[M+H]^+$ (LCMS).

Lipid 12 Synthesis

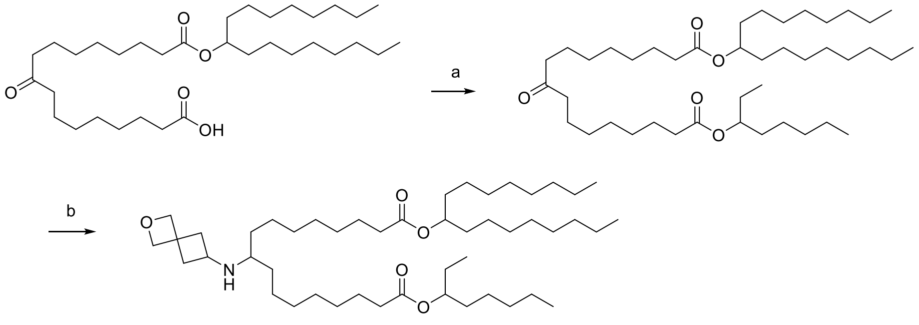

Reactants/Reagents: a) octan-3-ol, EDC.HCl, DIPEA, DMAP, DCM; b) 2-oxaspiro[3.3]heptan-6-aminium chloride, $NaBH(OAc)_3$, 1,2-DCE: NMP (4:1)

a) 3-(((ethylimino)methylene)amino)-N,N-dimethylpropan-1-amine hydrochloride (52.0 mg, 0.27 mmol) was added in one portion to a stirred solution of 17-(heptadecan-9-yloxy)-9,17-dioxoheptadecanoic acid (70.7 mg, 0.13 mmol), N-ethyl-N-isopropylpropan-2-amine (0.080 mL, 0.46 mmol), N,N-dimethylpyridin-4-amine (2.343 mg, 0.02 mmol) and octan-3-ol (0.043 mL, 0.27 mmol) in DCM (3 mL) at 0°C under argon. The resulting solution was stirred at 25 °C for 16 hours. The reaction mixture was diluted with EtOAc (20 mL), water (5 mL) and 5% citric acid solution (15 mL). The layers were separated, and the aqueous layer was extracted with EtOAc (3 x 20 mL). The combined organic layers were washed with sat. aq. NaCl (20 mL). The organic layer was dried over $MgSO_4$, filtered and concentrated under reduced pressure to afford crude product. The resulting residue was purified by flash silica chromatography, elution gradient 0 to 35% EtOAc in hexanes. Product fractions were concentrated under reduced pressure to afford **1-(heptadecan-9-yl) 17-(octan-3-yl) 9-oxoheptadecanedioate** (52.6 mg, 61.8%) as a colorless oil. ^{1}H NMR (500MHz, Chloroform-d) 0.84 - 0.91 (m, 12H), 1.21 - 1.33 (m, 42H), 1.46 - 1.64 (m, 16H), 2.27 (td, *J* = 7.5, 5.3 Hz, 4H), 2.37 (t, *J* = 7.4 Hz, 4H), 4.78 - 4.89 (m, 2H).

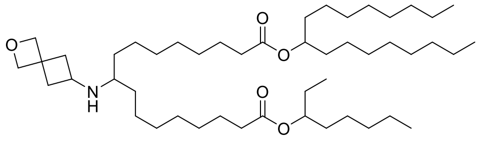

12

b) Sodium triacetoxyhydroborate (50.3 mg, 0.24 mmol) was added in one portion (after 10 min) to a stirred solution of 1-(heptadecan-9-yl) 17-(octan-3-yl) 9-oxoheptadecanedioate (52.6 mg, 0.08 mmol), and 2-oxaspiro[3.3]heptan-6-aminium chloride (34.3 mg, 0.23 mmol) in 1,2-DCE (2 mL) and NMP (0.5 mL) under argon. The resulting solution was stirred at 25 °C for 18 hours. The reaction mixture was diluted with DCM (15 mL), water (5 mL) and sat. aq. Na_2CO_3 (5 mL). The layers were separated, and the aqueous layer was extracted with DCM (3 x 15 mL). The combined organic layers were dried over $MgSO_4$, filtered and evaporated to afford crude product. The resulting residue was purified by flash silica chromatography, elution gradient 0 to 40% of 20% MeOH in DCM (w/ 1% NH_4OH) in DCM. Product fractions were concentrated under reduced pressure to afford **12** (1-(heptadecan-9-yl) 17-(octan-3-yl) 9-((2-oxaspiro[3.3]heptan-6-yl)amino)heptadecanedioate, 25.9 mg, 43.0%) as a colorless oil. ^{1}H NMR (400MHz, Methanol-d4) 0.88 - 0.95 (m, 12H), 1.26 - 1.39 (m, 50H), 1.50 - 1.68 (m, 12H), 1.94 - 2.02 (m, 2H), 2.33 (td, *J* = 7.2, 2.2 Hz, 4H), 2.46 - 2.59 (m, 3H), 3.19 (t, *J* = 7.8 Hz, 1H), 4.60 (s, 2H), 4.73 (s, 2H), 4.76 - 4.83 (m, 1H), 4.88 - 4.94 (m, 1H); $C_{48}H_{91}NO_5$ m/z calcd. 761.690 observed 762.6 $[M+H]^+$ (LCMS).

Lipid 13 Synthesis:

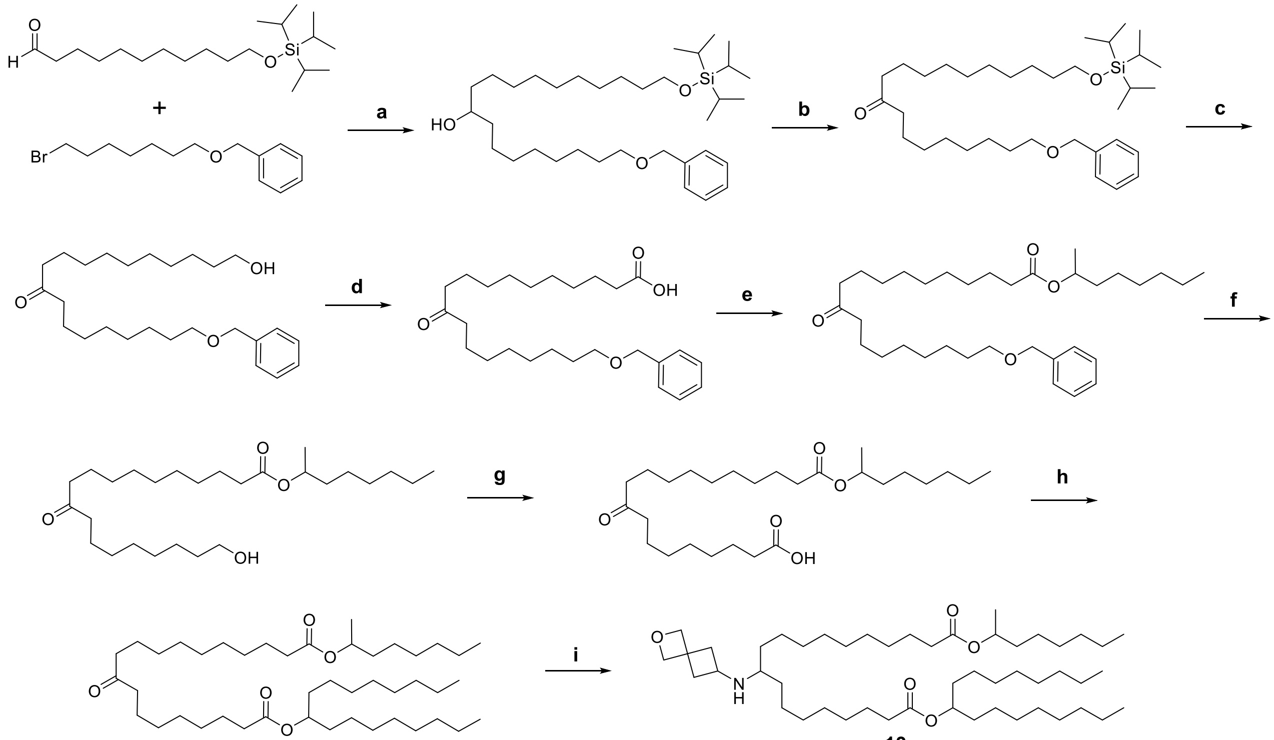

13

Reactants/Reagents: a) Mg, I_2, THF; b) Pyridine sulfur trioxide, TEA; c) TBAF, THF; d) DMP, $NaHCO_3$, DCM and then $NaClO_2$, t-BuOH, 2-methyl-2-butene, NaH_2PO_4; e) octan-2-ol, EDC.HCl, DIPEA, DMAP, DCM; f) Pd/C, H_2; g) DMP, $NaHCO_3$, DCM and then

$NaClO_2$, t-BuOH, 2-methyl-2-butene, NaH_2PO_4; h) heptadecan-9-ol, EDC.HCl, DIPEA, DMAP, DCM; i) 2-oxaspiro[3.3]heptan-6-amine hydrochloride, $NaBH(OAc)_3$, 1,2-DCE: NMP (4:1).

a) To a suspension of magnesium (0.284 g, 11.67 mmol) turnings in DMF (20 mL) containing a small iodine crystal were added few drops of (((8-bromooctyl)oxy)methyl)benzene in THF (10 mL). The mixture was heated until the reaction started, then the remaining (((8-bromooctyl)oxy)methyl)benzene (2.096 g, 7.00 mmol) was added drop by drop to maintain a non-assisted gentle reflux. After complete addition of the starting material, the mixture was heated under reflux for 1 h. The solution of Grignard reagent was cooled to 25 °C. 11-((triisopropylsilyl)oxy)undecanal (2 g, 5.84 mmol) was added in one portion to the stirred mixture under argon. The resulting mixture was stirred at 70°C for 16 hours. The reaction mixture was quenched with water (50 mL), extracted with DCM (3 x 25 mL), the organic layer was dried over $MgSO_4$, filtered and concentrated under reduced pressure to afford a pale yellow oil. The resulting residue was purified by flash silica chromatography, elution gradient 0 to 40% EtOAc in hexanes. Product fractions were concentrated under reduced pressure to afford **1-(benzyloxy)-19-((triisopropylsilyl)oxy)nonadecan-9-ol** (1.837 g, 74.4%) as a colorless oil. ^{1}H NMR (500 MHz, Chloroform-d, 27°C) δ ppm 1.03 - 1.14 (21H, m), 1.26 - 1.47 (28H, m), 1.53 - 1.58 (2H, m), 1.60 - 1.67 (2H, m), 3.44 - 3.54 (2H, t), 3.54 - 3.63 (1H, m), 3.65 - 3.73 (2H, t), 4.53 (2H, s), 7.36 (5H, m).

b) To an oven-dried flask, 1-(benzyloxy)-19-((triisopropylsilyl)oxy)nonadecan-9-ol (2.477 g, 4.40 mmol) was dissolved in DCM (80 mL). DMSO (15 mL) was then added, followed by TEA (6.13 mL, 44.00 mmol) to the reaction mixture. The mixture was cool to RT. Pyridine sulfur trioxide was added to the mixture and the reaction was allowed to warm to room temperature. The reaction mixture was stirred for 1 hour at room temperature. The reaction mixture was diluted with DCM and the reaction mixture was quenched with sat. aq. NH_4Cl (100 mL). The layers were separated and the aqueous layer was extracted with EtOAc (3 x 50 mL), the combined organic layers were washed with brine (50 mL) and dried over $MgSO_4$, filtered, and concentrated under reduced pressure to afford pale yellow oil. The resulting residue was purified by flash silica chromatography, elution gradient 0 to 20% EtOAc in hexanes. Product fractions were concentrated under reduced pressure to afford **1-(benzyloxy)-19-((triisopropylsilyl)oxy)nonadecan-9-one** (1.837 g, 74.4%) as a colorless oil. ^{1}H NMR (500 MHz, Chloroform-d, 27°C) δ ppm 1.03 - 1.18 (21H, m), 1.29 (28H, m), 2.33 - 2.44 (4H, t), 3.42 - 3.55 (2H, t), 3.62 - 3.73 (2H, t), 4.52 (2H, s), 7.36 (5H, m).

c) Tetrabutylammonium fluoride (13.10 mL, 13.10 mmol) was added dropwise to a stirred solution of 1-(benzyloxy)-19-((triisopropylsilyl)oxy)nonadecan-9-one (1.837 g, 3.27 mmol) in THF (10 mL) at 0°C under argon. The resulting mixture was stirred at 25 °C for 16 hours. The reaction mixture was quenched with sat aq. NH_4Cl (50 mL) and extracted with EtOAc (3 x 50 mL). Subsequently, the organic layer was dried over $MgSO_4$, filtered, and then concentrated under reduced pressure to afford an orange oil. The resulting residue was purified by flash silica chromatography, elution gradient 0 to 40% EtOAc in hexanes. Product fractions were concentrated under reduced pressure to afford **1-(benzyloxy)-19-hydroxynonadecan-9-one** (1.266 g, 96%) as a colorless oil. ^{1}H NMR (500 MHz, Chloroform-*d*, 27°C) δ ppm 1.23 - 1.70 (30H, m), 2.40 (4H, t), 3.48 (2H, t), 3.66 (2H, t), 4.52 (2H, s), 7.30 - 7.42 (5H, m).

d) *i)* 3-oxo-1l5-benzo[d][1,2]iodaoxole-1,1,1(3H)-triyl triacetate (3.98 g, 9.39 mmol) was added in one portion to a stirred suspension of sodium hydrogen carbonate (2.365 g, 28.16 mmol) and 1-(benzyloxy)-19-hydroxynonadecan-9-one (1.266 g, 3.13 mmol) in DCM (20 mL) at 0°C. The resulting solution was allowed to come to 25 °C over 24 hours. The reaction mixture was diluted with DCM (20 mL) and washed sequentially with sat. aq. $NaHCO_3$ (20 mL) and sat. aq. $Na_2S_2O_3$ (20 mL) The organic layer was dried over

$MgSO_4$, filtered, and concentrated under reduced pressure to afford crude aldehyde precursor as a colorless dry film, which was used without further purification. *ii)* The crude product was added to a stirred solution of 2-methylbut-2-ene (9.94 mL, 93.86 mmol), sodium dihydrogen phosphate (2.252 g, 18.77 mmol), and sodium chlorite (1.698 g, 18.77 mmol) in THF (10 mL) and tert-butanol (5.00 mL) at 25°C. The resulting solution was stirred at 25°C for 4 hours. The reaction mixture was diluted with DCM and water (30 ml each). The reaction mixture was adjusted to pH 3 with 1M HCl solution. The organic layer was dried over $MgSO_4$, filtered and concentrated under reduced pressure to afford crude product. The resulting residue was purified by flash silica chromatography, elution gradient 0 to 100% EtOAc in hexanes. Product fractions were concentrated under reduced pressure to afford product **19-(benzyloxy)-11-oxononadecanoic acid** (1.275 g, 97%) as a white solid. ^1H NMR (500 MHz, Chloroform-d, 27°C) δ ppm 1.30 (26H, m), 2.28 - 2.50 (6H, m), 3.37 - 3.60 (2H, t), 4.53 (2H, s), 7.25 - 7.38 (5H, m).

e) EDC.HCl (311 mg, 1.62 mmol) was added in one portion to a stirred mixture of 19-(benzyloxy)-11-oxononadecanoic acid (400 mg, 0.96 mmol), octan-2-ol (0.195 mL, 1.24 mmol), N-ethyl-N-isopropylpropan-2-amine (0.350 mL, 2.01 mmol), and DMAP (23.35 mg, 0.19 mmol) in DCM (5 mL) at 0°C under argon. The resulting mixture was stirred at room temperature for 16 hours. The reaction mixture was diluted with sat. aq. $NaHCO_3$ (25 mL) and DCM (25 mL). The layers were separated, and the aqueous layer was extracted with DCM (4 x 25 mL). The combined organic layers were dried over $MgSO_4$, filtered, and concentrated under reduced pressure to afford crude product. The resulting residue was purified by flash silica chromatography, elution gradient 0 to 40% EtOAc in hexanes. Product fractions were concentrated under reduced pressure to afford **octan-2-yl 19-(benzyloxy)-11-oxononadecanoate** (410 mg, 81%) as a colorless oil. ^1H NMR (500 MHz, Chloroform-d, 27°C) δ ppm 0.83 - 0.97 (3H, m), 1.15 - 1.67 (39H, m), 2.18 - 2.31 (2H, m), 2.39 (4H, t), 3.39 - 3.54 (2H, m), 4.52 (2H, s), 4.80 - 5.00 (1H, m), 7.28 - 7.40 (5H, m).

f) Octan-2-yl 19-(benzyloxy)-11-oxononadecanoate (410 mg, 0.77 mmol) and Pd/C (247 mg, 0.23 mmol) in MeOH (10 mL) was stirred under an atmosphere of hydrogen and RT for 16 hours. The resulting residue was purified by flash silica chromatography, elution gradient 0 to 60% EtOAc in hexanes. Product fractions were concentrated under reduced pressure to afford **octan-2-yl 19-hydroxy-11-oxononadecanoate** (200 mg, 58.8%) as a pale yellow oil. ^1H NMR (500 MHz, Chloroform-d, 27°C) δ ppm 0.92 (3H, t), 1.17 - 1.68 (39H, m), 2.22 - 2.32 (2H, m), 2.40 (4H, t), 3.55 - 3.67 (2H, m), 4.90 (1H, m).

g) *i)* DMP (577 mg, 1.36 mmol) was added in one portion to a stirred suspension of sodium bicarbonate (343 mg, 4.08 mmol) and octan-2-yl 19-hydroxy-11-oxononadecanoate (200 mg, 0.45 mmol) in DCM (5 mL) at 0°C. The resulting solution was allowed to come to room temp over 24 hours. The reaction mixture was diluted with DCM (20 mL), and washed sequentially with sat. aq. NaHCO3 (20 mL), and sat. aq. $Na_2S_2O_3$ (20 mL) The organic layer was dried over $MgSO_4$, filtered, and concentrated under reduced pressure to afford crude aldehyde precursor as a colorless dry film, which was used without further purification. *ii)* The crude product was added to a stirred solution of 2-methylbut-2-ene (1.442 mL, 13.61 mmol), sodium dihydrogen phosphate (327 mg, 2.72 mmol), and sodium chlorite (246 mg, 2.72 mmol) in THF (10 mL) and tert-butanol (5.00 mL) at 25°C. The resulting solution was stirred at RT for 4 hours. The reaction mixture was diluted with DCM and water (30 ml each). The reaction mixture was adjusted to pH =3 with 1M HCl solution. The organic layer was dried over $MgSO_4$, filtered, and concentrated under reduced pressure to afford crude product. The resulting residue was purified by flash silica chromatography, elution gradient 0 to 100% EtOAc in hexanes. Product fractions were concentrated under reduced pressure to afford desired product **19-(octan-2-yloxy)-9,19-dioxononadecanoic acid** as a white solid. Yield was not

measured. ^{1}H NMR (500 MHz, Chloroform-*d*, 27°C) δ ppm 0.92 (3H, t), 1.24 - 1.72 (38H, m), 2.29 (2H, t), 2.35 - 2.47 (6H, m), 4.87 (1H, m).

h) EDC.HCl (227 mg, 1.18 mmol) was added in one portion to a stirred mixture of 19-(octan-2-yloxy)-9,19-dioxononadecanoic acid (256 mg, 0.56 mmol), heptadecan-9-ol (217 mg, 0.84 mmol), N,N-dimethylpyridin-4-amine (0.403 mL, 2.31 mmol), and N,N-dimethylpyridin-4-amine (13.76 mg, 0.11 mmol) in DCM (5 mL) at 0°C under argon. The resulting mixture was stirred at room temperature for 16 hours. The reaction mixture was diluted with sat. aq. $NaHCO_3$ (25 mL) and DCM (25 mL). The layers were separated, and the aqueous layer was extracted with DCM (4 x 25 mL). The combined organic layers were dried over $MgSO_4$, filtered, and concentrated under reduced pressure to afford crude product. The resulting residue was purified by flash silica chromatography, elution gradient 0 to 40% EtOAc in hexanes. Product fractions were concentrated under reduced pressure to afford **1-(heptadecan-9-yl) 19-(octan-2-yl) 9-oxononadecanedioate** (141 mg, 36.1 %) as a colorless oil. ^{1}H NMR (500 MHz, Chloroform-*d*, 27°C) δ ppm 0.86 - 0.95 (9H, m), 1.18 - 1.69 (65H, m), 2.29 (4H, m), 2.35 - 2.44 (4H, m), 4.80 - 4.98 (2H, m).

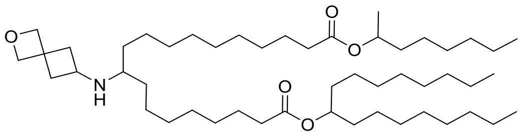

13

i) Sodium triacetoxyborohydride (0.116 g, 0.55 mmol) was added in one portion to a stirred solution of 1-(heptadecan-9-yl) 19-(octan-2-yl) 9-oxononadecanedioate (0.141 g, 0.20 mmol) and 2-oxaspiro[3.3]heptan-6-amine hydrochloride (0.073 g, 0.49 mmol) in 1,2-DCE (2 mL) and NMP (0.5 mL) at 0°C under argon. The resulting solution was stirred at room temperature for 16 hours. The reaction mixture was diluted with DCM (50 mL) and sat. aq. Na_2CO_3 (50 mL). The layers were separated, and the aqueous layer was extracted with DCM (3 x 25 mL). The organic layer was dried over $MgSO_4$, filtered, and concentrated under reduced pressure to afford crude product. The resulting residue was purified by flash silica chromatography, elution gradient 0 to 100% of 20% MeOH in DCM (w/ 1% NH_4OH) in DCM. Product fractions were concentrated under reduced pressure to afford **13** (1-(heptadecan-9-yl) 19-(octan-2-yl) 9-((2-oxaspiro[3.3]heptan-6-yl)amino)nonadecanedioate, 0.099 g, 61.7%) as a colorless oil. ^{1}H NMR (500MHz, Methanol-d4) 0.92 (s, 9H), 1.18 - 1.71 (m, 71H), 1.91 - 2.04 (m, 2H), 2.27 - 2.37 (m, 4H), 2.43 - 2.50 (m, 1H), 2.52 - 2.59 (m, 2H), 3.11 - 3.23 (m, 1H), 4.56 - 4.64 (m, 2H), 4.73 (s, 2H); $C_{49}H_{93}NO_5$ m/z calcd. 789.721 observed 790.80 $[M+H]^+$ (LCMS).

Lipid 14 Synthesis

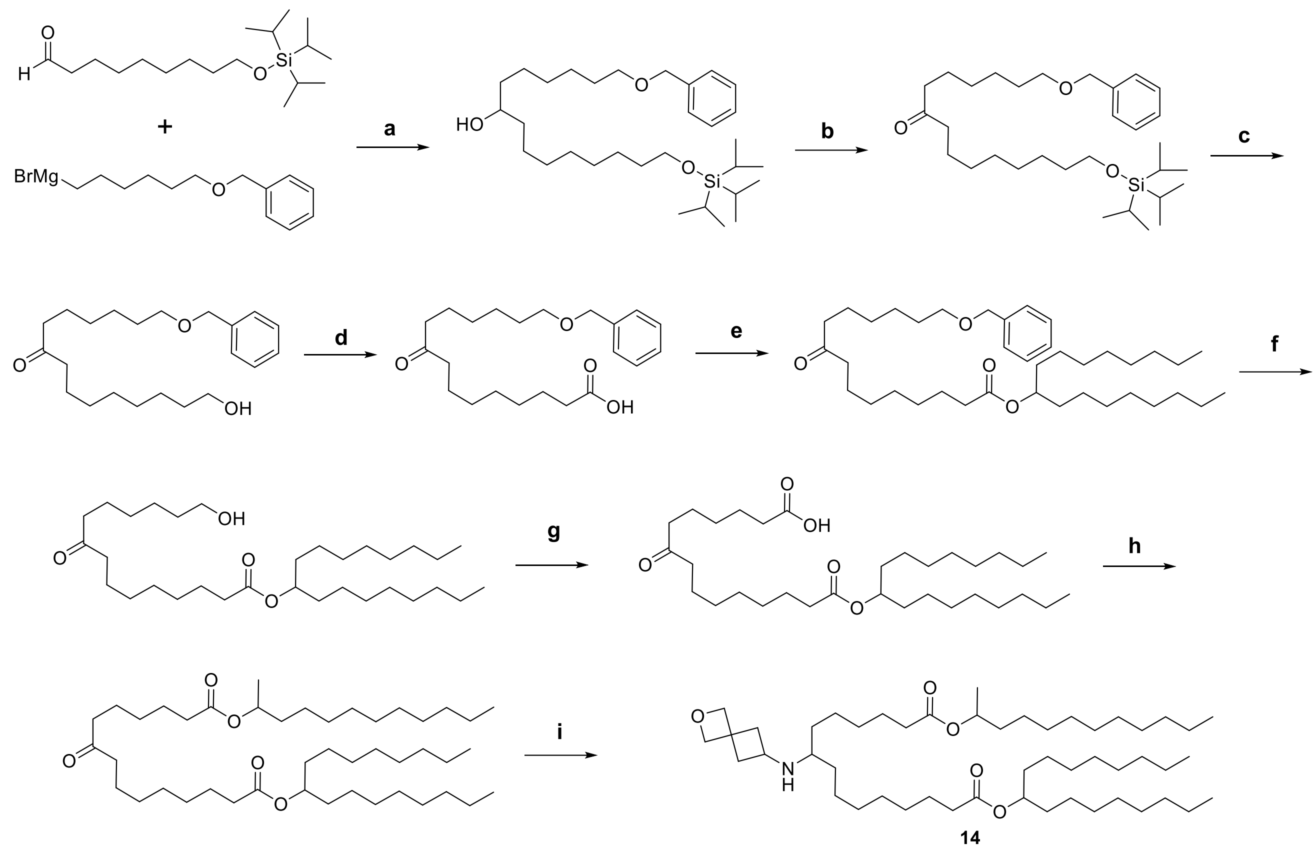

Reactants/Reagents: a) Mg, I_2, THF; b) Pyridine sulfur trioxide, TEA; c) TBAF, THF; d) DMP, $NaHCO_3$, DCM and then $NaClO_2$, *t*-BuOH, 2-methyl-2-butene, NaH_2PO_4; e) octan-2-ol, EDC.HCl, DIPEA, DMAP, DCM; f) Pd/C, H_2; g) DMP, $NaHCO_3$, DCM and then $NaClO_2$, *t*-BuOH, 2-methyl-2-butene, NaH_2PO_4; h) heptadecan-9-ol, EDC.HCl, DIPEA, DMAP, DCM; i) 2-oxaspiro[3.3]heptan-6-amine hydrochloride, $NaBH(OAc)_3$, 1,2-DCE: NMP (4:1).

a) (6-(benzyloxy)hexyl)magnesium bromide (5.79 mL, 2.89 mmol) was diluted in THF (10 mL) containing a small crystal of iodine. The solution of Grignard reagent was cooled to 0°C and 9-((triisopropylsilyl)oxy)nonanal (0.7 g, 2.23 mmol) was added in one portion to the stirred mixture under argon. The resulting mixture was stirred at 70°C for 16 hours. The reaction mixture was quenched with water (50 mL), extracted with DCM (3 x 25 mL), the organic layer was dried over $MgSO_4$, filtered, and concentrated under reduced pressure to afford pale yellow oil. The resulting residue was purified by flash silica chromatography, elution gradient 0 to 40% EtOAc in hexanes. Product fractions were concentrated under reduced pressure to afford **1-(benzyloxy)-15-((triisopropylsilyl)oxy)pentadecan-7-ol** (0.550 g, 49 %) as a colorless oil. ^{1}H NMR (500 MHz, Chloroform-*d*, 27°C) δ ppm 1.04 - 1.13 (21H, m), 1.25 - 1.49 (20H, m), 1.51 - 1.60 (2H, m), 1.60 - 1.69 (2H, m), 3.43 - 3.53 (2H, t), 3.55 - 3.63 (1H, m), 3.66 - 3.73 (2H, t), 4.53 (2H, s), 7.36 (5H, m).

b) To an oven-dried flask was added 1-(benzyloxy)-15-((triisopropylsilyl)oxy)pentadecan-7-ol (0.860 g, 1.70 mmol) and DCM (40 mL). DMSO (7.50 mL) was then added, followed by triethylamine (2.365 mL, 16.97 mmol) to the reaction mixture. The mixture was cool to 0 C. pyridine sulfur trioxide (2.160 g, 13.57 mmol) was added to the mixture and the reaction was allowed to warm to room temperature. The reaction mixture was stirred for 1 hour at room temperature. The reaction mixture was diluted with DCM

and the reaction mixture was quenched with sat. aq. NH_4Cl (100 mL). Layer were separated and the aqueous layer was extracted with EtOAc (3 x 50 mL), the combined organic layers were washed with brine (50 mL) and dried over $MgSO_4$, filtered and concentrated under reduced pressure to afford pale yellow oil. The resulting residue was purified by flash silica chromatography, elution gradient 0 to 20% EtOAc in hexanes. Product fractions were concentrated under reduced pressure to afford **1-(benzyloxy)-15-((triisopropylsilyl)oxy)pentadecan-7-one** (0.684 g, 80%) as a colorless oil. ^1H NMR (500 MHz, Chloroform-*d*, 27°C) δ ppm 0.98 - 1.15 (21H, m), 1.25 - 1.71 (20H, m), 2.32 - 2.50 (4H, m), 3.42 - 3.52 (2H, t), 3.62 - 3.74 (2H, t), 4.52 (2H, s), 7.36 (5H, m).

c) Tetrabutylammonium fluoride (5.42 mL, 5.42 mmol) was added dropwise to a stirred solution of 1-(benzyloxy)-15-((triisopropylsilyl)oxy)pentadecan-7-one (0.684 g, 1.35 mmol) in THF (10 mL) at 0°C under argon. The resulting mixture was stirred at RT for 16 hours. The reaction mixture was quenched with sat. aq. NH_4Cl (50 mL), extracted with EtOAc (3 x 50 mL), the organic layer was dried over $MgSO_4$, filtered and concentrated under reduced pressure to afford orange oil. The resulting residue was purified by flash silica chromatography, elution gradient 0 to 40% EtOAc in hexanes. Product fractions were concentrated under reduced pressure to afford **1-(benzyloxy)-15-hydroxypentadecan-7-one** (0.385 g, 82 %) as a colorless oil. ^1H NMR (500 MHz, Chloroform-*d*, 27°C) δ ppm 1.23 - 1.70 (20H, m), 2.40 (4H, t), 3.48 (2H, t), 3.66 (2H, t), 4.52 (2H, s), 7.30 - 7.42 (5H, m).

d) *i)* 3-oxo-1l5-benzo[d][1,2]iodaoxole-1,1,1(3H)-triyl triacetate (1.515 g, 3.57 mmol) was added in one portion to a stirred suspension of sodium bicarbonate (0.900 g, 10.72 mmol) and 1-(benzyloxy)-15-hydroxypentadecan-7-one (0.415 g, 1.19 mmol) in DCM (10 mL) at 0°C. The resulting solution was allowed to come to room temp over 24 hours. The reaction mixture was diluted with DCM (20 mL), and washed sequentially with saturated aqueous $NaHCO_3$ (20 mL), and sat. aq. $Na_2S_2O_3$ (20 mL) The organic layer was dried over $MgSO_4$, filtered, and concentrated under reduced pressure to afford crude aldehyde precursor as a colorless dry film, which was used without further purification. *ii)* The crude product was added to a stirred solution of 2-methylbut-2-ene (3.78 mL, 35.72 mmol), sodium dihydrogen phosphate (0.857 g, 7.14 mmol) and sodium chlorite (0.646 g, 7.14 mmol) in THF (10.00 mL) and tBuOH (5 mL) at 25°C. The resulting solution was stirred at RT for 4 hours. The reaction mixture was diluted with DCM and water (30 ml each). The reaction mixture was adjusted to pH =3 with 1M HCl solution. The organic layer was dried over $MgSO_4$, filtered and concentrated under reduced pressure to afford crude product. The resulting residue was purified by flash silica chromatography, elution gradient 0 to 100% EtOAc in hexanes. Product fractions were concentrated under reduced pressure to afford desired product **15-(benzyloxy)-9-oxopentadecanoic acid** (0.433 g, 100%) as a white solid. ^1H NMR (500 MHz, Chloroform-*d*, 27°C) δ ppm 1.29 (18H, m), 2.39 (6H, m), 3.48 (2H t), 4.52 (2H, s), 7.30 - 7.42 (5H, m).

e) 3-(((ethylimino)methylene)amino)-N,N-dimethylpropan-1-amine hydrochloride (389 mg, 2.03 mmol) was added in one portion to a stirred mixture of 15-(benzyloxy)-9-oxopentadecanoic acid (433 mg, 1.19 mmol), heptadecan-9-ol (398 mg, 1.55 mmol), N-ethyl-N-isopropylpropan-2-amine (0.438 mL, 2.51 mmol), and N,N-dimethylpyridin-4-amine (29.2 mg, 0.24 mmol) in DCM (5 mL) at 0°C under argon. The resulting mixture was stirred at room temperature for 16 hours. The reaction mixture was diluted with sat. aq. $NaHCO_3$ (25 mL) and DCM (25 mL). The layers were separated, and the aqueous layer was extracted with DCM (4 x 25 mL). The combined organic layers were dried over $MgSO_4$, filtered and concentrated under reduced pressure to afford crude product. The resulting residue was purified by flash silica chromatography, elution gradient 0 to 40% EtOAc in hexanes. Product fractions were concentrated under reduced pressure to afford **heptadecan-9-yl 15-(benzyloxy)-9-oxopentadecanoate** (276 mg,

38.4%) as a colorless oil. ^{1}H NMR (500 MHz, Chloroform-d, 27°C) δ ppm 0.83 - 0.95 (6H, t), 1.21 - 1.70 (46H, m), 2.29 (2H, t), 2.36 - 2.45 (4H, m), 3.42 - 3.53 (2H, t), 4.52 (2H, s), 4.89 (1H, m), 7.35 (5H, m).

f) Heptadecan-9-yl 15-(benzyloxy)-9-oxopentadecanoate (276 mg, 0.46 mmol) and Pd/C (147 mg, 0.14 mmol) in MeOH (10 mL) was stirred under an atmosphere of hydrogen for 16 hours. The resulting residue was purified by flash silica chromatography, elution gradient 0 to 60% EtOAc in hexanes. Product fractions were concentrated under reduced pressure to afford **heptadecan-9-yl 15-hydroxy-9-oxopentadecanoate** (190 mg, 80%) as a pale yellow oil. ^{1}H NMR (500 MHz, Chloroform-d, 27°C) δ ppm 0.92 (6H, t), 1.23 - 1.71 (46H, m), 2.24 - 2.32 (2H, t), 2.37 - 2.45 (4H, m), 3.54 - 3.70 (2H, m), 4.87 (1H, m).

g) *i)* 3-oxo-1l5-benzo[d][1,2]iodaoxole-1,1,1(3H)-triyl triacetate (473 mg, 1.12 mmol) was added in one portion to a stirred suspension of sodium bicarbonate (281 mg, 3.35 mmol) and heptadecan-9-yl 15-hydroxy-9-oxopentadecanoate (190 mg, 0.37 mmol) in DCM (10 mL) at 0°C. The resulting solution was allowed to come to 25°C over 24 hours. The reaction mixture was diluted with DCM (20 mL), and washed sequentially with sat. aq. $NaHCO_3$ (20 mL), and sat. aq. $Na_2S_2O_3$ (20 mL) The organic layer was dried over $MgSO_4$, filtered and concentrated under reduced pressure to afford crude aldehyde precursor as a colorless dry film, which was used without further purification. *ii)* The crude product was added to a stirred solution of 2-methylbut-2-ene (1.182 mL, 11.16 mmol), sodium dihydrogen phosphate (268 mg, 2.23 mmol), and sodium bicarbonate (281 mg, 3.35 mmol) in THF (10.00 mL) and *tert*-Butanol (5 mL) at 25°C. The resulting solution was stirred at RT for 4 hours. The reaction mixture was diluted with DCM and water (30 ml each). The reaction mixture was adjusted to pH 3 with 1M HCl solution. The organic layer was dried over $MgSO_4$, filtered, and concentrated under reduced pressure to afford crude product. The resulting residue was purified by flash silica chromatography, elution gradient 0 to 100% EtOAc in hexanes. Product fractions were concentrated under reduced pressure to afford **15-(benzyloxy)-9-oxopentadecanoic acid** (0.433 g, 100 %) as a white solid. ^{1}H NMR (500 MHz, Chloroform-*d*, 27°C) δ ppm 0.92 (6H, t), 1.24 - 1.72 (44H, m), 2.29 (2H, t), 2.35 - 2.47 (6H, m), 4.87 (1H, m).

h) 3-(((ethylimino)methylene)amino)-N,N-dimethylpropan-1-amine hydrochloride (153 mg, 0.80 mmol) was added in one portion to a stirred mixture of 15-(heptadecan-9-yloxy)-7,15-dioxopentadecanoic acid (200 mg, 0.38 mmol), dodecan-2-ol (0.128 mL, 0.57 mmol), N-ethyl-N-isopropylpropan-2-amine (0.273 mL, 1.56 mmol), and N,N-dimethylpyridin-4-amine (9.31 mg, 0.08 mmol) in DCM (5 mL) at 0°C under argon. The resulting mixture was stirred at room temperature for 16 hours. The reaction mixture was diluted with sat. aq. $NaHCO_3$ (25 mL) and DCM (25 mL). The layers were separated, and the aqueous layer was extracted with DCM (4 x 25 mL). The combined organic layers were dried over $MgSO_4$, filtered, and concentrated under reduced pressure to afford crude product. The resulting residue was purified by flash silica chromatography, elution gradient 0 to 40% EtOAc in hexanes. Product fractions were concentrated under reduced pressure to afford **1-(dodecan-2-yl) 15-(heptadecan-9-yl) 7-oxopentadecanedioate** (125 mg, 47%) as a colorless oil. ^{1}H NMR (500 MHz, Chloroform-*d*, 27°C) δ ppm 0.90 (9H, t), 1.17 - 1.72 (66H, m), 2.29 (4H, t), 2.40 (4H, m), 4.90 (1H, m).

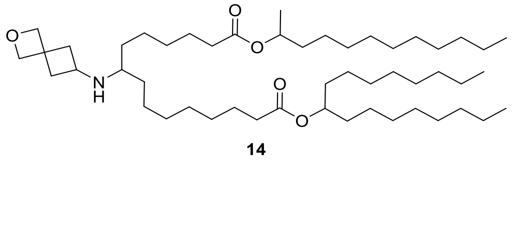

14

i) Sodium triacetoxyborohydride (103 mg, 0.49 mmol) was added in one portion to a stirred solution of 1-(dodecan-2-yl) 15-(heptadecan-9-yl) 7-oxopentadecanedioate (125 mg, 0.18 mmol) and 2-oxaspiro[3.3]heptan-6-amine hydrochloride (64.8 mg, 0.43 mmol) in 1,2-DCE (2 mL) and NMP (0.5 mL) at 0°C under argon. The resulting solution was stirred at room temperature for 16 hours. The reaction mixture was diluted with DCM (50 mL) and sat. aq. Na_2CO_3 (50 mL). The layers were separated, and the aqueous layer was extracted with DCM (3 x 25 mL). The organic layer was dried over $MgSO_4$, filtered and concentrated under reduced pressure to afford crude product. The resulting residue was purified by flash silica chromatography, elution gradient 0 to 100% of 20% MeOH in DCM (w/ 1% NH_4OH) in DCM. Product fractions were concentrated under reduced pressure to afford **14** (1-(dodecan-2-yl) 15-(heptadecan-9-yl) 7-((2-oxaspiro[3.3]heptan-6-yl)amino)pentadecanedioate, 85 mg, 59.9 %) as a colorless oil. ^1H NMR (500 MHz, Methanol-*d*4, 27°C) δ ppm 0.92 (9H, t), 1.31 (71H, m), 1.91 - 2.05 (2H, m), 2.26 - 2.37 (4H, m), 2.43 - 2.50 (1H, m), 2.52 - 2.60 (2H, m), 3.11 - 3.23 (1H, m), 4.53 - 4.63 (2H, s), 4.69 - 4.78 (2H, s), 4.91 (2H, m); $C_{49}H_{93}NO_5$ m/z calcd. 789.721 observed 790.80 $[M+H]^+$ (LCMS).

Lipid 15 Synthesis:

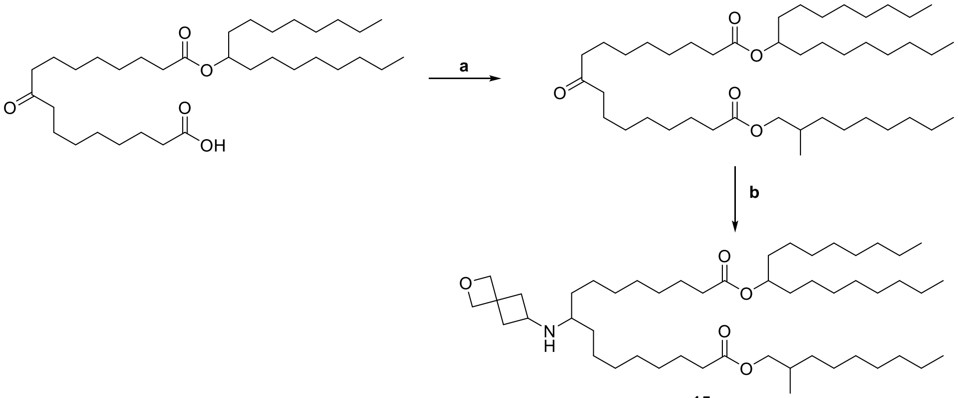

15

Reagents: a) EDC.HCl, DIPEA, DMAP, DCM; b) 2-oxaspiro[3.3]heptan-6-amine hydrochloride, $NaBH(OAc)_3$, 1,2-DCE: NMP (4:1)

a) 3-(((ethylimino)methylene)amino)-N,N-dimethylpropan-1-amine hydrochloride (66.8 mg, 0.35 mmol) was added in one portion to a stirred solution of 2-methylnonan-1-ol (52.5 mg, 0.33 mmol), N-ethyl-N-isopropylpropan-2-amine (0.104 mL, 0.60 mmol), N,N-dimethylpyridin-4-amine (3.04 mg, 0.02 mmol) and 17-(heptadecan-9-yloxy)-9,17-dioxoheptadecanoic acid (91.7 mg, 0.17 mmol) in DCM (4 mL) at 0°C under argon. The resulting solution was stirred at 25 °C for 16 hours. The reaction mixture was diluted with DCM (20 mL), 5% Citric acid solution (20 mL) . The layers were separated, and the aqueous layer was extracted with DCM (3 x 20 mL). The combined organic layers were washed with sat**.** aq**.** NaCl (20 mL). The organic layer was dried over $MgSO_4$, filtered and concentrated under reduced pressure to afford crude product. The resulting residue was purified by flash silica chromatography, elution gradient 0 to 35% EtOAc in hexanes. Product fractions were concentrated under reduced pressure to afford **1-(heptadecan-9-yl) 17-(2-methylnonyl) 9-oxoheptadecanedioate** (87 mg, 76 %) as a colorless oil. ^1H NMR (500 MHz, Chloroform-*d*) δ ppm 0.8 - 0.9 (m, 12 H) 1.2 - 1.4 (m, 48 H) 1.4 - 1.7 (m, 13 H) 2.3 (dt, J=12.4, 7.5 Hz, 4 H) 2.4 (t, J=7.5 Hz, 4 H) 3.8 - 4.0 (m, 2 H) 4.8 - 4.9 (m, 1 H).

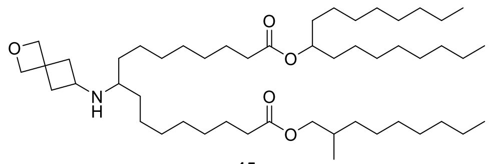

15

b) Sodium triacetoxyhydroborate (30.7 mg, 0.14 mmol) was added in one portion (after 10 min) to a stirred solution of 1-(heptadecan-9-yl) 17-(2-methylnonyl) 9-oxoheptadecanedioate (40.2 mg, 0.06 mmol) and 2-oxaspiro[3.3]heptan-6-aminium chloride (20.83 mg, 0.14 mmol) in 1,2-DCE (2 mL) and NMP (0.5 mL) under argon. The resulting solution was stirred at 25 °C for 18 hours. The reaction mixture was diluted with DCM (15 mL), water (5 mL) and sat. aq. Na_2CO_3 (10 mL). The layers were separated, and the aqueous layer was extracted with DCM (3 x 15 mL). The combined organic layers were dried over $MgSO_4$, filtered and evaporated to afford crude product. The resulting residue was purified by flash silica chromatography, elution gradient 0 to 40% of 20% MeOH in DCM (w/ 1% NH_4OH) in DCM. Product fractions were concentrated under reduced pressure to afford **15** (1-(heptadecan-9-yl) 17-(2-methylnonyl) 9-((2-oxaspiro[3.3]heptan-6-yl)amino)heptadecanedioate, 12.10 mg, 26.4 %) as a colorless oil. ^{1}H NMR (500 MHz, Methanol-*d*4) δ ppm 0.9 - 1.0 (m, 12 H) 1.3 - 1.4 (m, 56 H) 1.5 - 1.6 (m, 4 H) 1.6 - 1.7 (m, 4 H) 1.7 - 1.8 (m, 1 H) 2.0 - 2.0 (m, 2 H) 2.3 - 2.3 (m, 4 H) 2.5 - 2.6 (m, 3 H) 3.2 - 3.2 (m, 1 H) 3.9 - 4.0 (m, 2 H) 4.6 - 4.6 (m, 2 H) 4.7 - 4.7 (m, 2 H) 4.9 - 4.9 (m, 1 H); $C_{50}H_{95}NO_5$ m/z calcd. 789.721 observed 790.8 $[M+H]^+$ (LCMS).

Lipid 16 Synthesis:

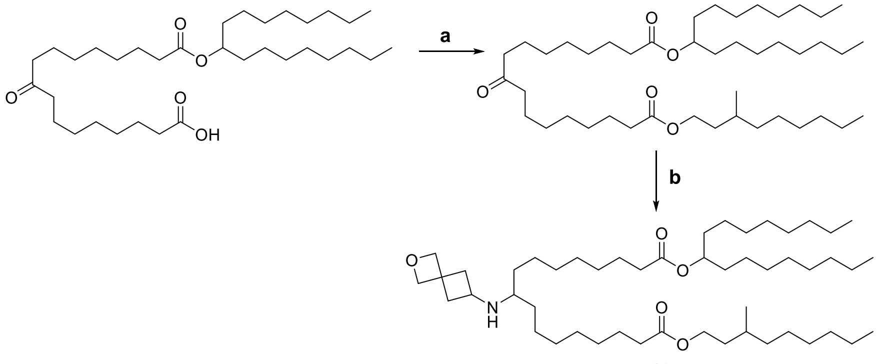

16

Reagents: a) EDC.HCl, DIPEA, DMAP, DCM; b) 2-oxaspiro[3.3]heptan-6-aminium chloride, $NaBH(OAc)_3$, 1,2-DCE: NMP (4:1)

a) 3-(((ethylimino)methylene)amino)-N,N-dimethylpropan-1-amine hydrochloride (58.3 mg, 0.30 mmol) was added in one portion to a stirred mixture of 17-(heptadecan-9-yloxy)-9,17-dioxoheptadecanoic acid (80 mg, 0.14 mmol), 3-methylnonan-1-ol (0.047 mL, 0.22 mmol), N-ethyl-N-isopropylpropan-2-amine (0.104 mL, 0.59 mmol), and N,N-dimethylpyridin-4-amine (3.5 mg, 0.03 mmol) in DCM (5 mL) at 0°C under argon. The resulting mixture was stirred at room temperature for 16 hours. The reaction mixture was diluted with DCM (25 mL) and 10% citric acid (25 mL). The layers were separated, and the aqueous layer was extracted with DCM (3 x 25 mL). The combined organic layers were dried over $MgSO_4$, filtered and concentrated under reduced pressure to afford crude product. The resulting residue was purified by flash silica chromatography, elution gradient 0 to 60% EtOAc in hexanes. Product fractions were concentrated

under reduced pressure to afford **1-(heptadecan-9-yl) 17-(3-methylnonyl) 9-oxoheptadecanedioate** (61.9 mg, 61.7 %) as a white solid. ^{1}H NMR (500 MHz, Chloroform-*d*, 27°C) δ ppm 0.82 - 0.97 (12H, m), 1.10 - 1.70 (61H, m), 2.25 - 2.31 (4H, m), 2.38 (4H, t), 4.02 - 4.18 (2H, m), 4.80 - 4.93 (1H, m).

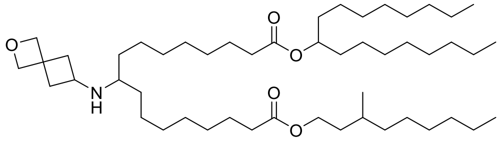

16

b) Sodium triacetoxyborohydride (24.77 mg, 0.12 mmol) was added in one portion to a stirred solution of 2-oxaspiro[3.3]heptan-6-amine hydrochloride (15.54 mg, 0.10 mmol) and 1-(heptadecan-9-yl) 17-(3-methylnonyl) 9-oxoheptadecanedioate (30 mg, 0.04 mmol) in 1,2-DCE (2 mL) and NMP (0.5 mL) at 0°C under argon. The resulting solution was stirred at room temperature for 16 hours. The reaction mixture was diluted with DCM (50 mL) and sat. aq. Na_2CO_3 (50 mL). The layers were separated, and the aqueous layer was extracted with DCM (3 x 25 mL). The organic layer was dried over $MgSO_4$, filtered and concentrated under reduced pressure to afford crude product. The resulting residue was purified by flash silica chromatography, elution gradient 0 to 100% of 20% MeOH in DCM (w/ 1% NH_4OH) in DCM. Product fractions were concentrated under reduced pressure to afford **16** (1-(heptadecan-9-yl) 17-(3-methylnonyl) 9-((2-oxaspiro[3.3]heptan-6-yl)amino)heptadecanedioate, 16.90 mg, 49.4 %) as a colorless oil. ^{1}H NMR (500 MHz, Methanol-*d4*) δ ppm 0.9 - 0.9 (m, 12 H) 1.1 - 1.7 (m, 65 H) 2.0 (br dd, *J*=11.7, 9.2 Hz, 2 H) 2.3 (t, *J*=7.3 Hz, 4 H) 2.5 - 2.6 (m, 3 H) 3.2 - 3.3 (m, 1 H) 4.1 - 4.2 (m, 2 H) 4.6 (s, 2 H) 4.7 (s, 2 H) 4.9 - 4.9 (m, 1 H); $C_{50}H_{95}NO_5$ m/z calcd. 789.721 observed 790.7 $[M+H]^+$ (LCMS).

Lipid 17 Synthesis:

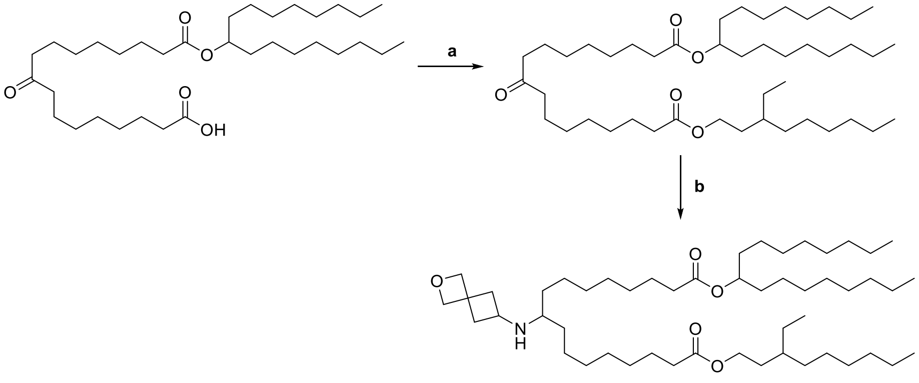

17

Reagents: a) EDC.HCl, DIPEA, DMAP, DCM; b) 2-oxaspiro[3.3]heptan-6-aminium chloride, $NaBH(OAc)_3$, 1,2-DCE: NMP (4:1)

a) 3-(((ethylimino)methylene)amino)-N,N-dimethylpropan-1-amine hydrochloride (0.058 g, 0.30 mmol) was added in one portion to a stirred mixture of 17-((3-ethylnonyl)oxy)-9,17-dioxoheptadecanoic acid (0.067 g, 0.14 mmol), heptadecan-9-ol (0.055 g, 0.21 mmol), N-ethyl-N-isopropylpropan-2-amine (0.102 mL, 0.59 mmol), and N,N-dimethylpyridin-4-amine (3.49 mg, 0.03 mmol) in DCM (5 mL) at 0°C under argon. The resulting mixture was stirred at room temperature for 16 hours. The reaction mixture was

diluted with sodium bicarbonate (25 mL) and DCM (25 mL). The layers were separated, and the aqueous layer was extracted with DCM (4 x 25 mL). The combined organic layers were dried over $MgSO_4$, filtered and concentrated under reduced pressure to afford crude product. The resulting residue was purified by flash silica chromatography, elution gradient 0 to 40% EtOAc in hexanes. Product fractions were concentrated under reduced pressure to afford **1-(3-ethylnonyl) 17-(heptadecan-9-yl) 9-oxoheptadecanedioate** (0.040 g, 39.6 %) as a colorless oil. ^{1}H NMR (500 MHz, Chloroform-*d*, 27°C) δ ppm 0.81 - 0.92 (12H, m), 1.17 - 1.68 (63H, m), 2.21 - 2.31 (4H, m), 2.34 - 2.41 (4H, t), 3.98 - 4.14 (2H, t), 4.79 - 4.91 (1H, m).

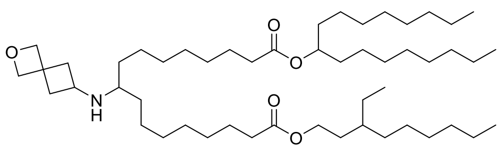

17

b) Sodium triacetoxyborohydride (0.032 g, 0.15 mmol) was added in one portion to a stirred solution of 1-(3-ethylnonyl) 17-(heptadecan-9-yl) 9-oxoheptadecanedioate (0.040 g, 0.06 mmol) and 1-(3-ethylnonyl) 17-(heptadecan-9-yl) 9-oxoheptadecanedioate (0.040 g, 0.06 mmol) in 1,2-DCE (2 mL) and NMP (0.5 mL) at 0°C under argon. The resulting solution was stirred at room temperature for 16 hours. The reaction mixture was diluted with DCM (50 mL) and sat. aq. Na_2CO_3 (50 mL). The layers were separated, and the aqueous layer was extracted with DCM (3 x 25 mL). The organic layer was dried over $MgSO_4$, filtered and concentrated under reduced pressure to afford crude product. The resulting residue was purified by flash silica chromatography, elution gradient of 20% MeOH in DCM (w/ 1% NH_4OH) in DCM. Product fractions were concentrated under reduced pressure to afford **17** (1-(3-ethylnonyl) 17-(heptadecan-9-yl) 9-((2-oxaspiro[3.3]heptan-6-yl)amino)heptadecanedioate, 0.029 g, 64.4 %) as a colorless oil. ^{1}H NMR (500 MHz, Methanol-*d*4) 0.91 (br t, J = 2.9 Hz, 12H), 1.22 - 1.69 (m, 67H), 1.96 - 2.05 (m, 2H), 2.31 (s, 4H), 2.49 - 2.59 (m, 3H), 3.17 - 3.27 (m, 1H), 4.07 - 4.15 (m, 2H), 4.59 (s, 2H), 4.72 (s, 2H), 4.84 - 4.91 (m, 1H)$C_{50}H_{95}NO_5$ m/z calcd. 803.737 observed 804.6 $[M+H]^+$ (LCMS).

References:

1. A. Gallud, A. Sabirsh, M. Ughetto WO2024211518A1, 2024.

2. M. J. Munson, G. O'Driscoll, A. M. Silva, E. Lázaro-Ibáñez, A. Gallud, J. T. Wilson, A. Collén, E. K. Esbjörner and A. Sabirsh, *Commun. Biol.*, 2021, **4**, 211.

3. S.M. Ansell, X. Du, WO2013086322A1, 2013.

^{1}H NMR (500 MHz, Methanol-*d*4) of **1**

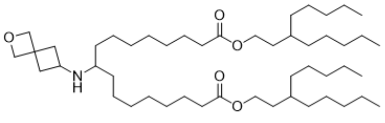

1

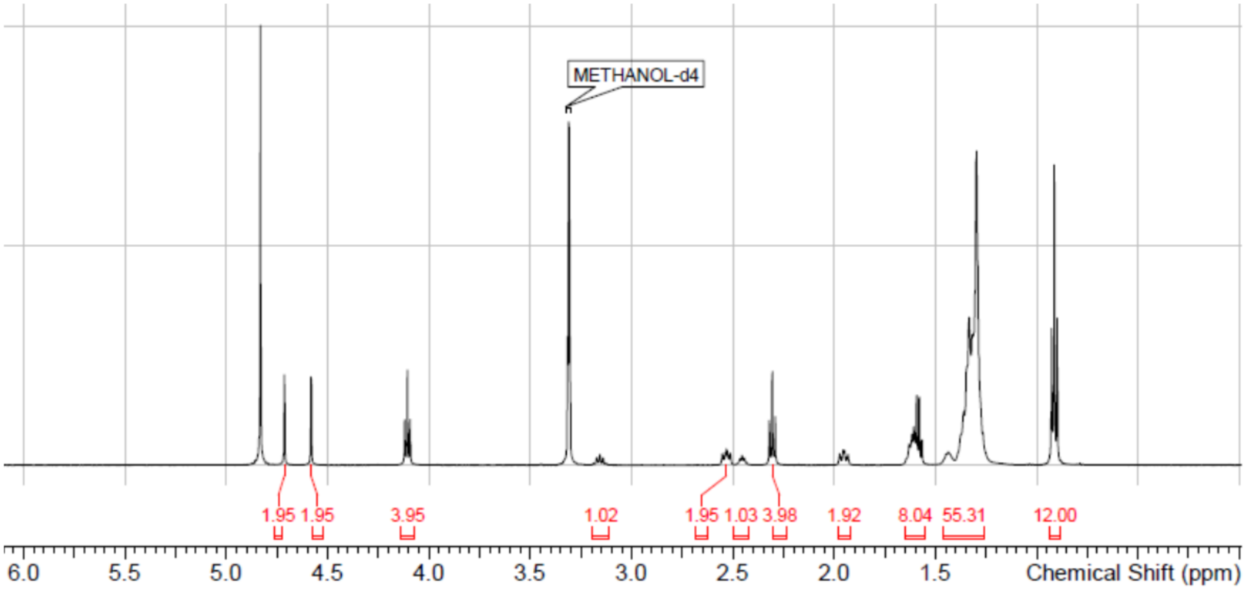

HSQC (500 MHz, Methanol-*d*4) of **1**

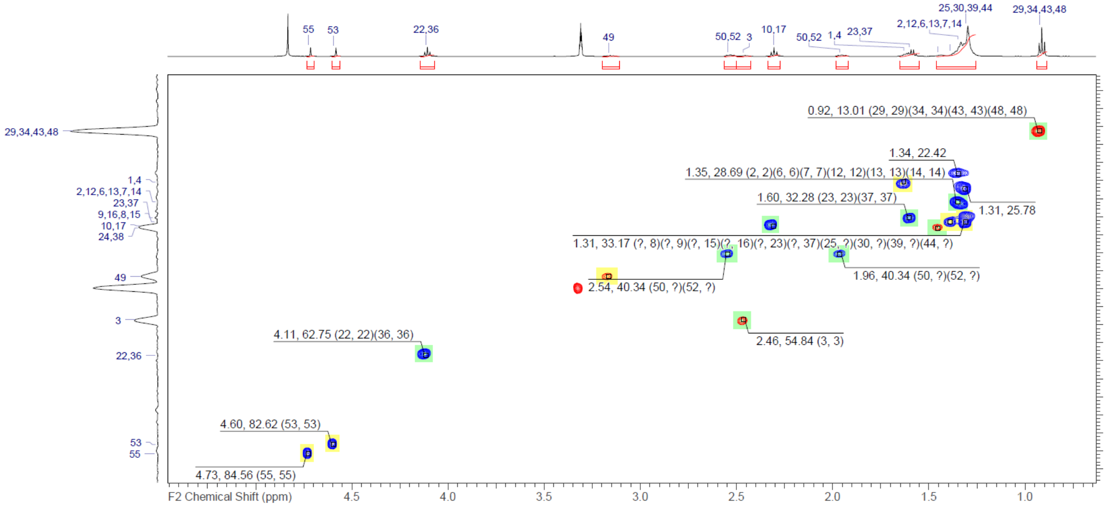

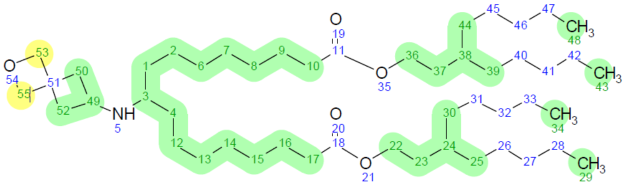

^{1}H NMR (500 MHz, Methanol-*d*4) of **2**

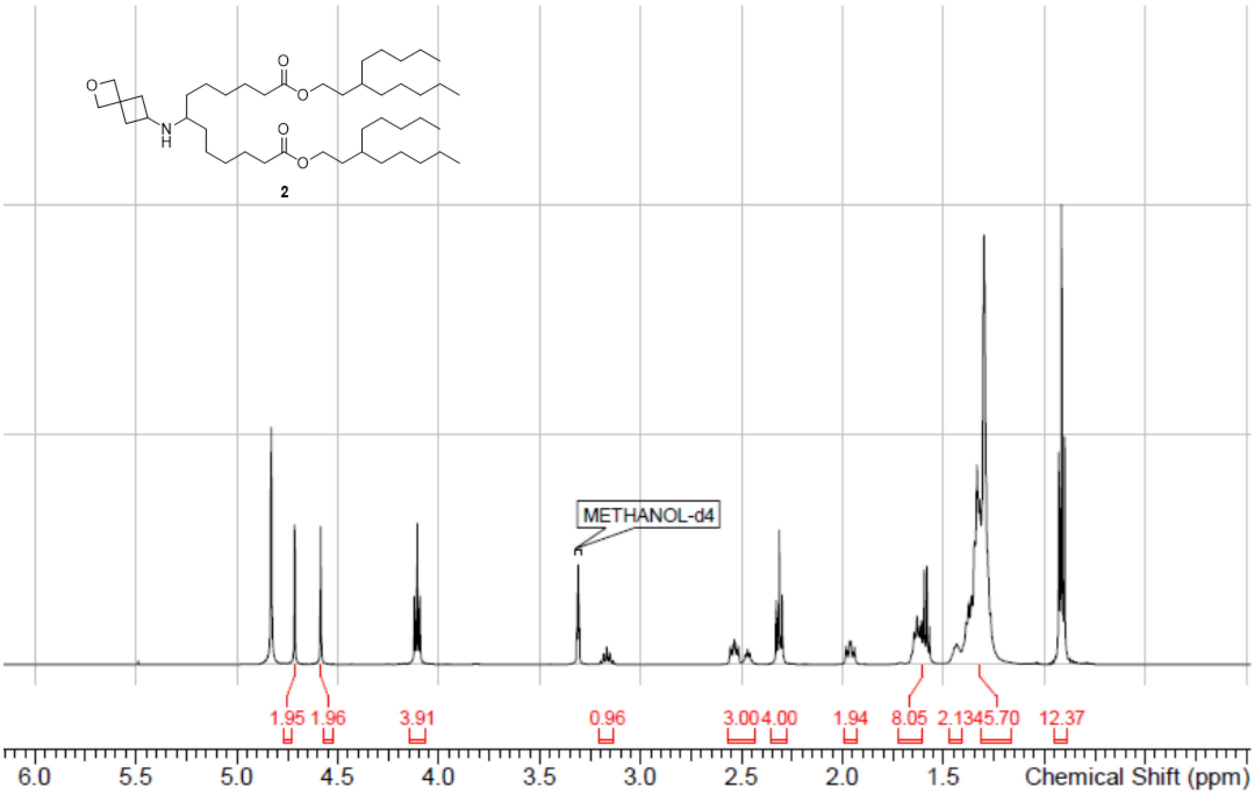

HSQC (500 MHz, Methanol-*d*4) of **2**

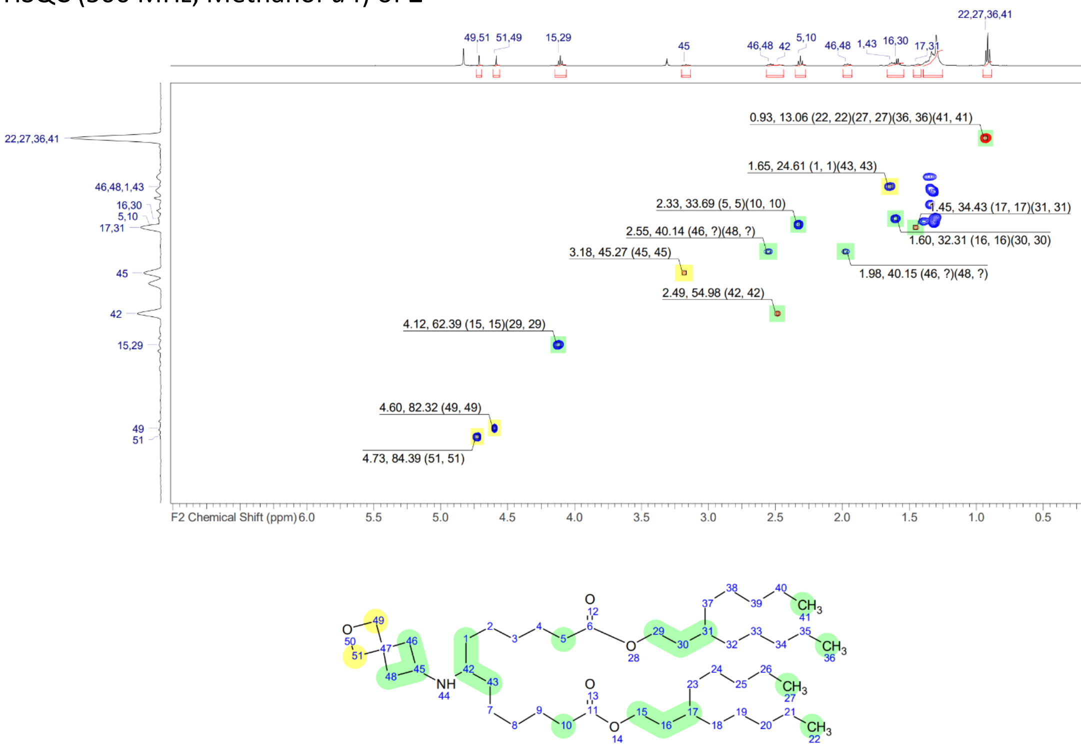

^{1}H NMR (500 MHz, Methanol-*d*4) of **3**

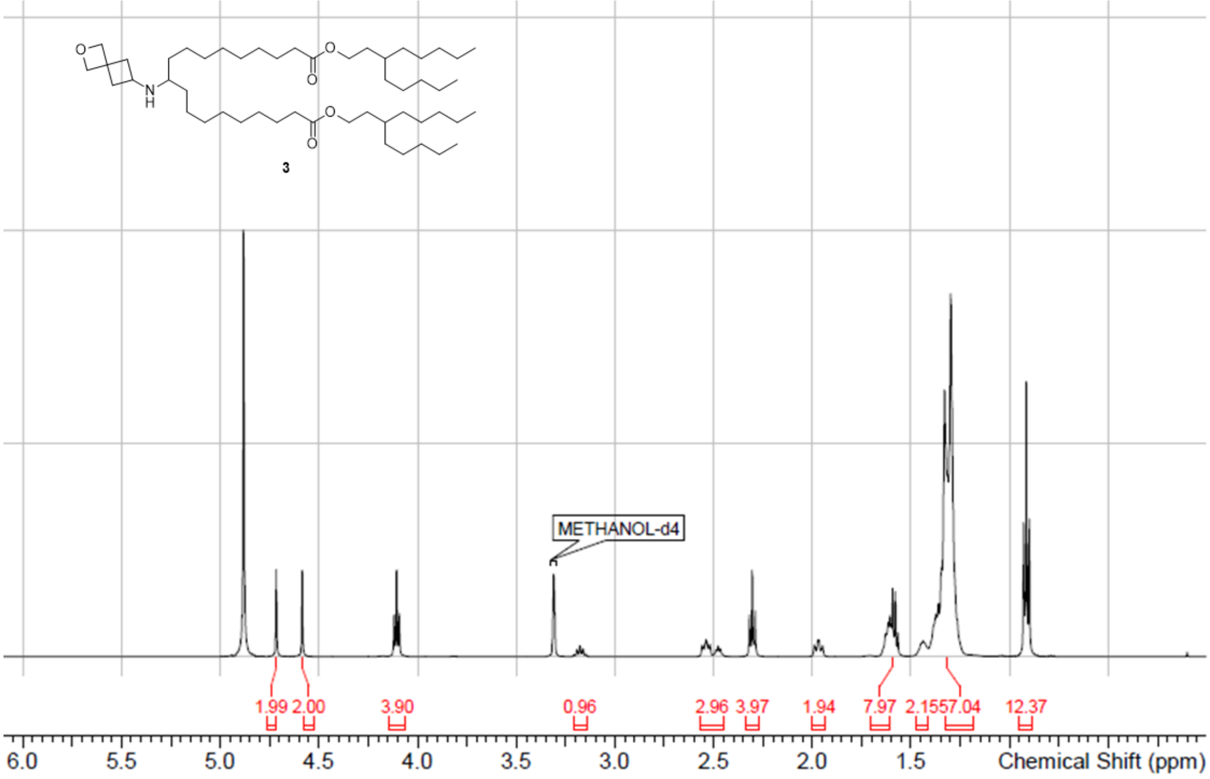

HSQC (500 MHz, Methanol-*d*4) of **3**

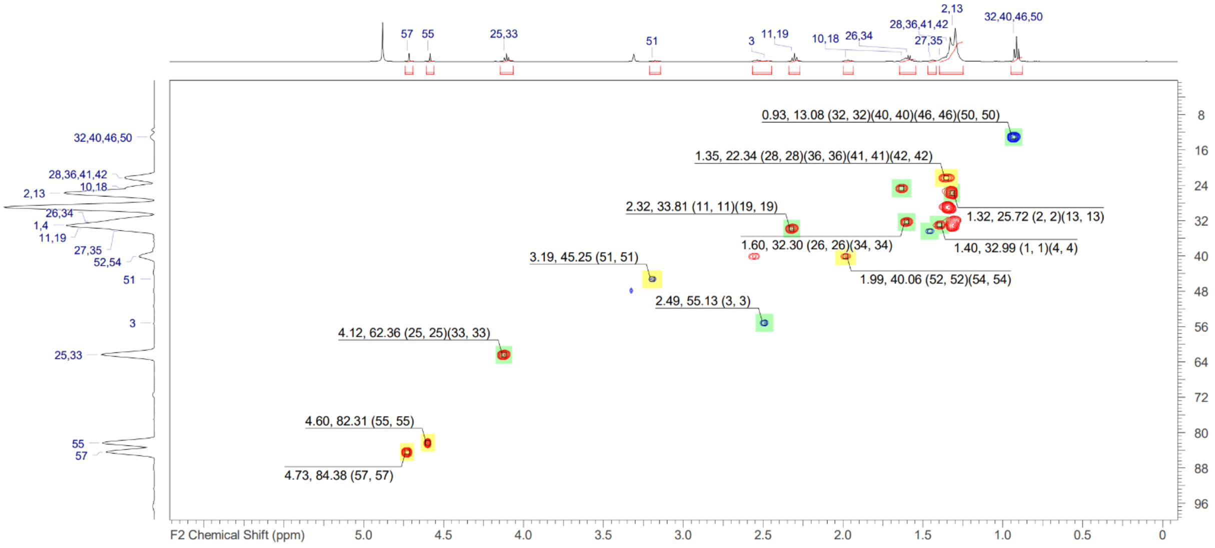

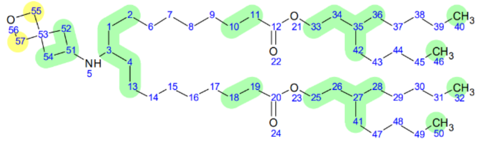

^{1}H NMR (500 MHz, Methanol-*d*4) of **4**

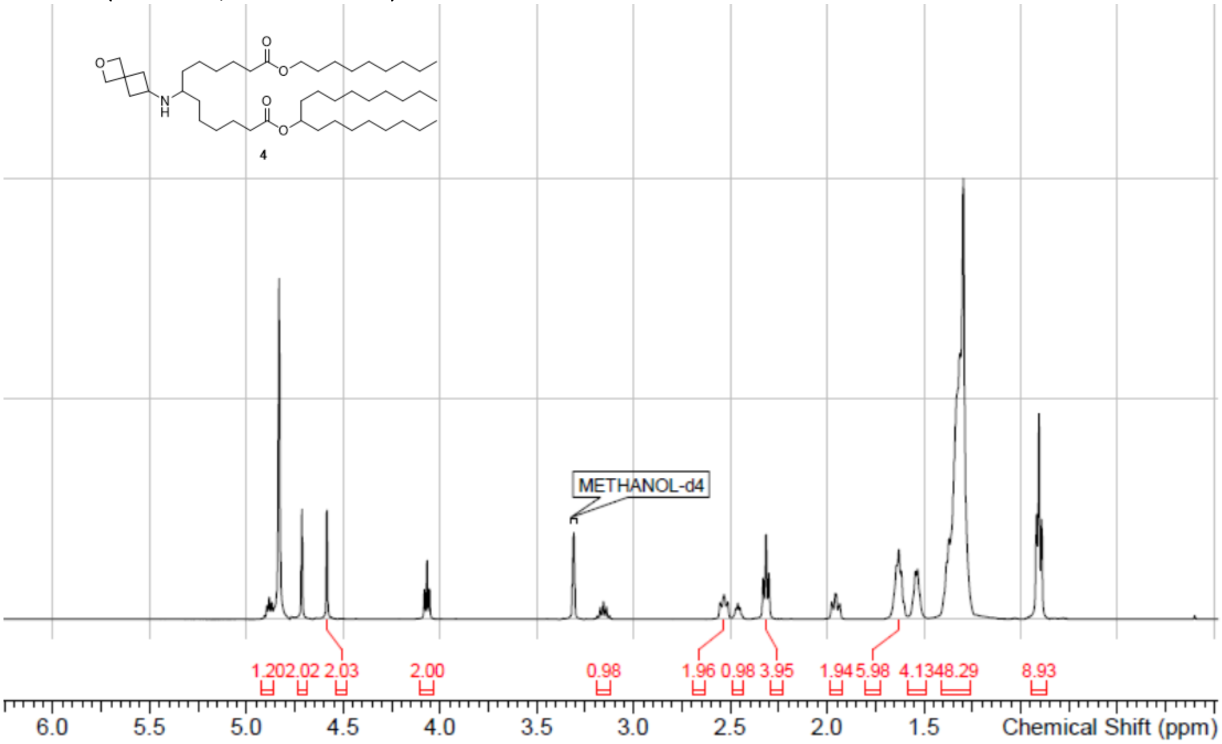

HSQC (500 MHz, Methanol-*d*4) of **4**

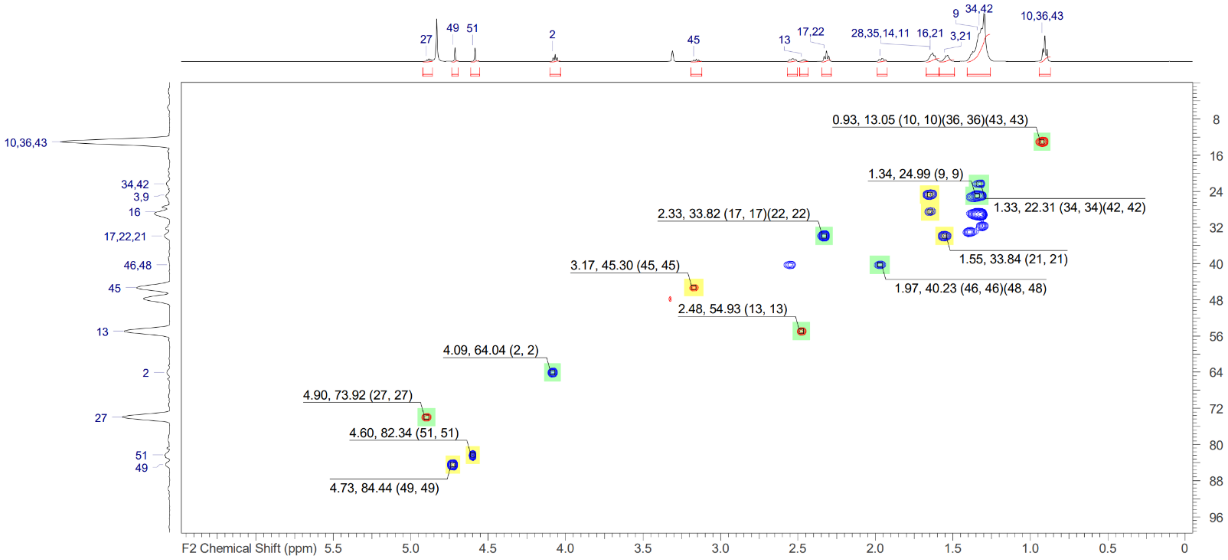

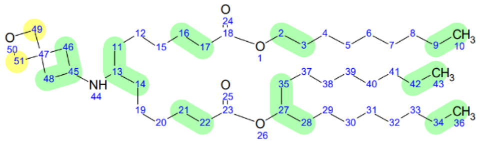

^{1}H NMR (500 MHz, $CDCl_3$) of **5**

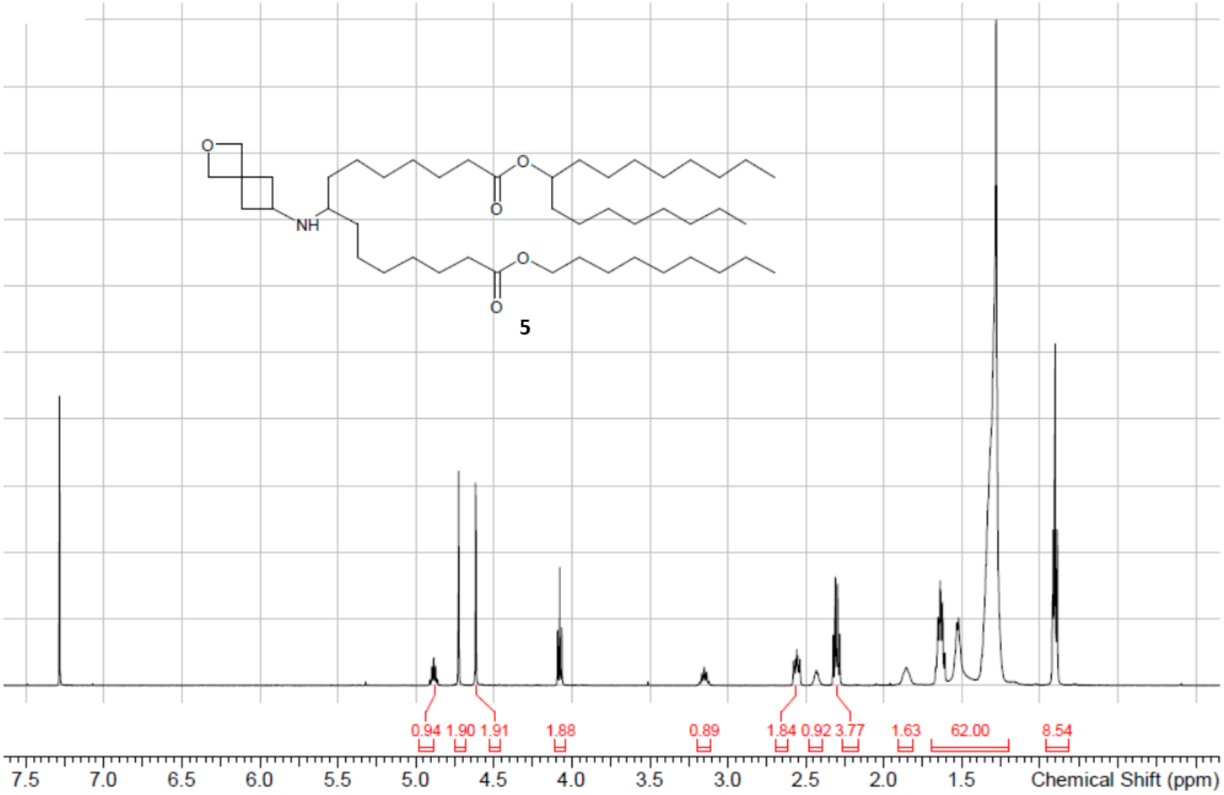

HSQC (500 MHz, $CDCl_3$) of **5**

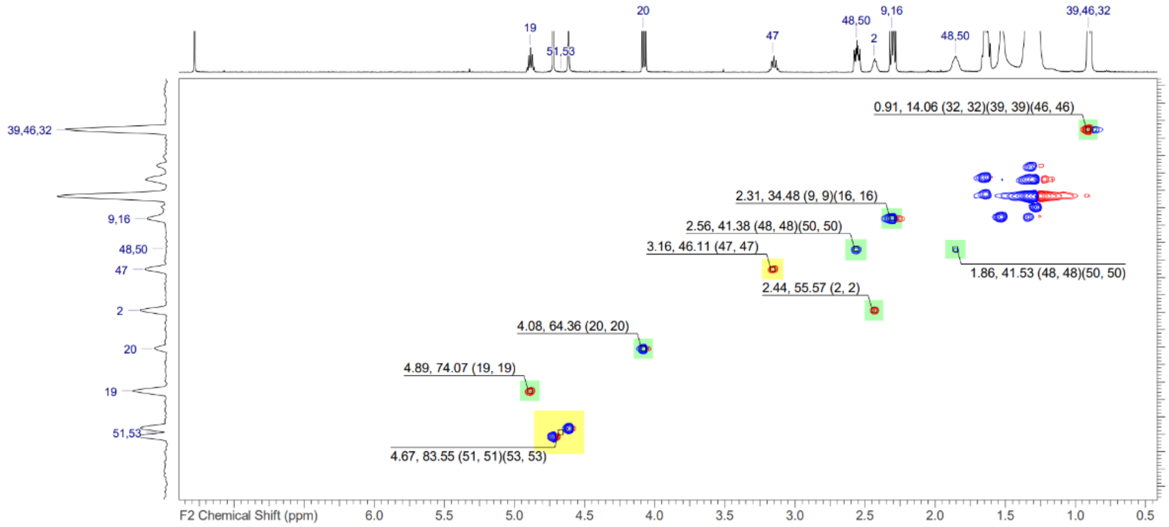

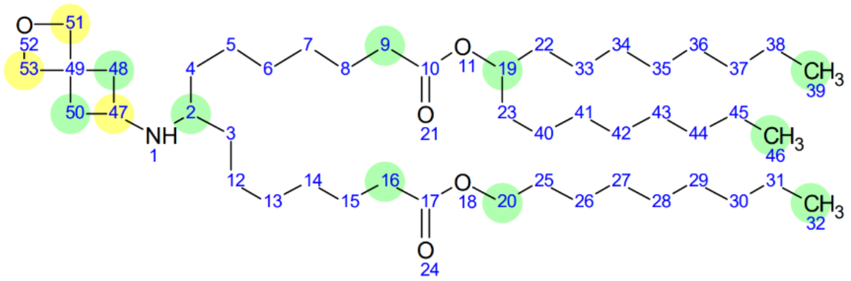

^{1}H NMR (500 MHz, Methanol-$d4$) of **6**

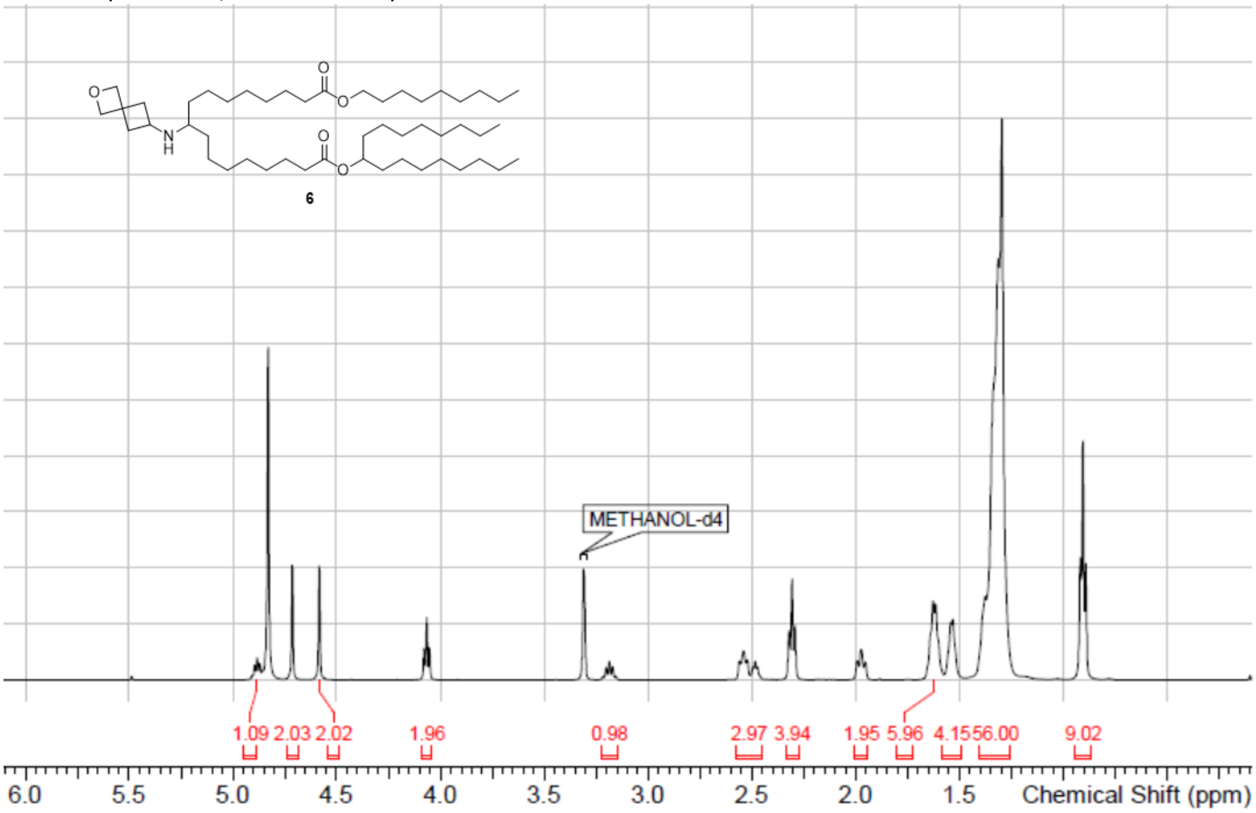

HSQC (500 MHz, Methanol-$d4$) of **6**

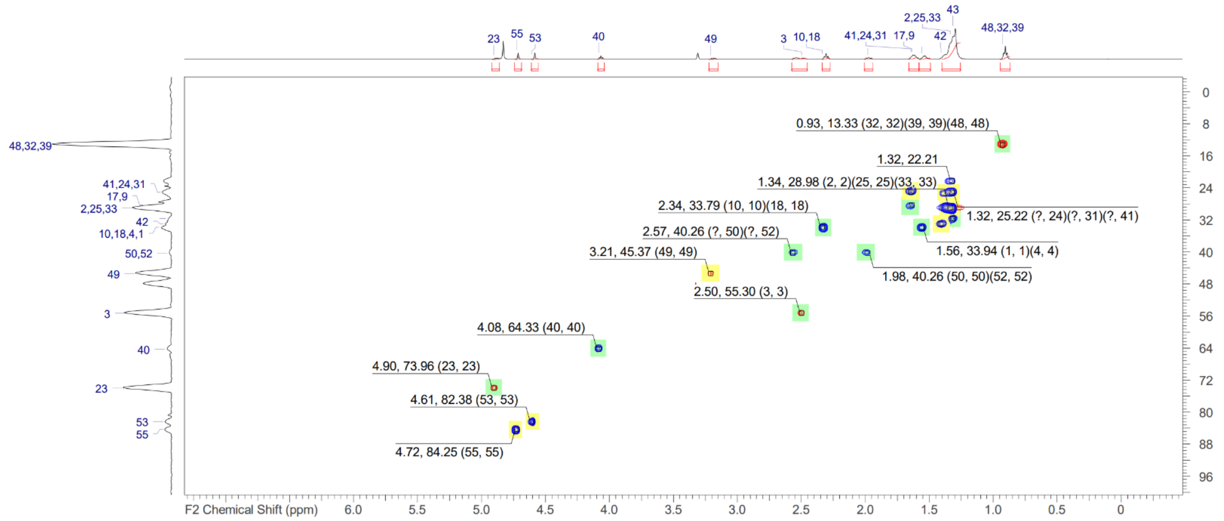

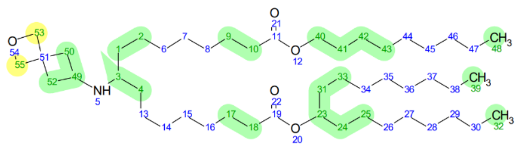

^{1}H NMR (500 MHz, Methanol-*d*4) of **7**

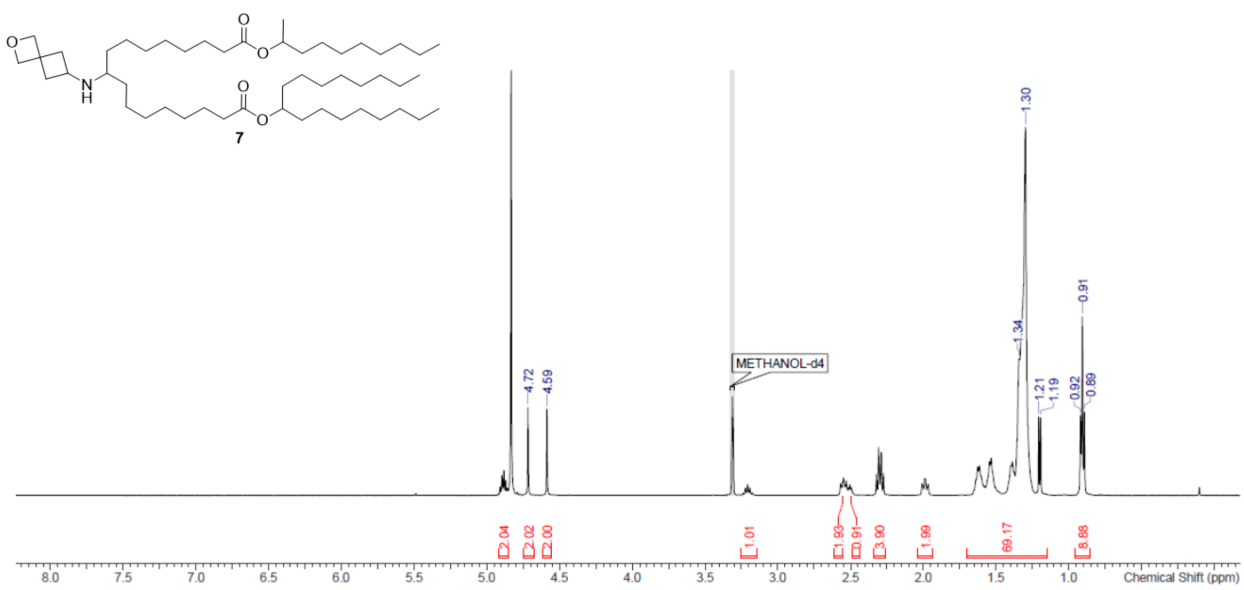

HSQC (500 MHz, Methanol-*d*4) of **7**

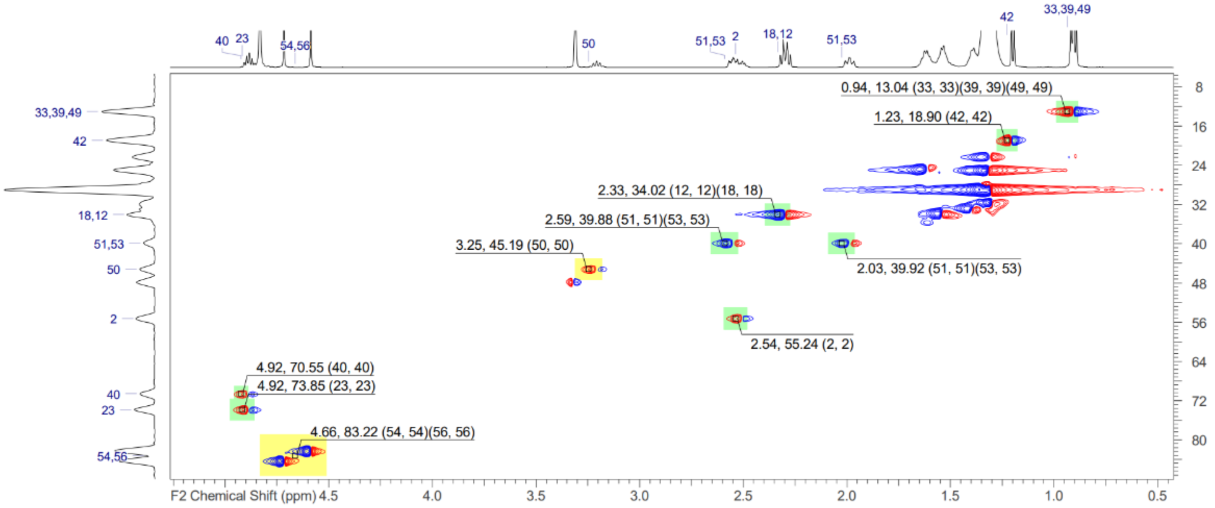

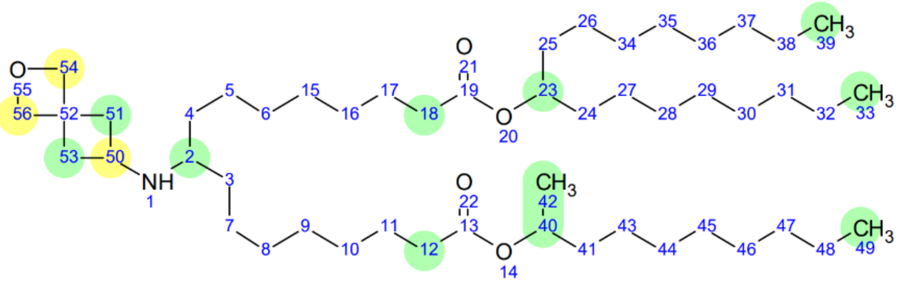

^{1}H NMR (500 MHz, Methanol-d4) of **8**

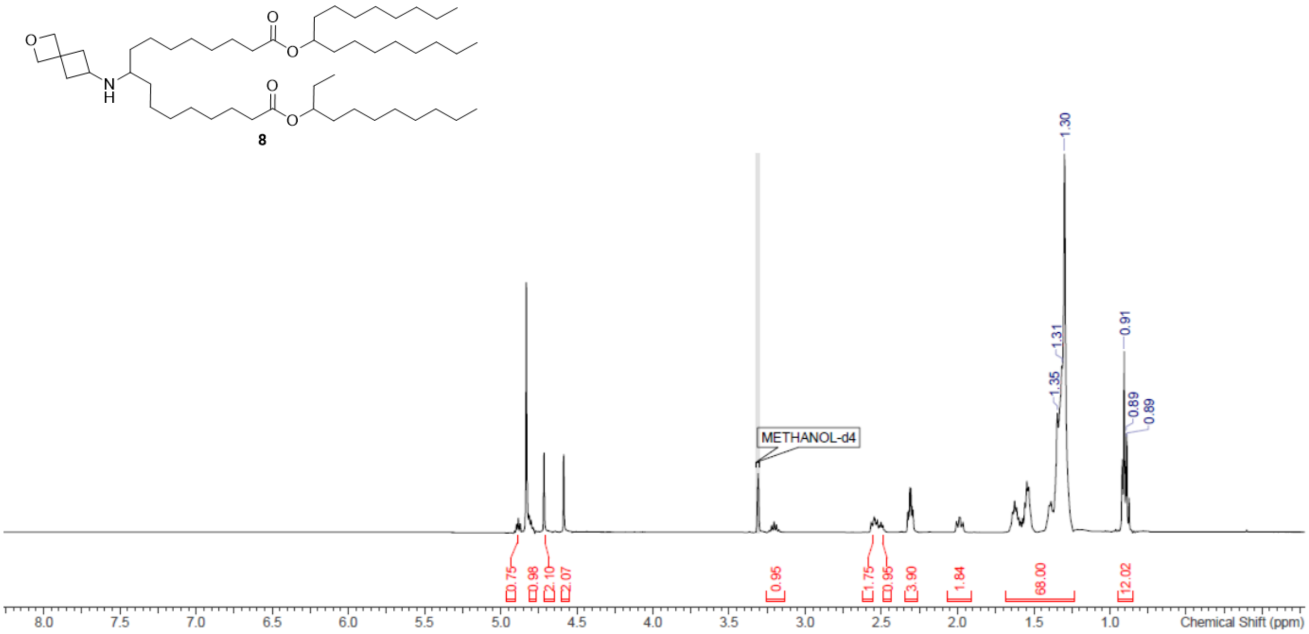

HSQC (500 MHz, Methanol-d4) of **8**

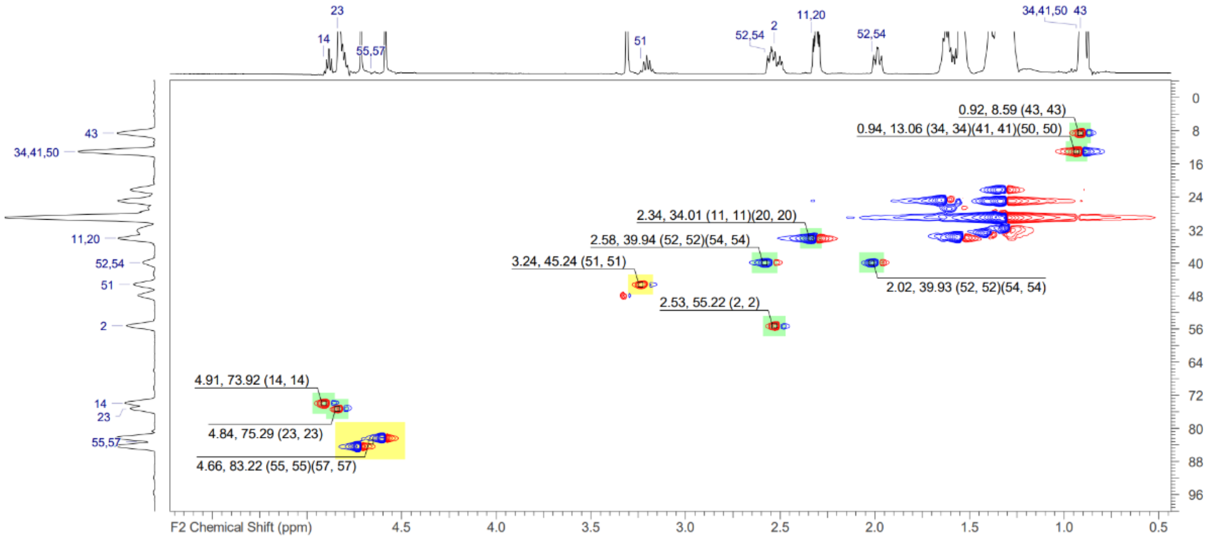

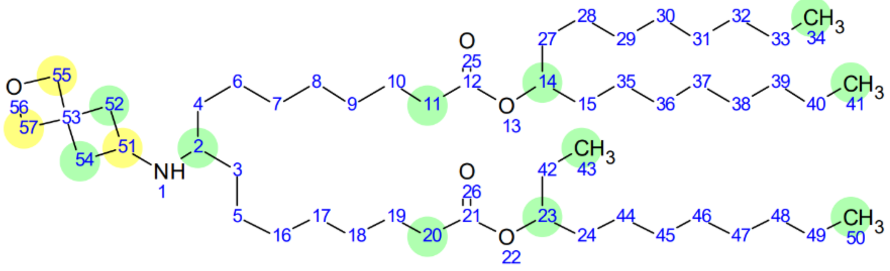

^1H NMR (500 MHz, Methanol-d4) of **9**

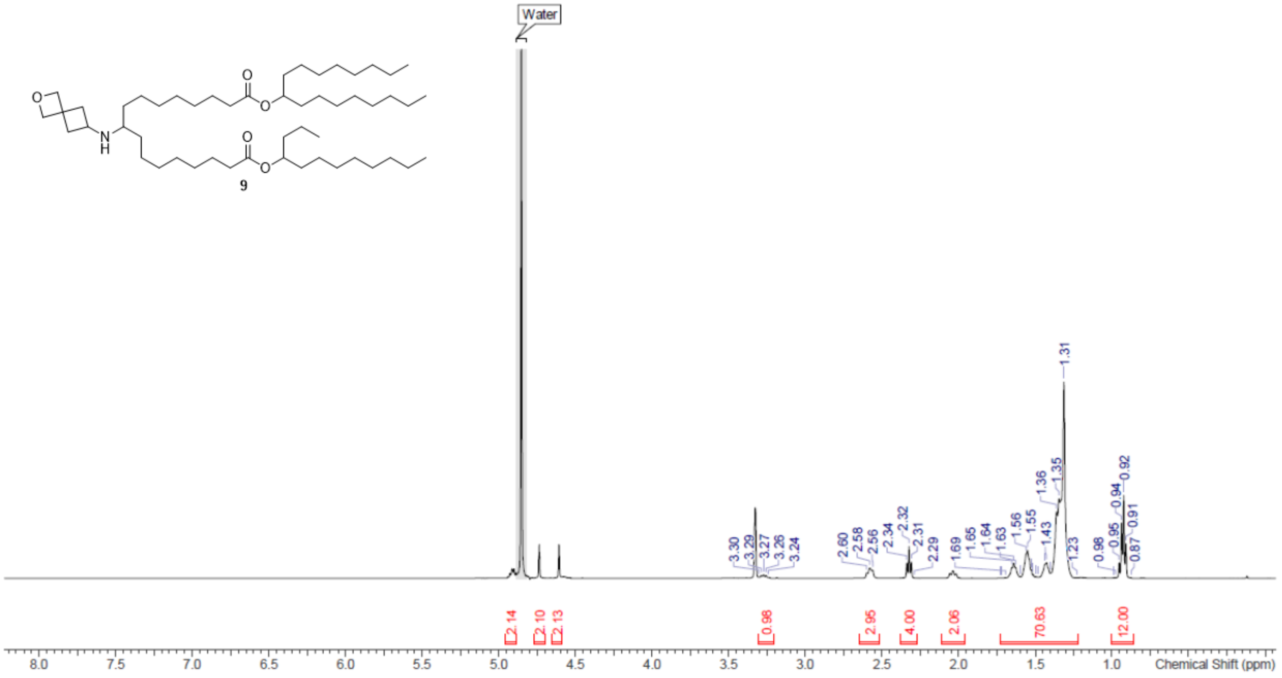

HSQC (500 MHz, Methanol-d4) of **9**

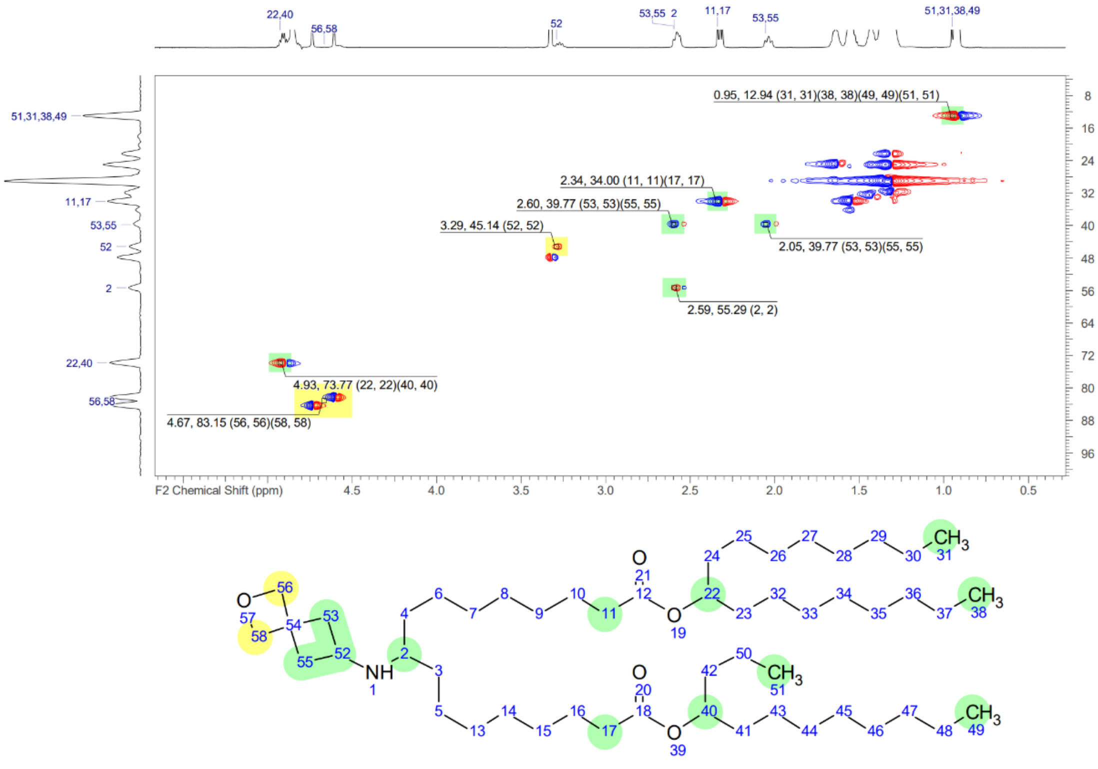

^{1}H NMR (500 MHz, Methanol-d4) of **10**

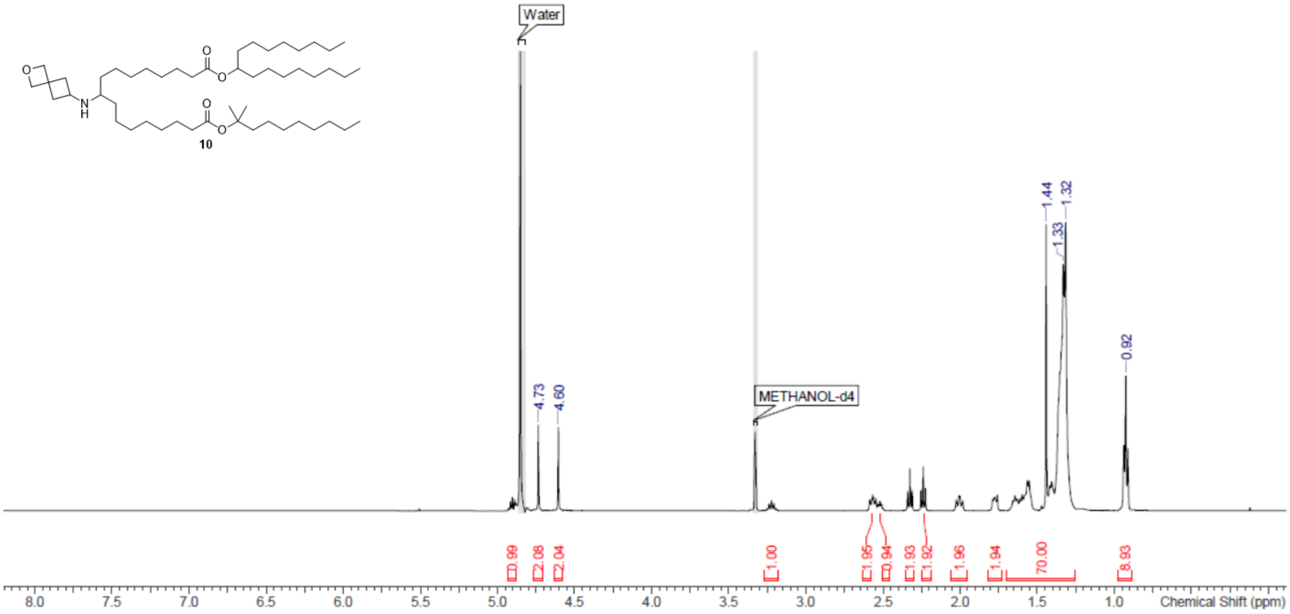

HSQC (500 MHz, Methanol-d4) of **10**

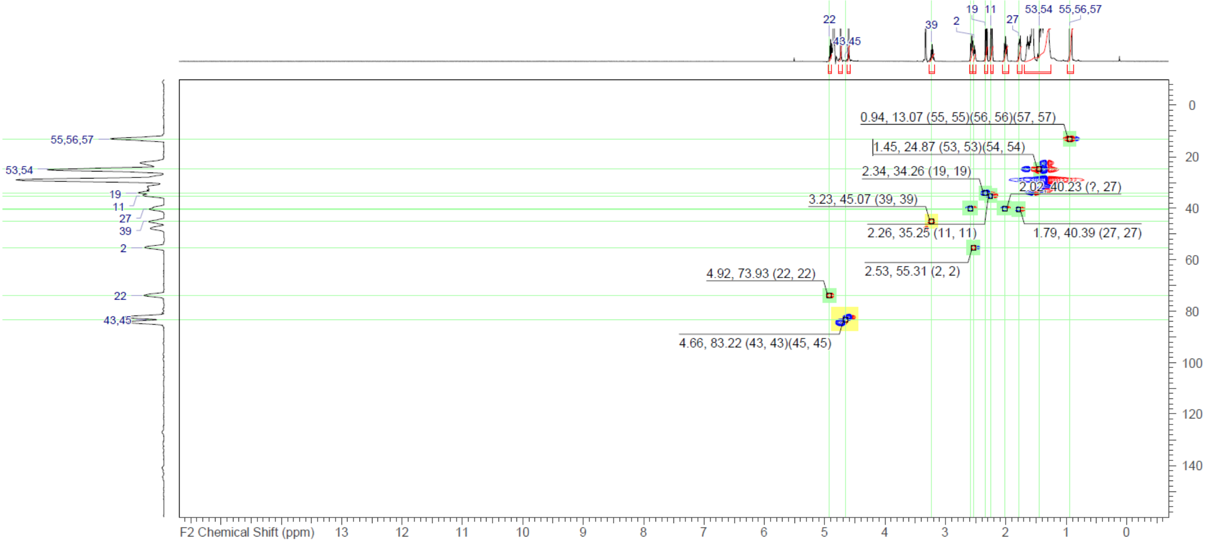

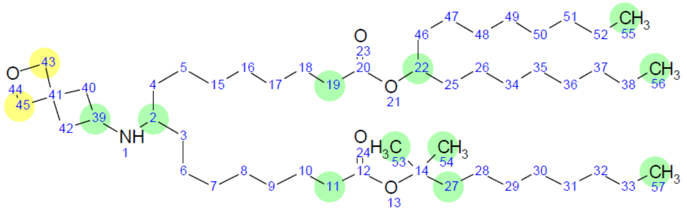

^{1}H NMR (500 MHz, Methanol-d4) of **11**

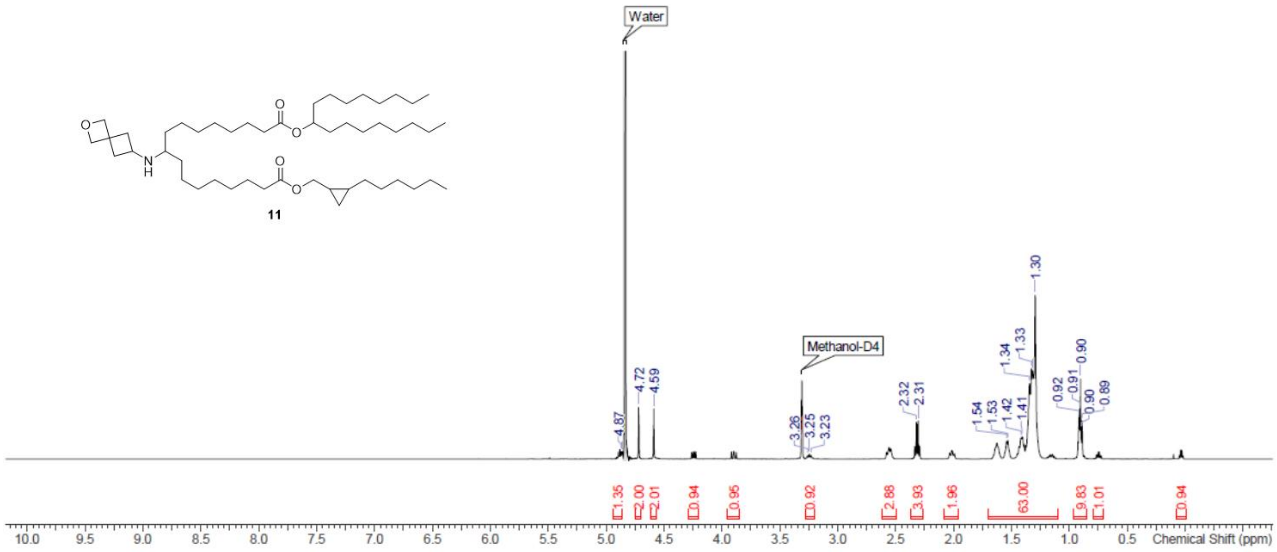

HSQC (500 MHz, Methanol-d4) of **11**

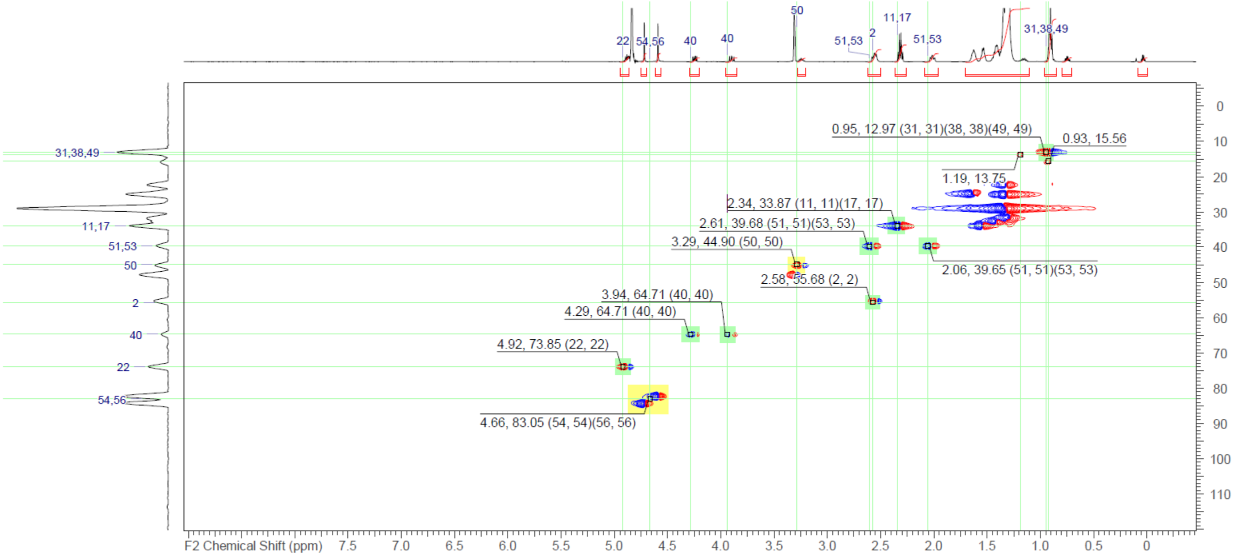

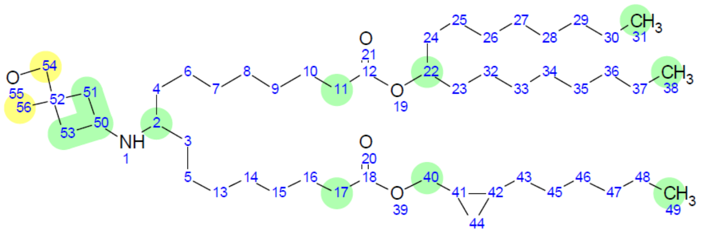

^{1}H NMR (400 MHz, Methanol-d4) of **12**

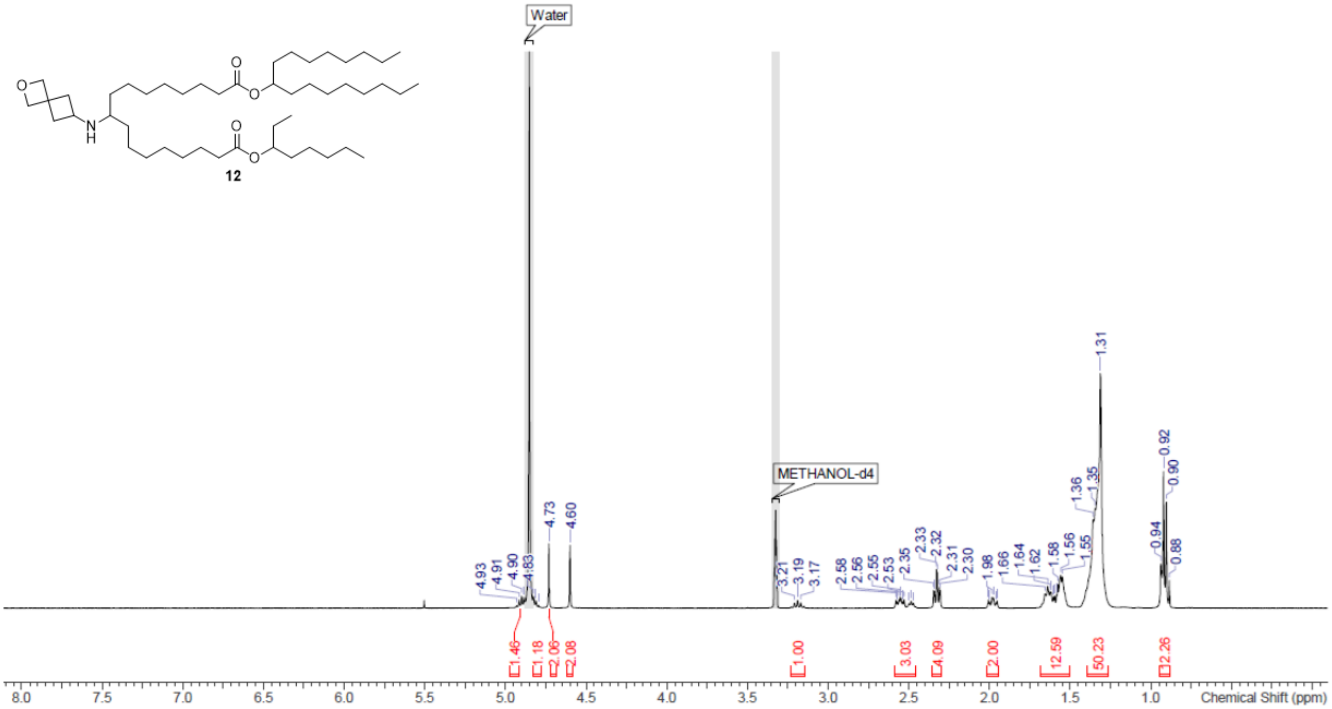

HSQC (500 MHz, Methanol-*d*4) of **12**

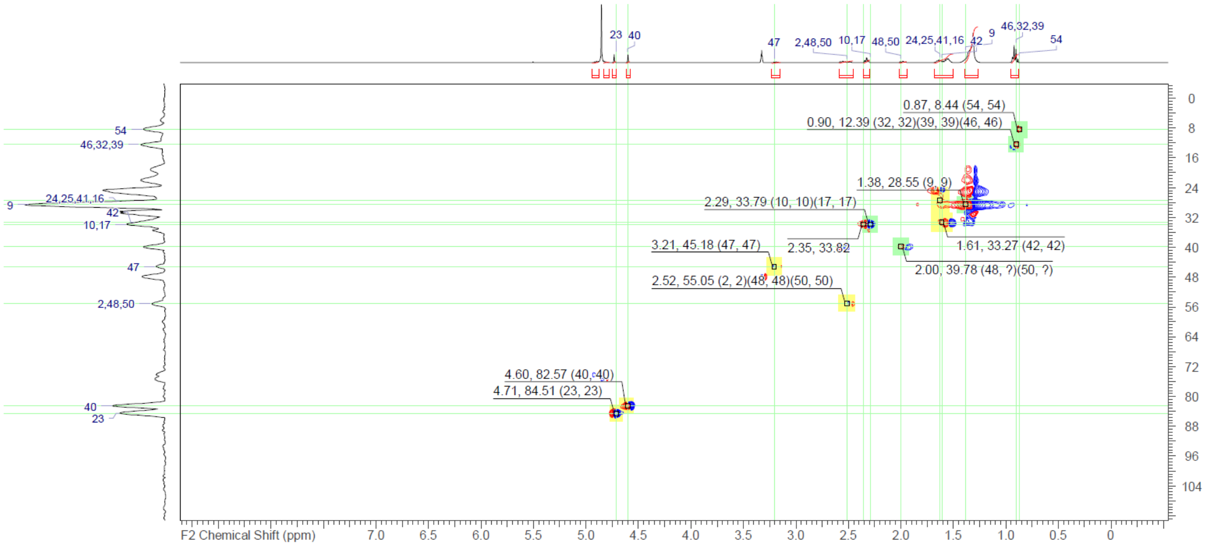

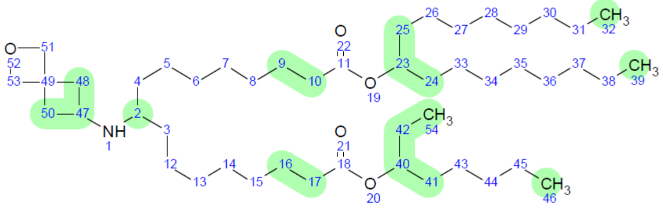

^{1}H NMR (500 MHz, Methanol-*d*4) of **13**

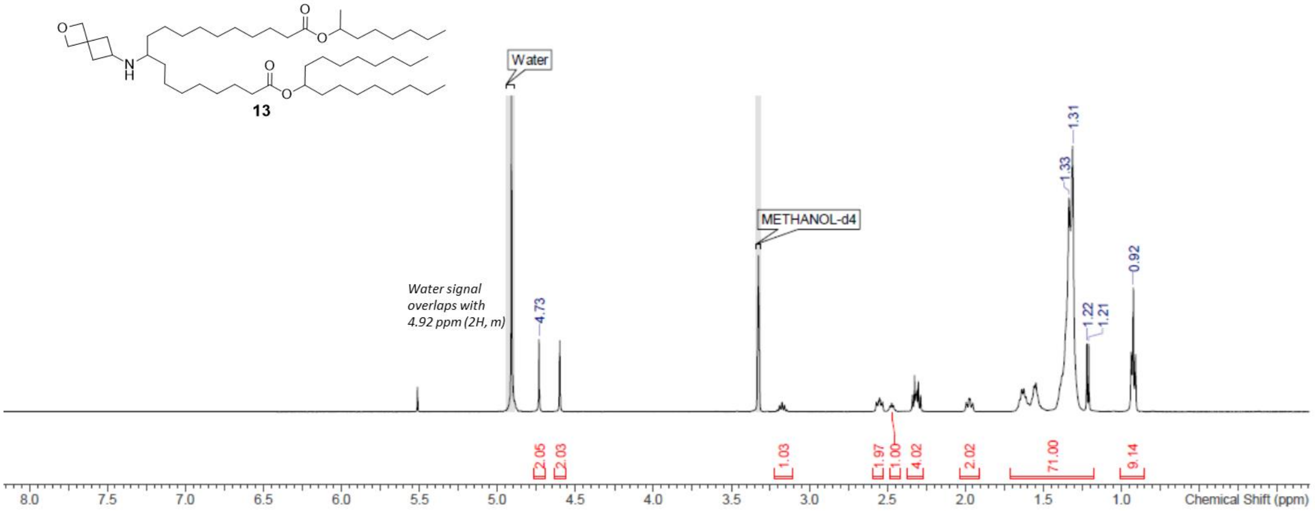

HSQC (500 MHz, Methanol-*d*4) of **13**

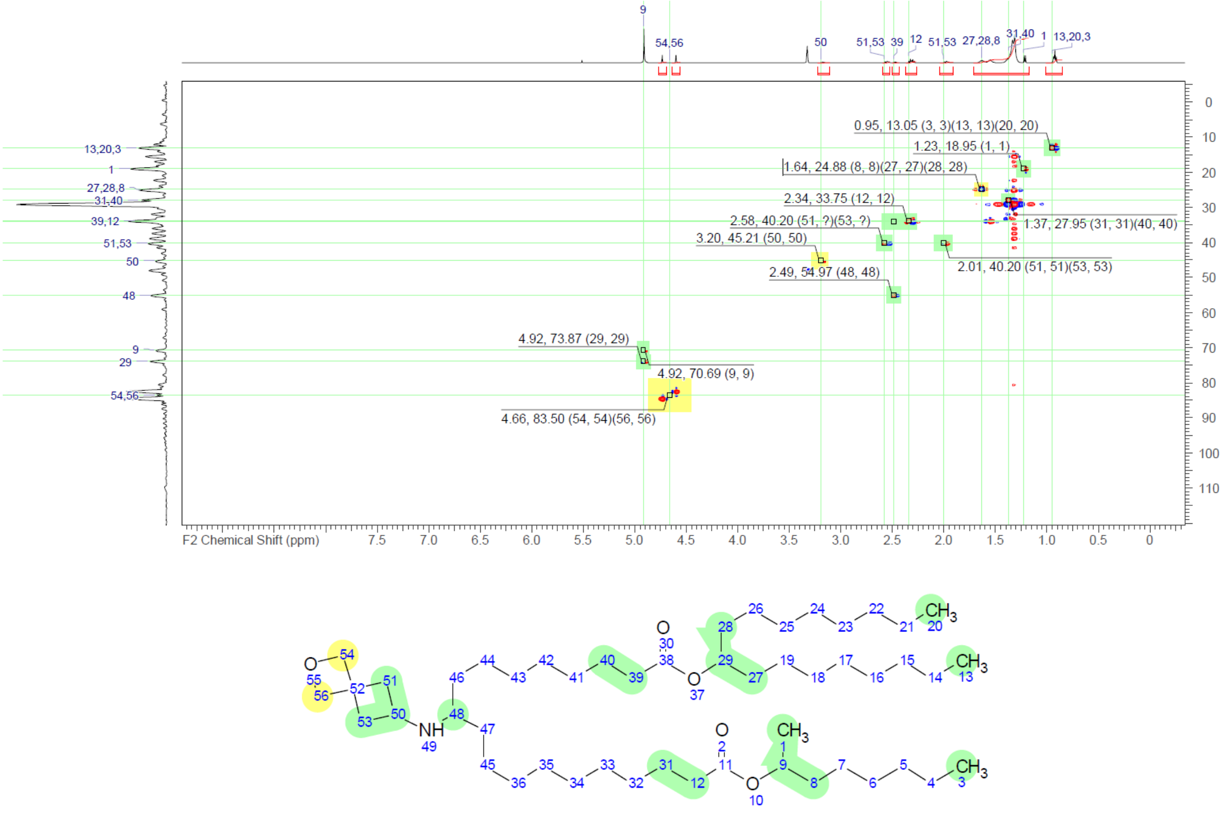

^{1}H NMR (500 MHz, Methanol-*d*4) of **14**

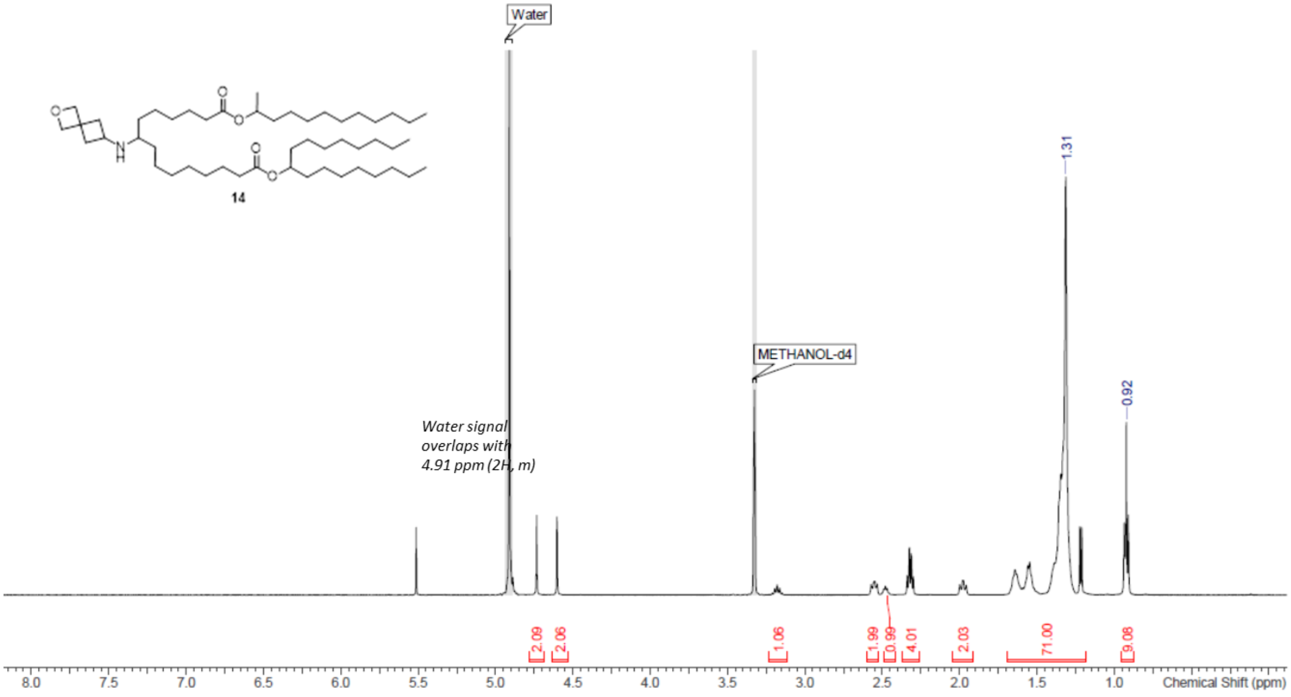

HSQC (500 MHz, Methanol-*d*4) of **14**

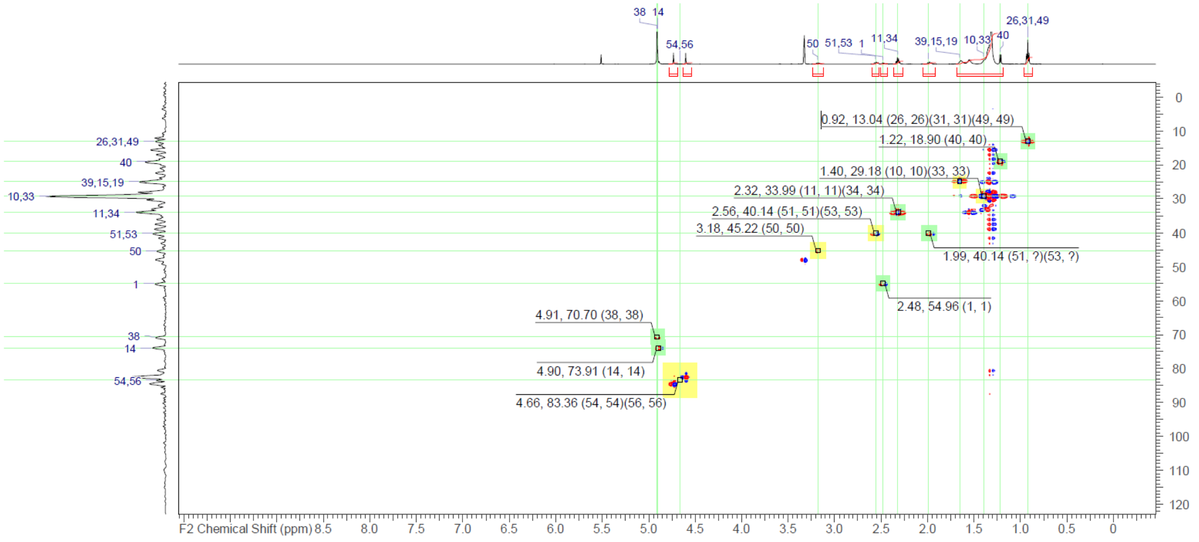

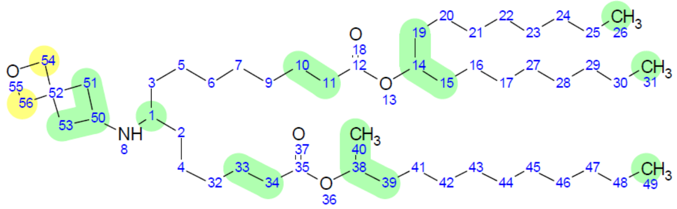

^{1}H NMR (500 MHz, Methanol-*d*4) of **15**

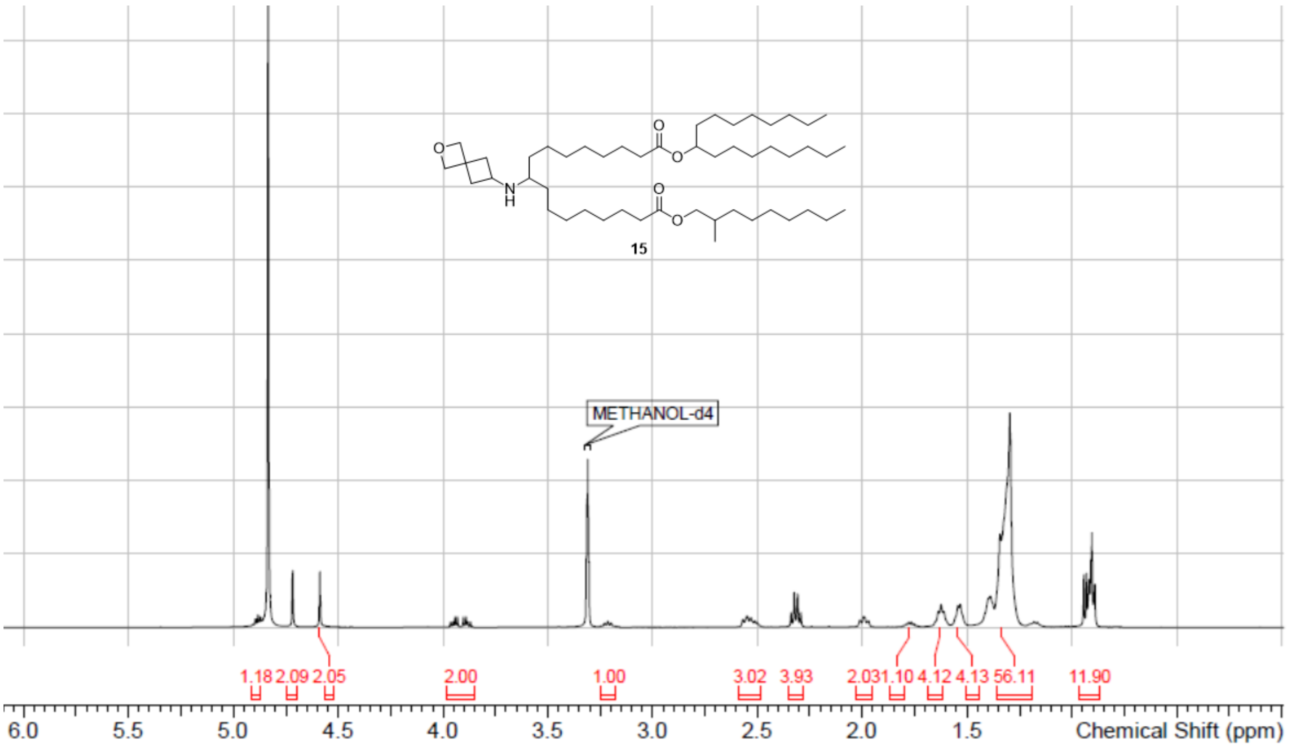

HSQC (500 MHz, Methanol-*d*4) of **15**

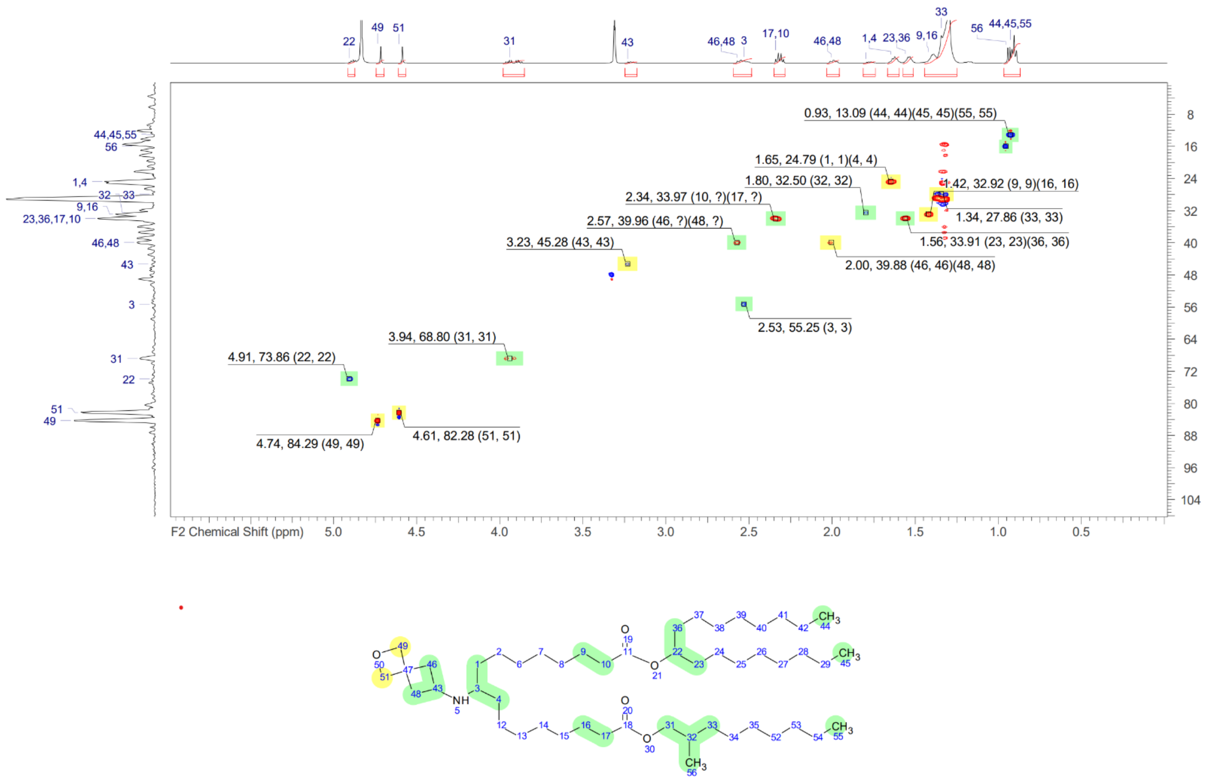

^{1}H NMR (500 MHz, Methanol-*d*4) of **16**

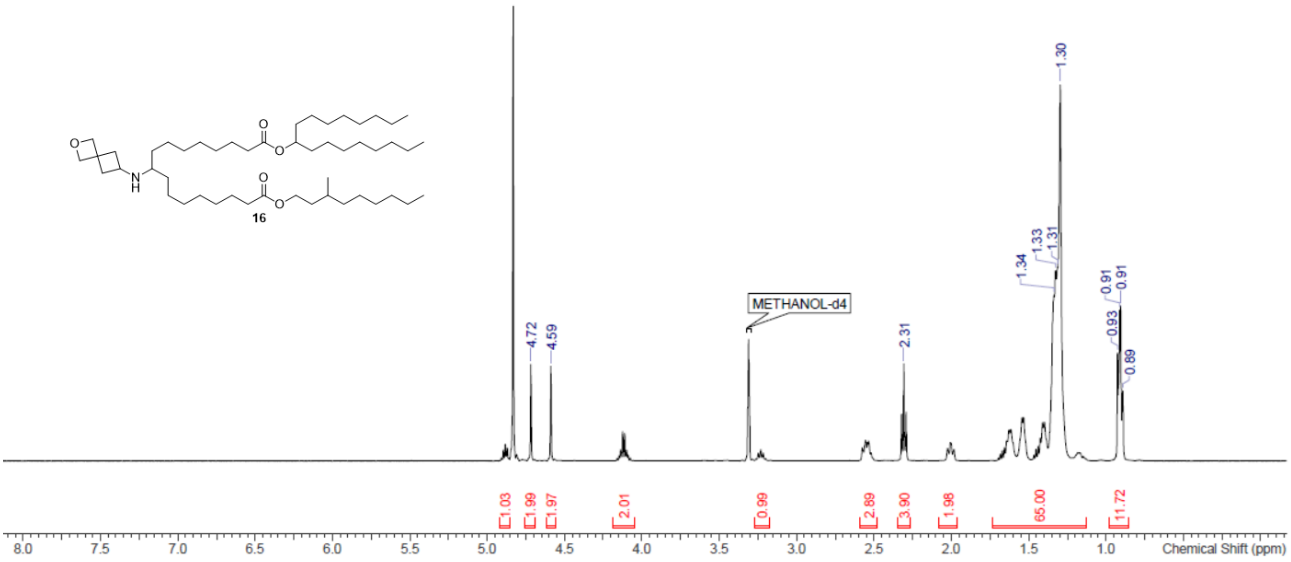

HSQC (500 MHz, Methanol-*d*4) of **16**

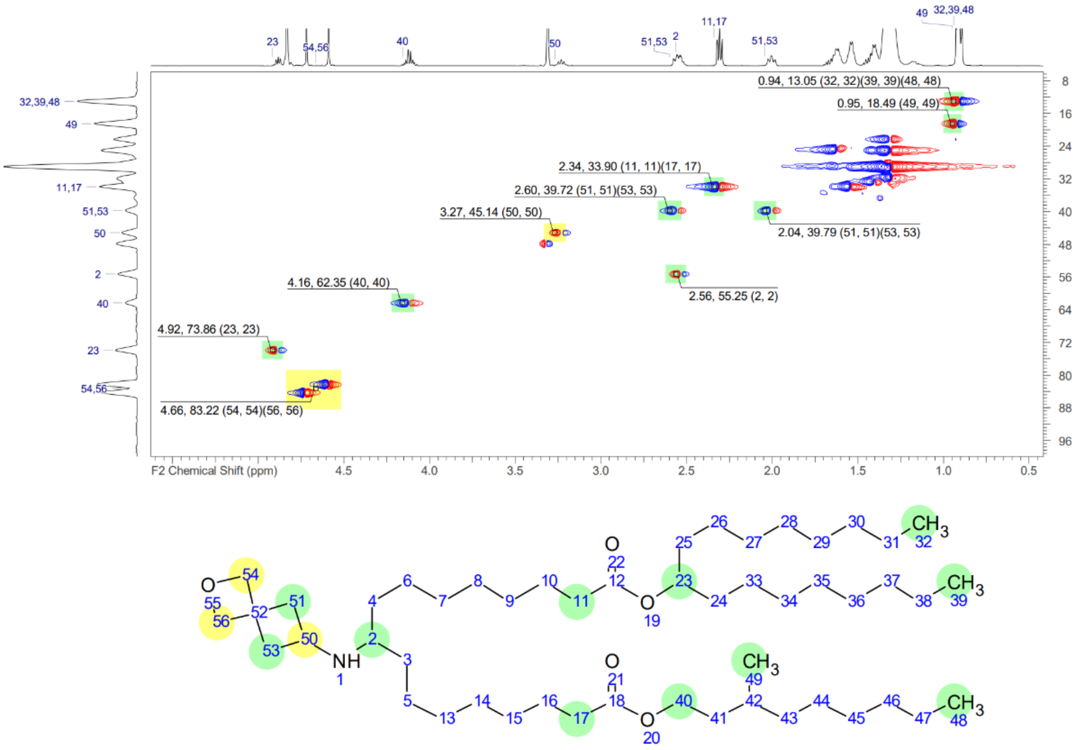

^{1}H NMR (500 MHz, Methanol-*d*4) of **17**

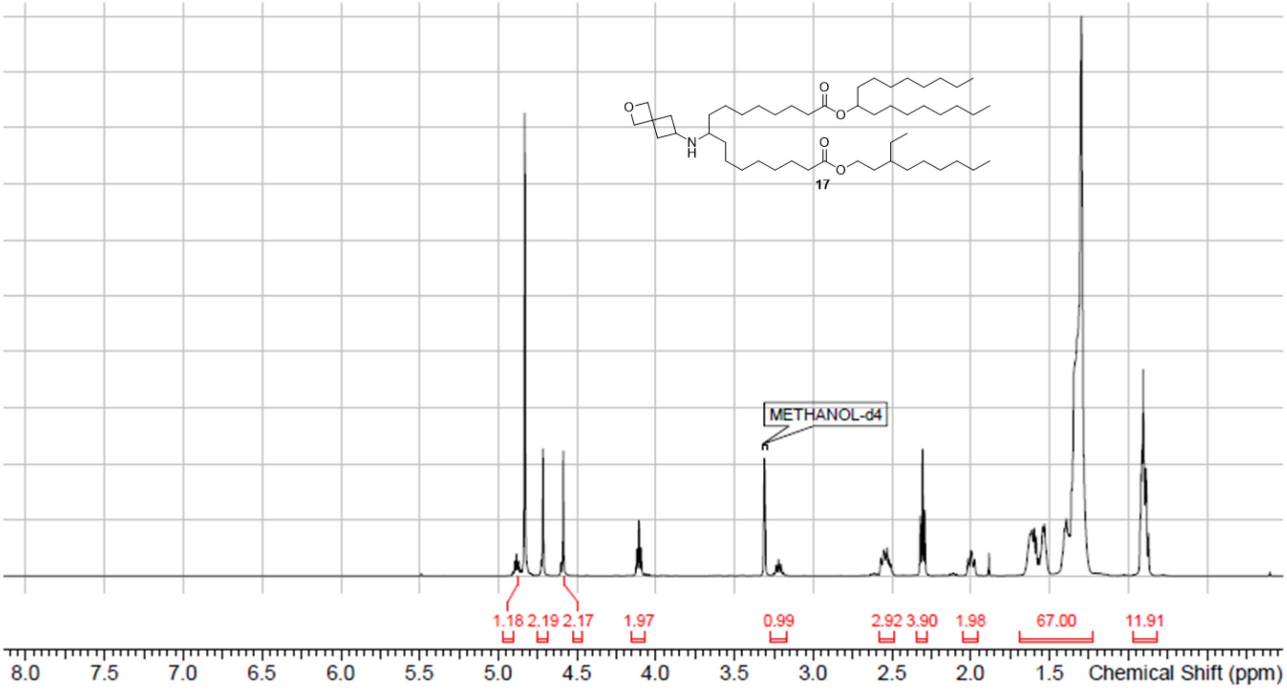

HSQC (500 MHz, Methanol-*d*4) of **17**

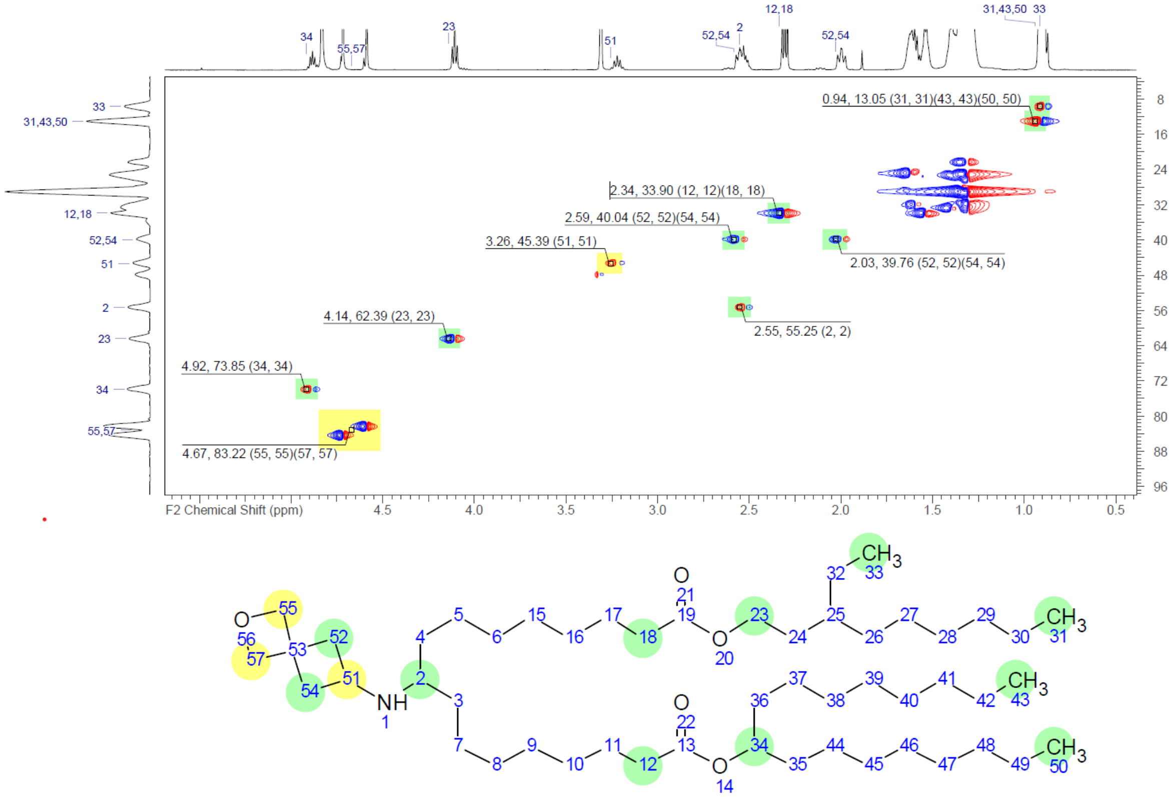

^{1}H NMR (500 MHz, Methanol-*d*4) of **18**

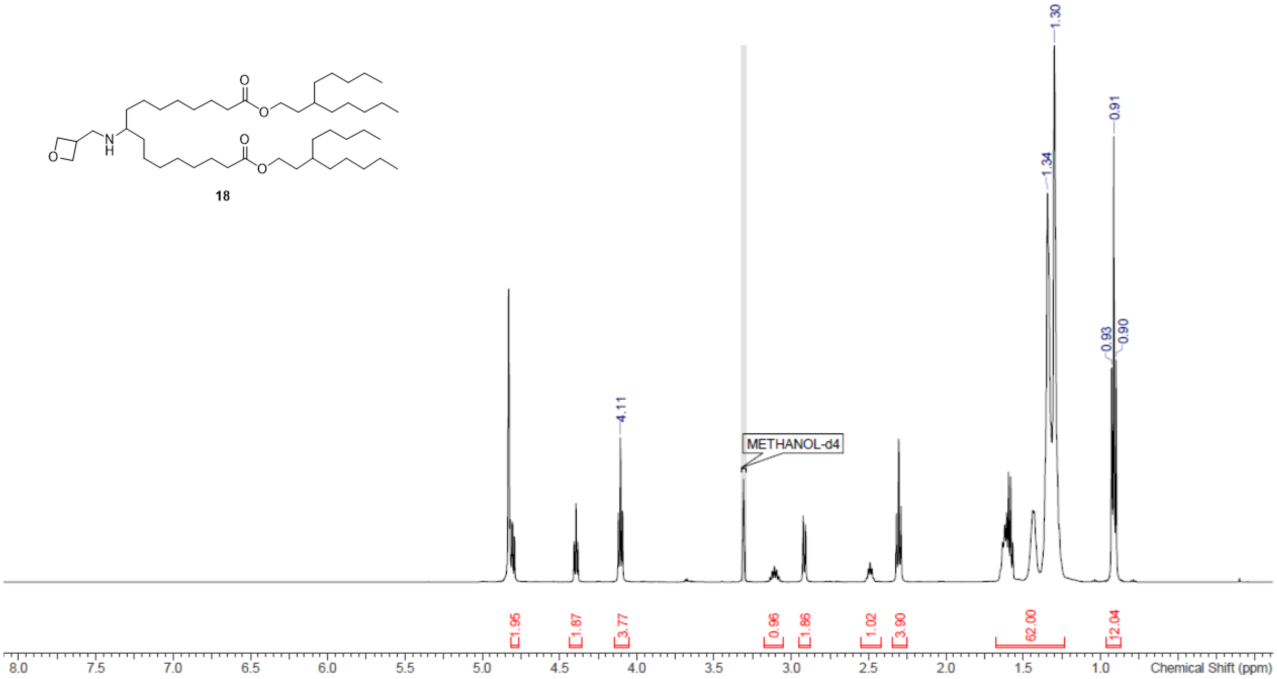

HSQC (500 MHz, Methanol-*d*4) of **18**

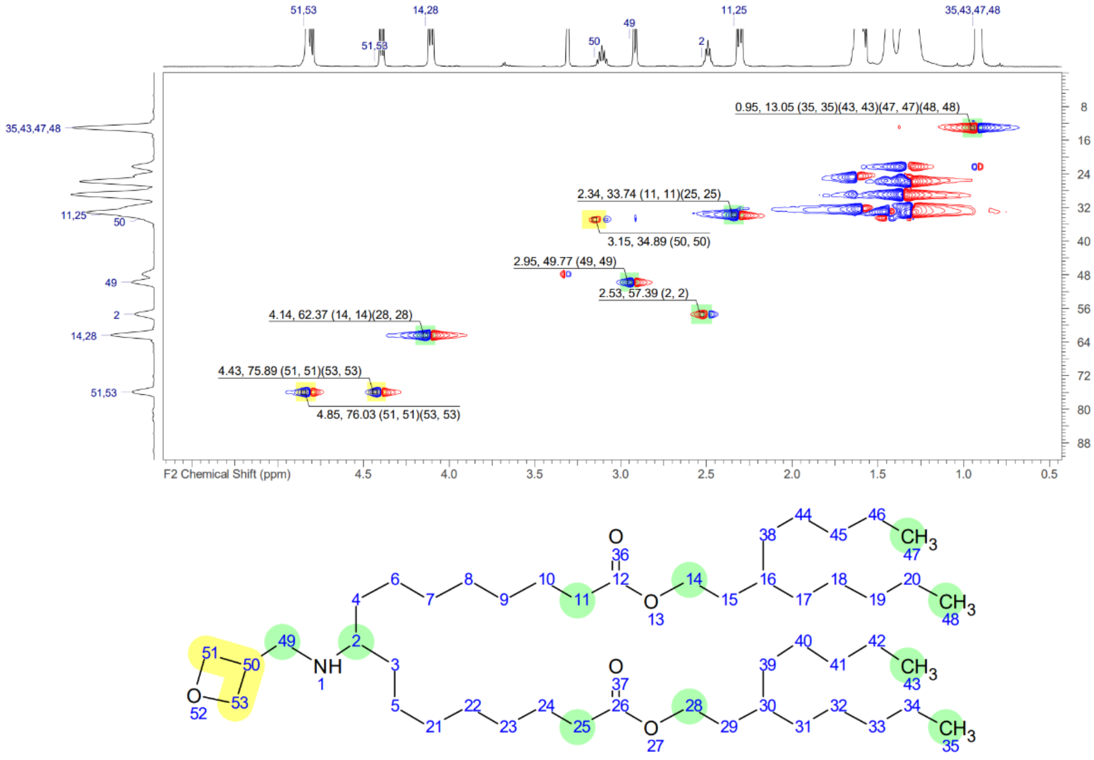

^{1}H NMR (500 MHz, Methanol-*d*4) of **19**

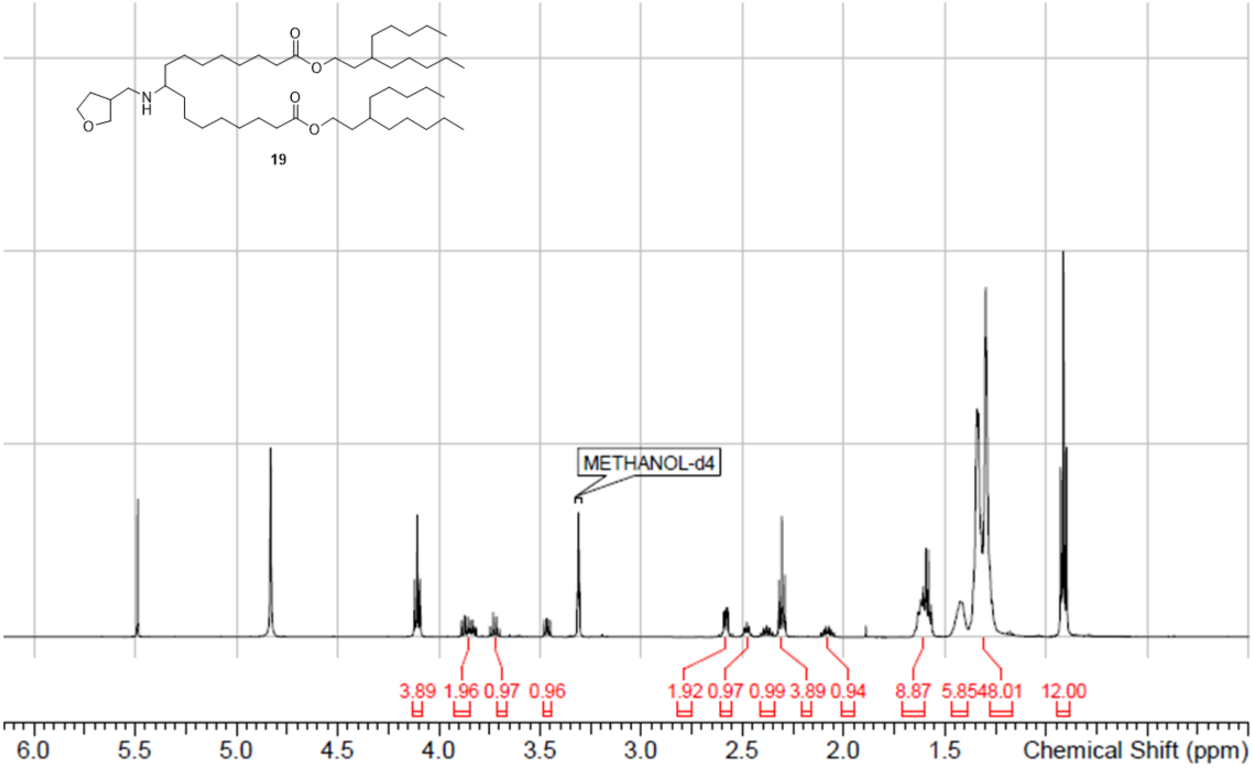

HSQC (500 MHz, Methanol-*d*4) of **19**

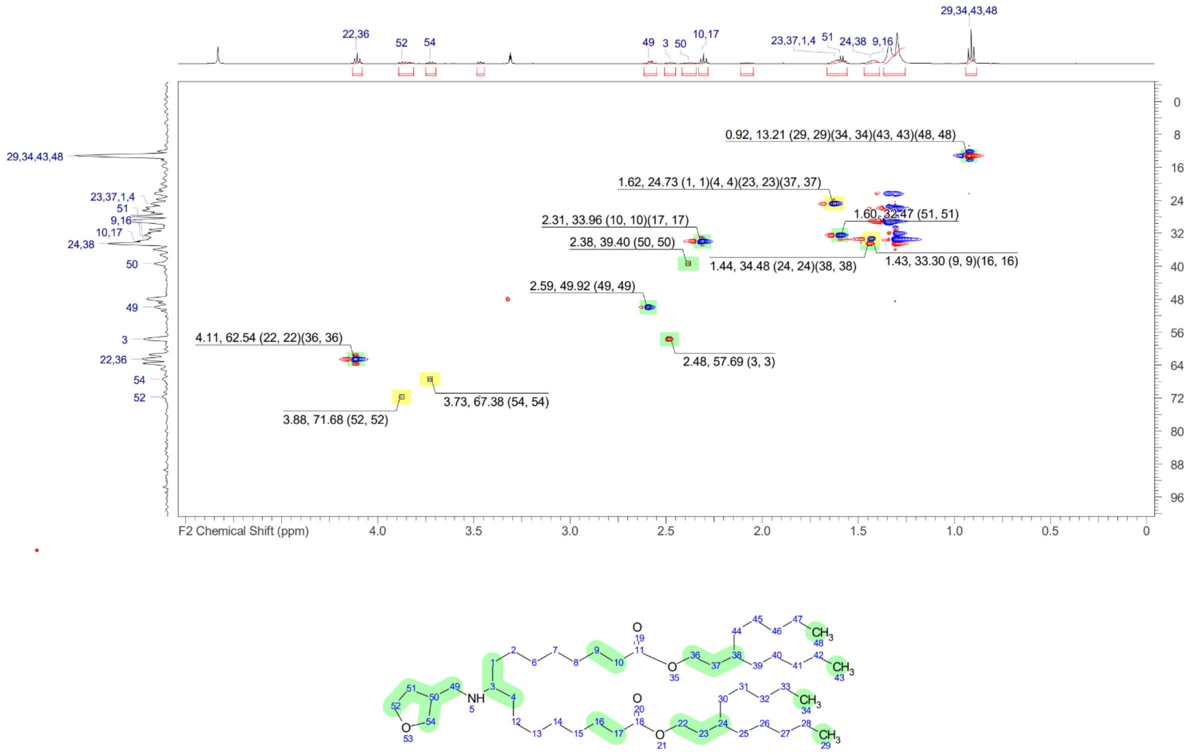

^{1}H NMR (500 MHz, Methanol-*d*4) of **20**

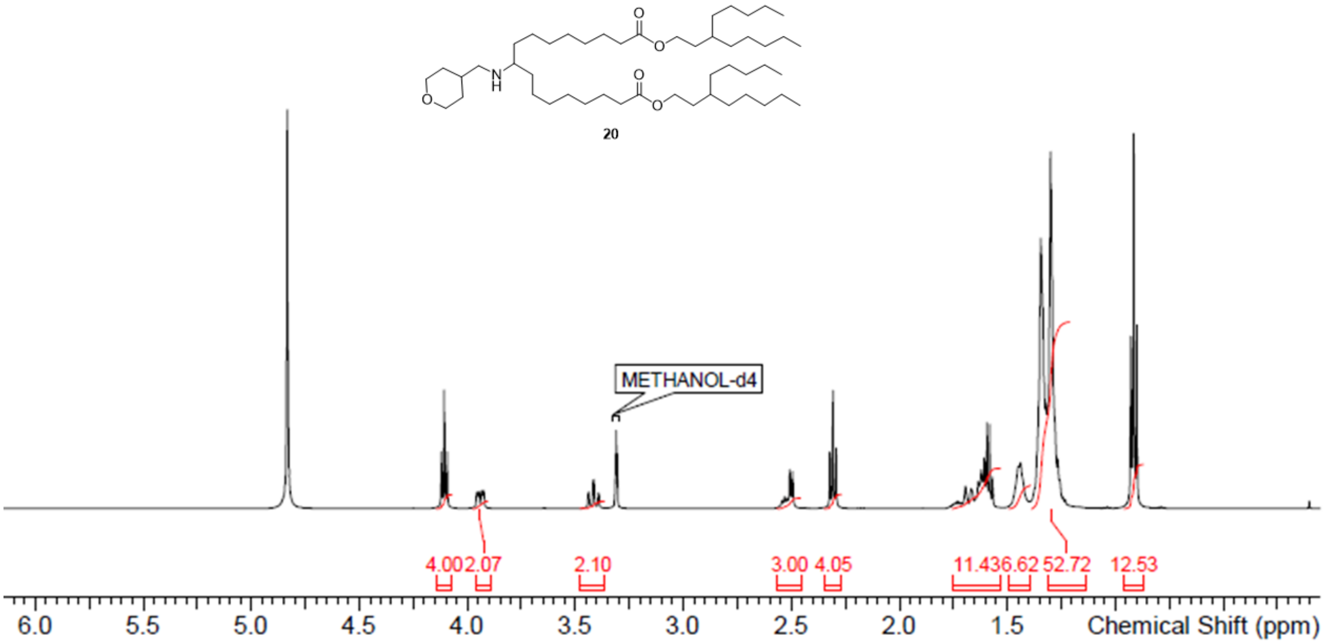

HSQC (500 MHz, Methanol-*d*4) of **20**

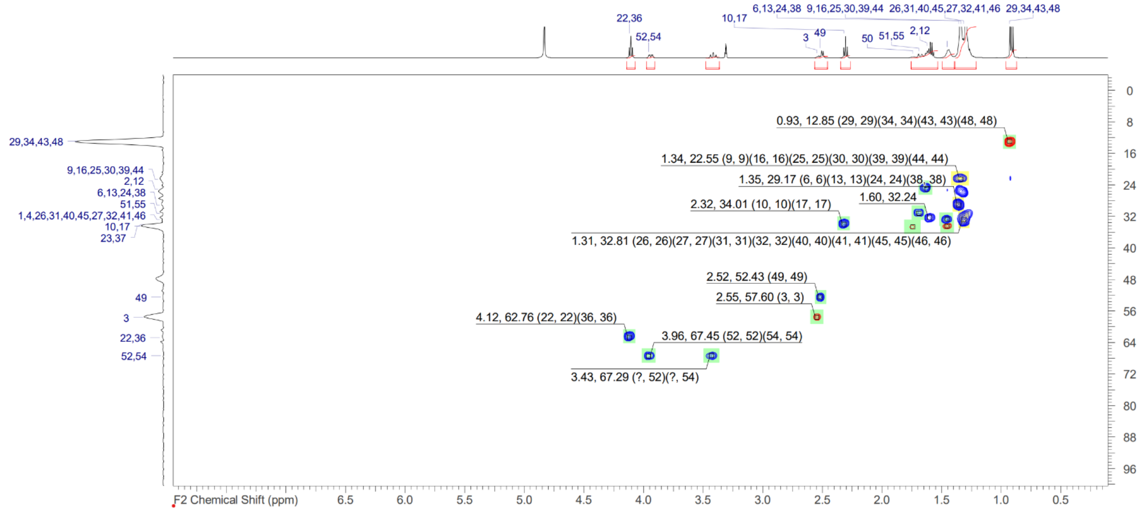

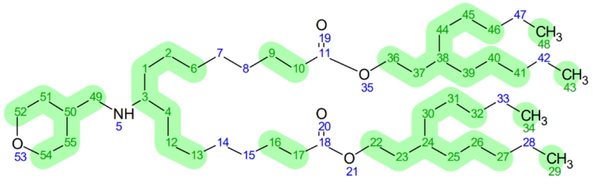

^{1}H NMR (500 MHz, Methanol-*d*4) of **21**

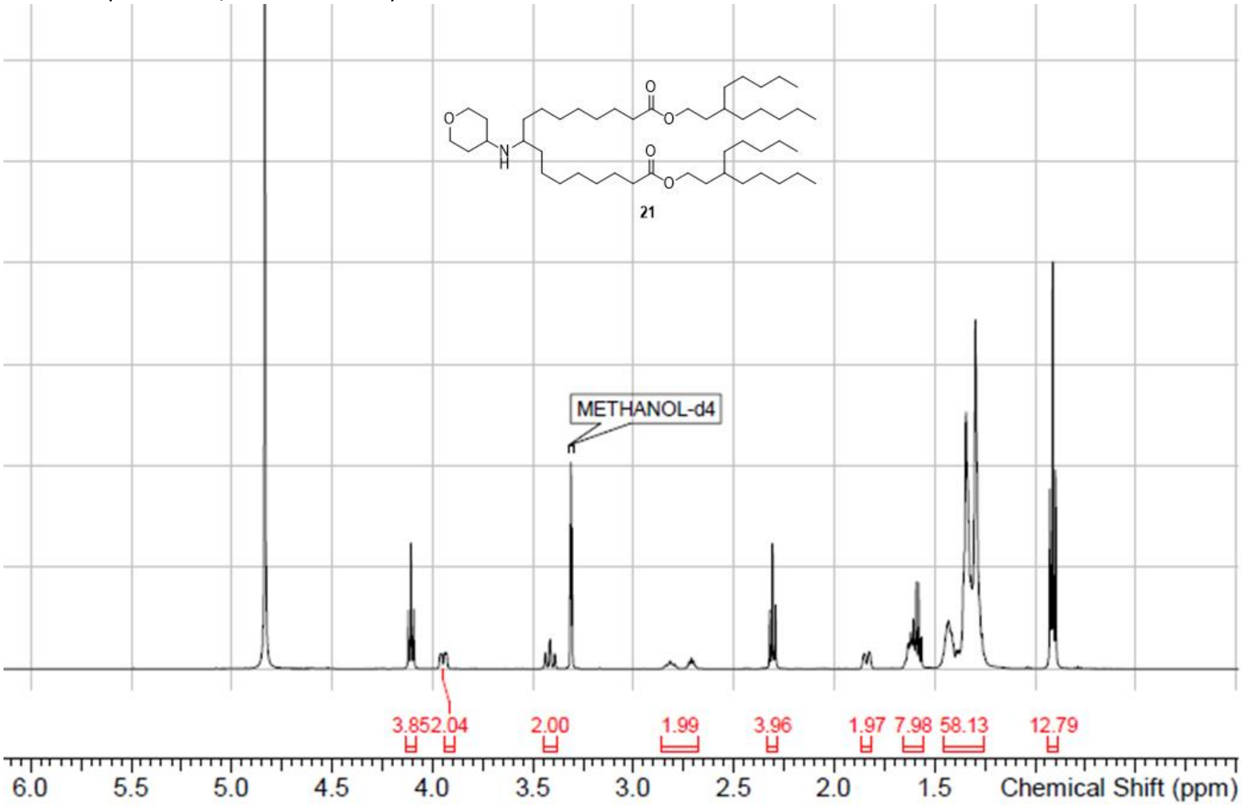

HSQC (500 MHz, Methanol-*d*4) of **21**

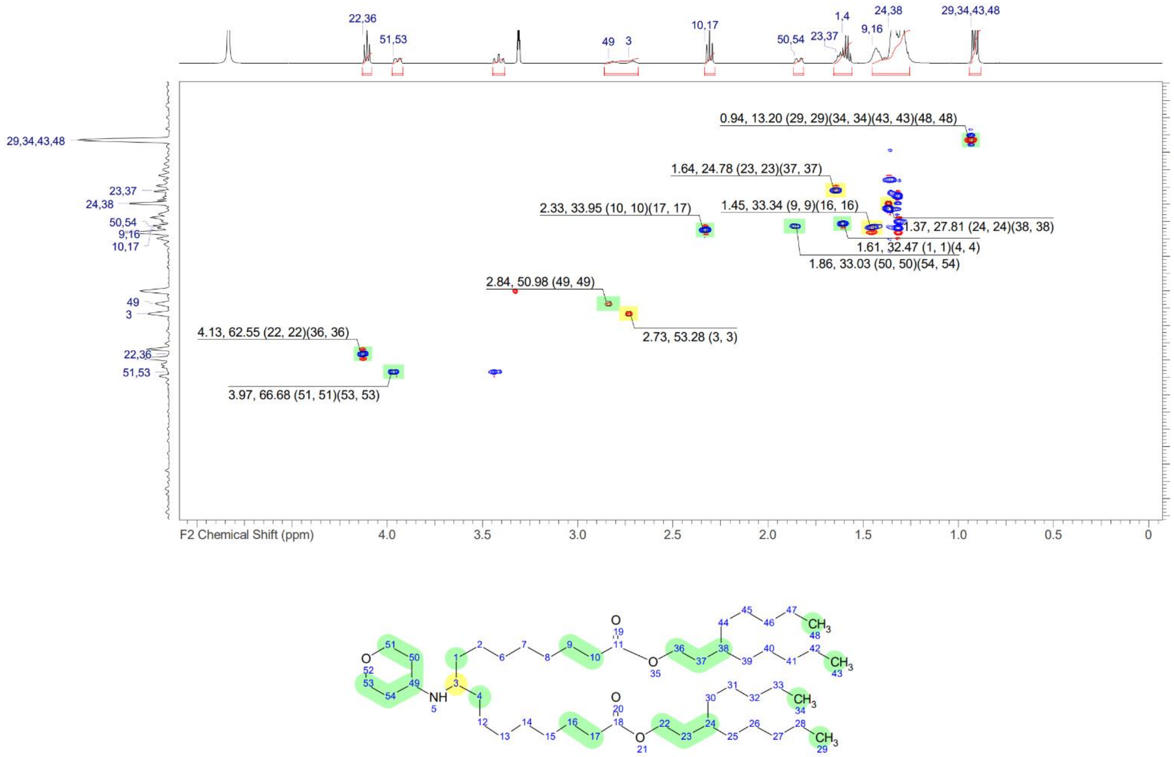

^{1}H NMR (500 MHz, Methanol-*d*4) of **22**

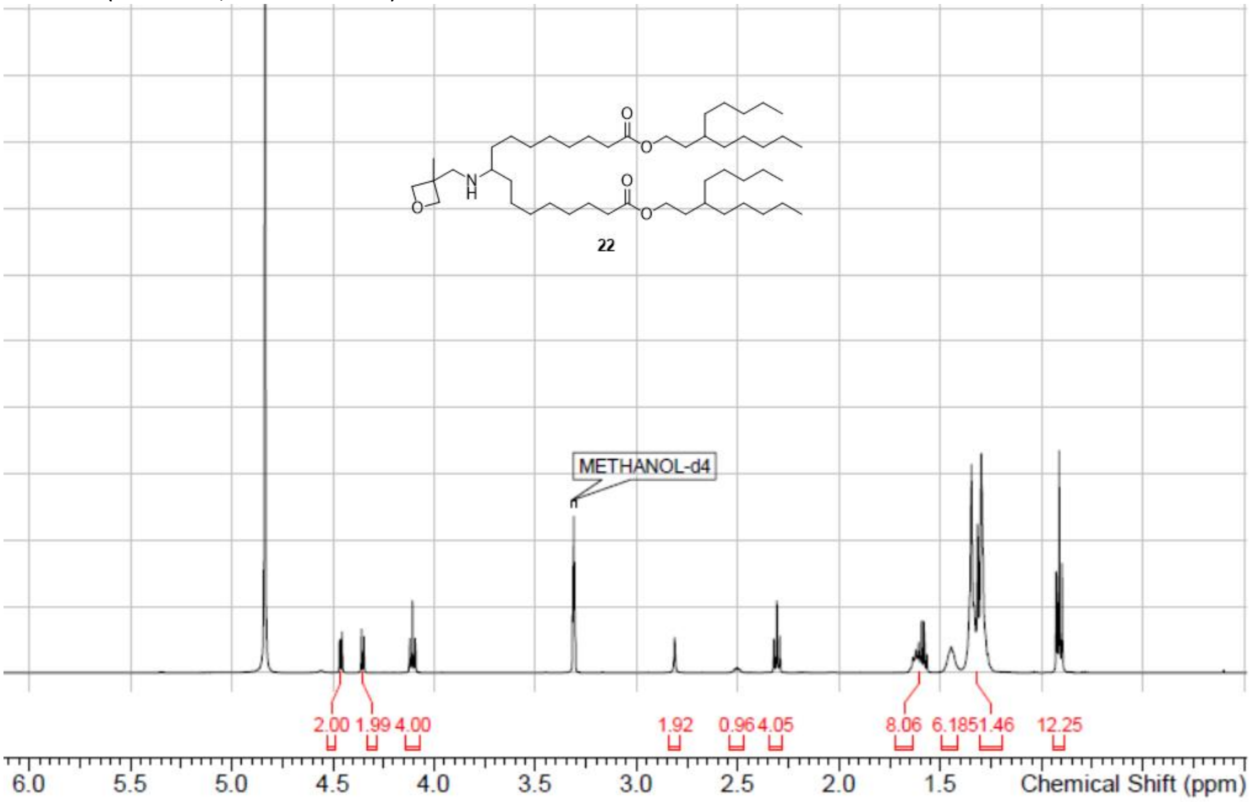

HSQC (500 MHz, Methanol-*d*4) of **22**

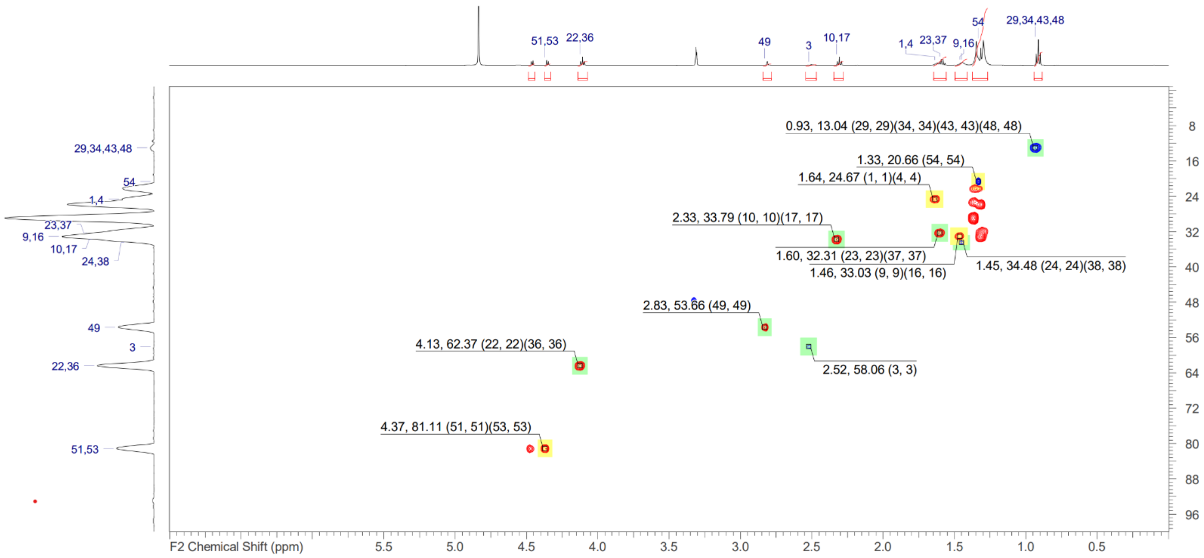

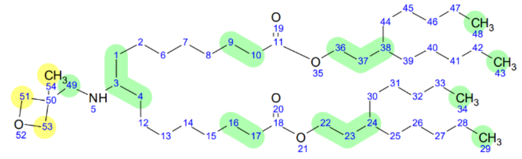

^{1}H NMR (500 MHz, Methanol-*d*4) of **23**

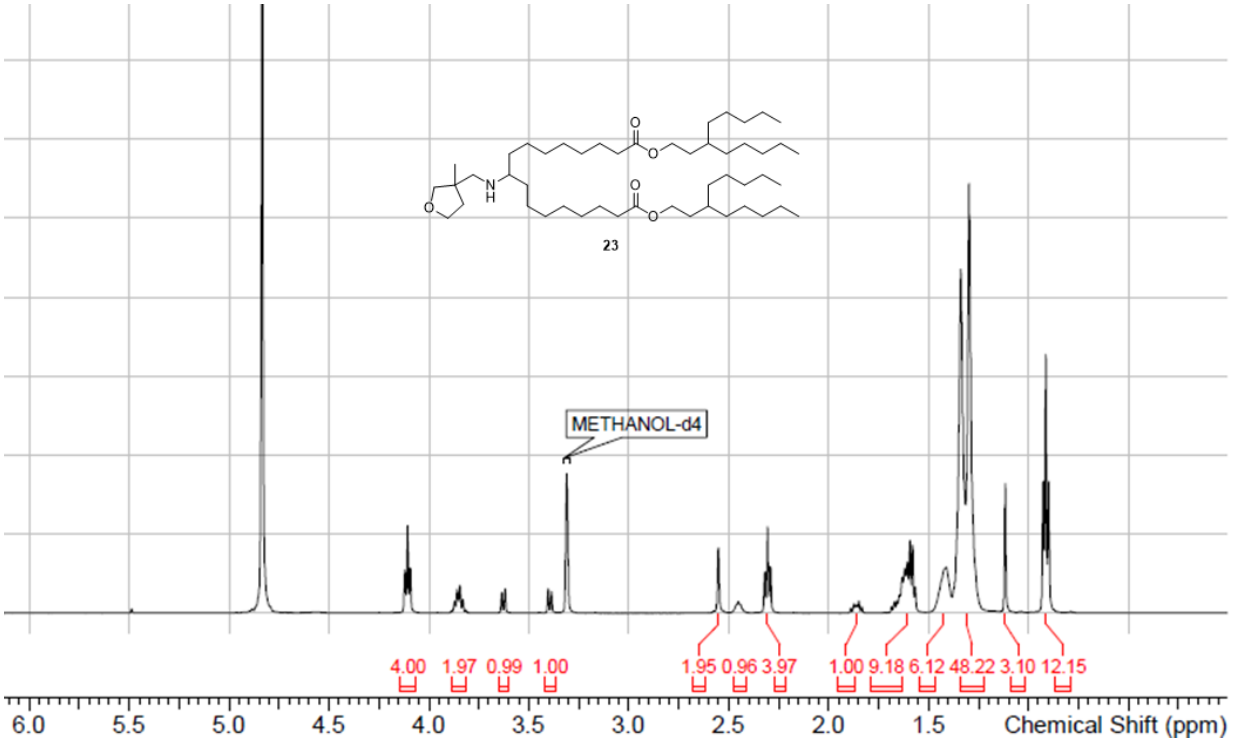

HSQC (500 MHz, Methanol-*d*4) of **23**

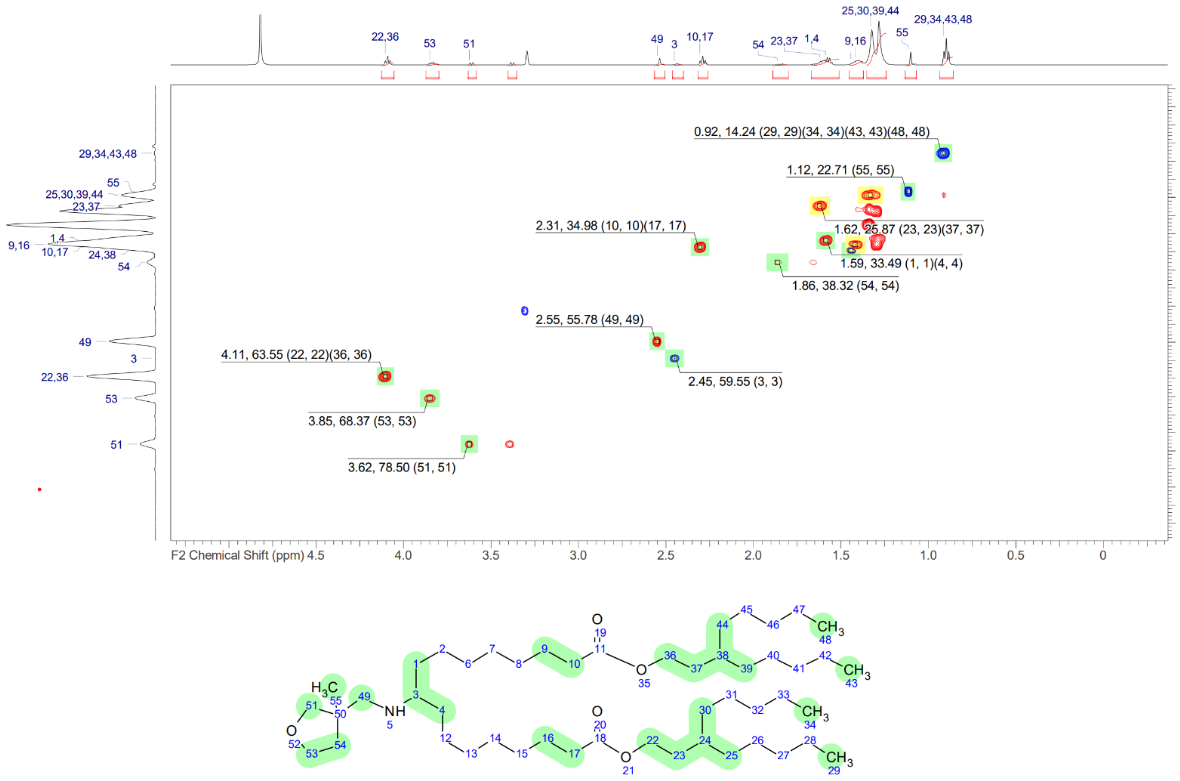

^{1}H NMR (500 MHz, Methanol-*d*4) of **24**

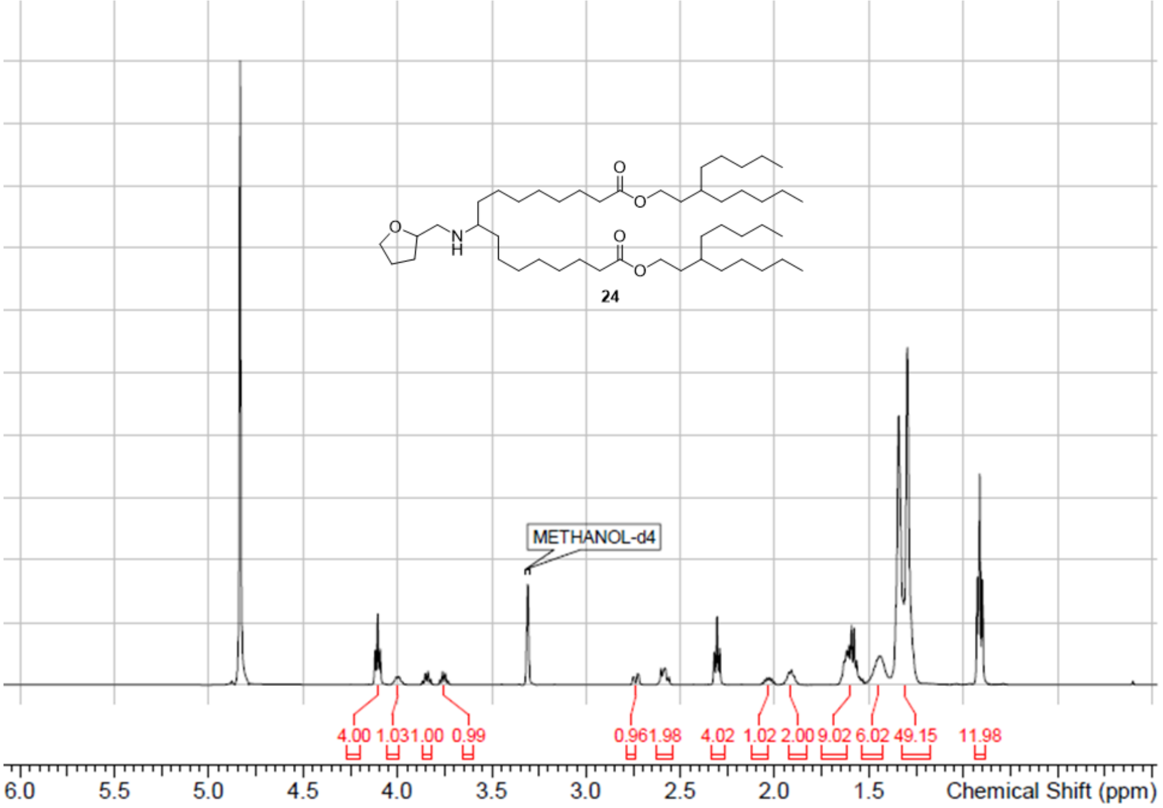

HSQC (500 MHz, Methanol-*d*4) of **24**

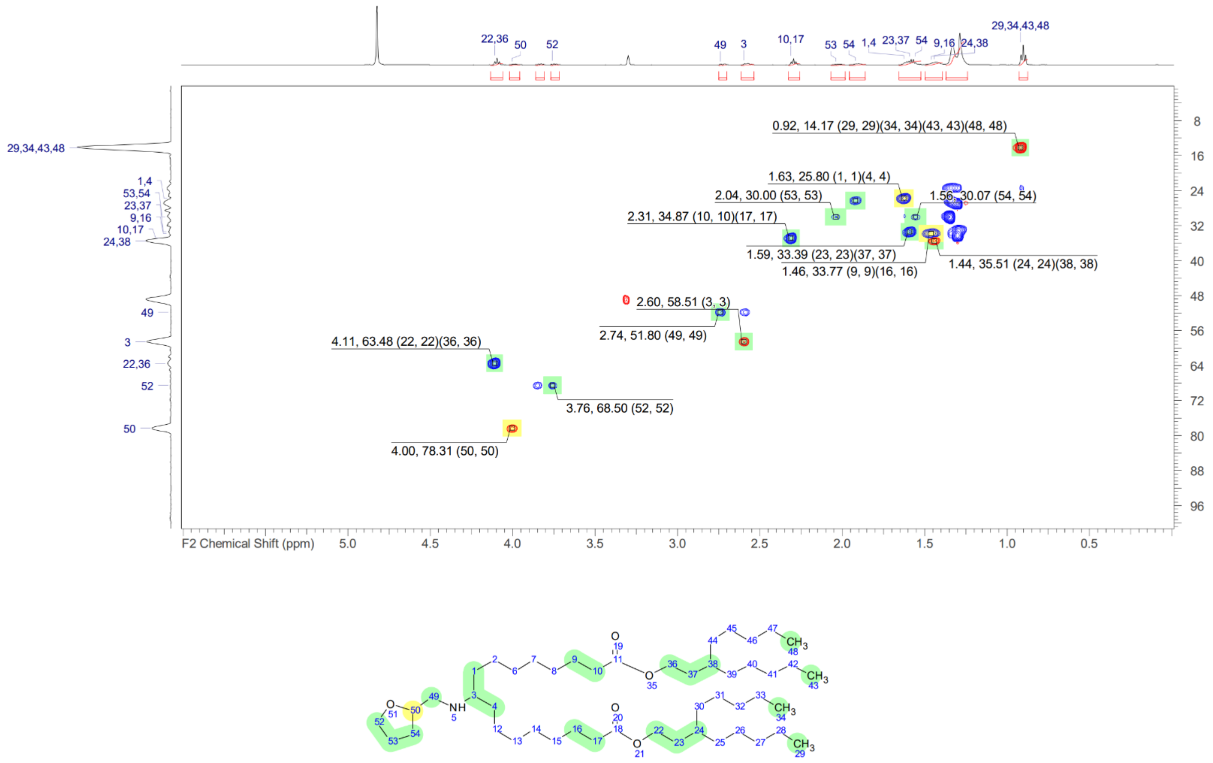

^{1}H NMR (400 MHz, Methanol-*d*4) of **25**

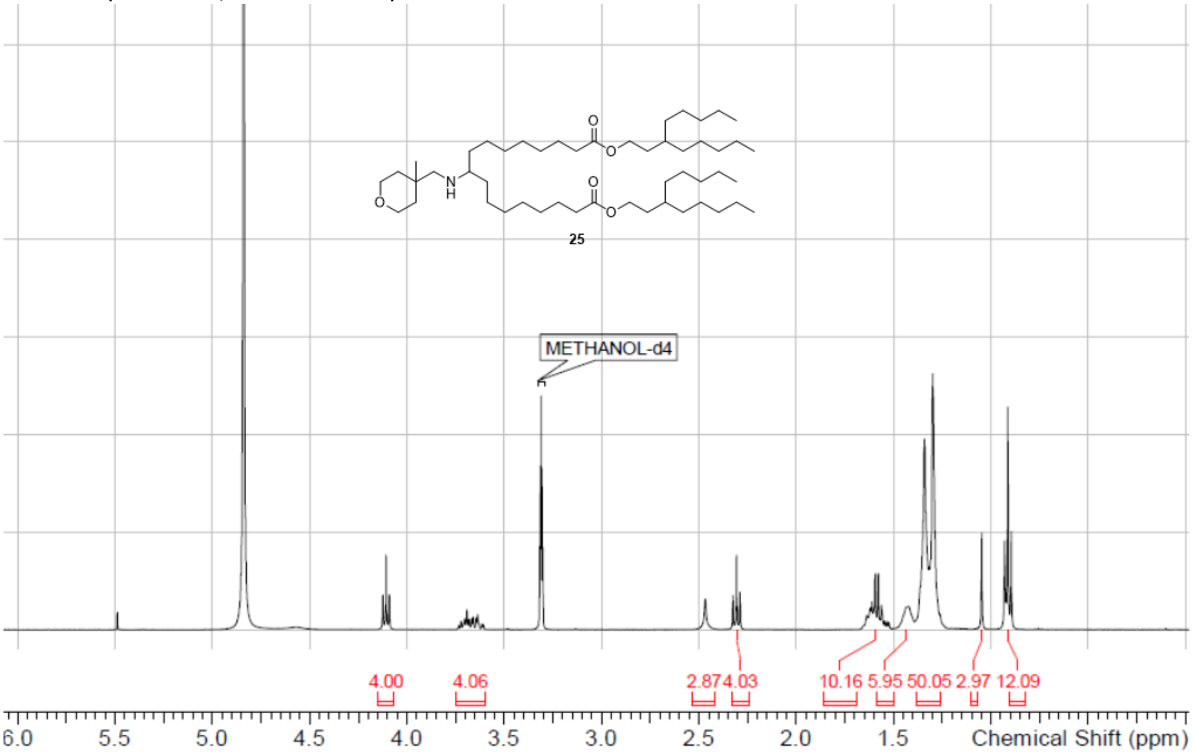

HSQC (400 MHz, Methanol-*d*4) of **25**

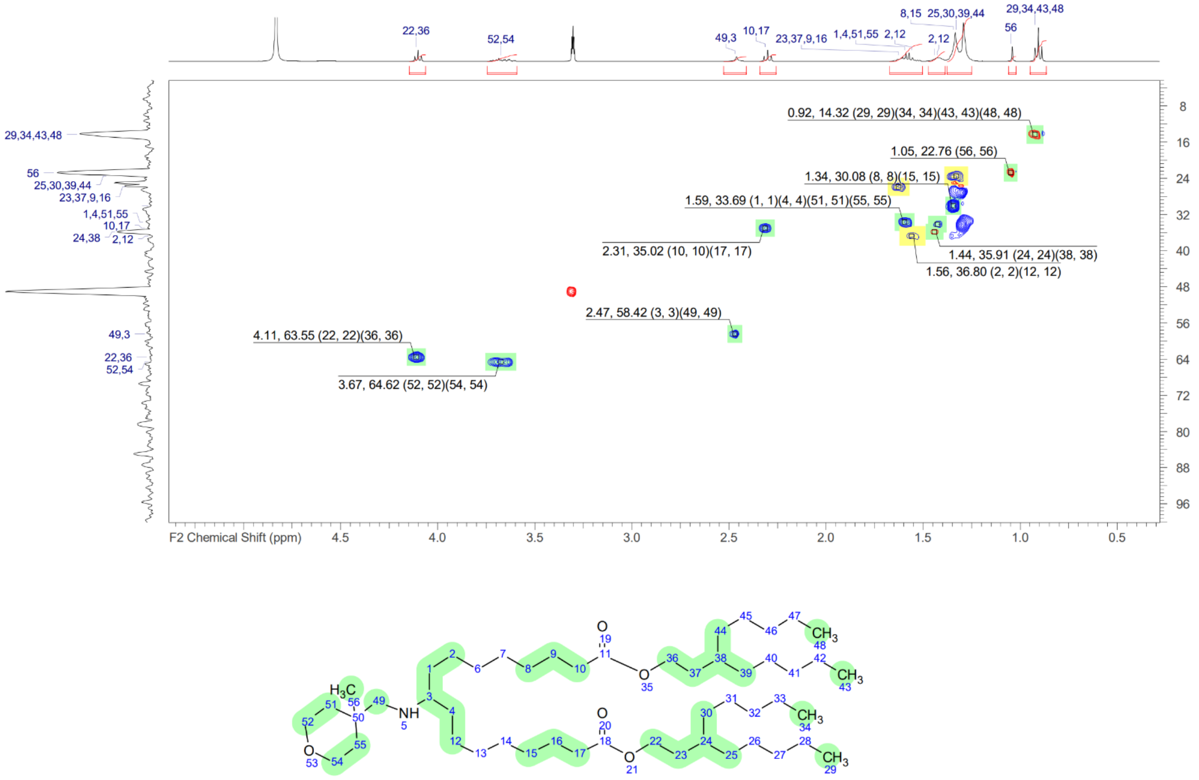

^{1}H NMR (500 MHz, Methanol-*d*4) of **26**

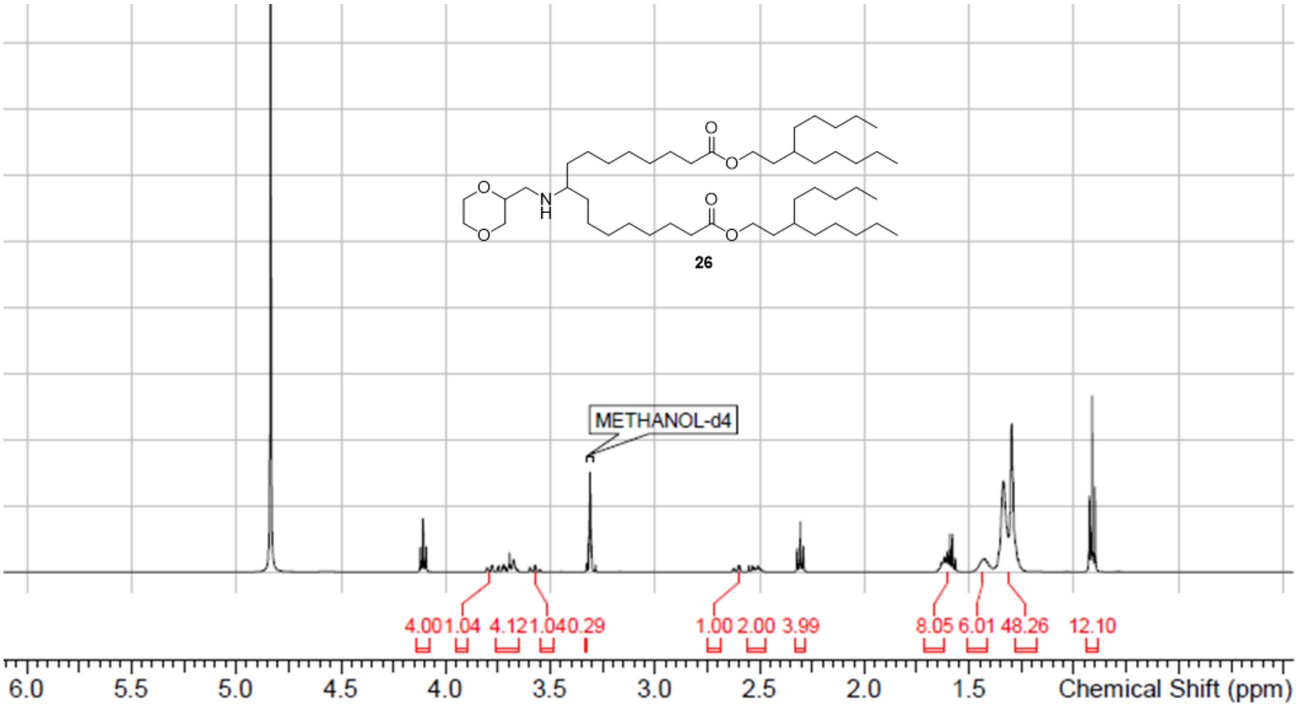

HSQC (500 MHz, Methanol-*d*4) of **26**

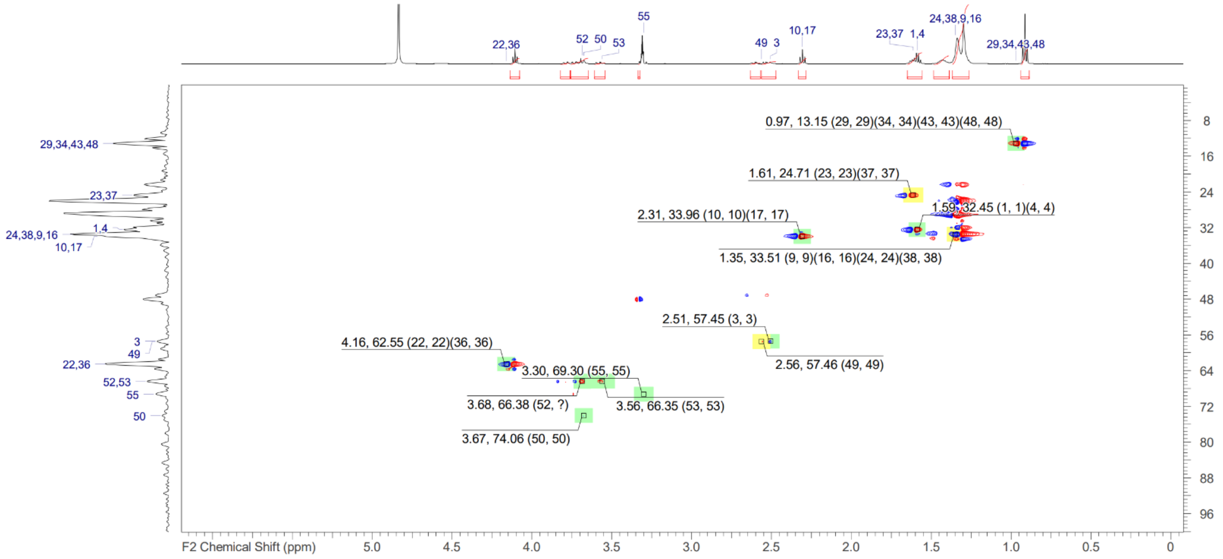

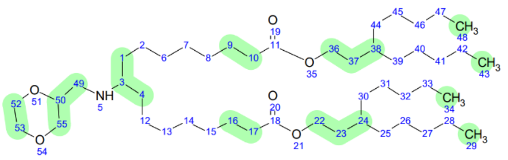

^{1}H NMR (500 MHz, Methanol-*d*4) of **27**

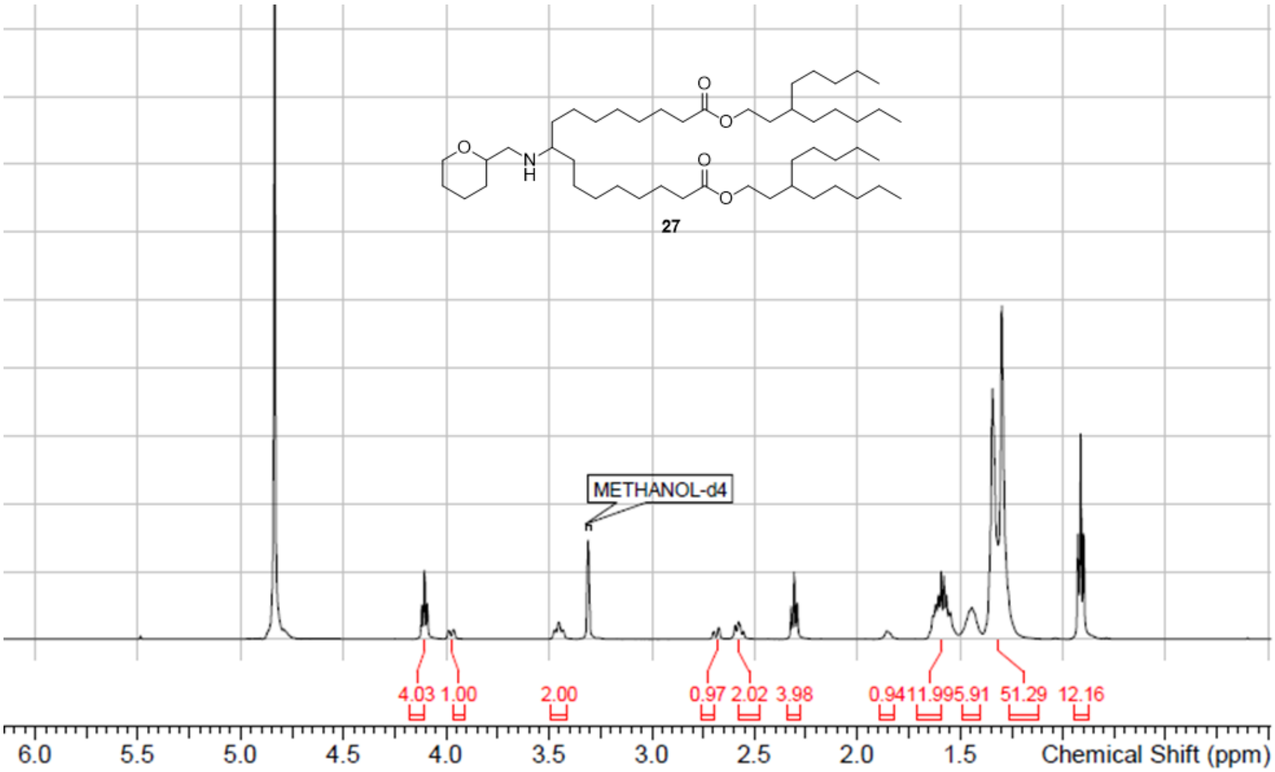

HSQC (500 MHz, Methanol-*d*4) of **27**

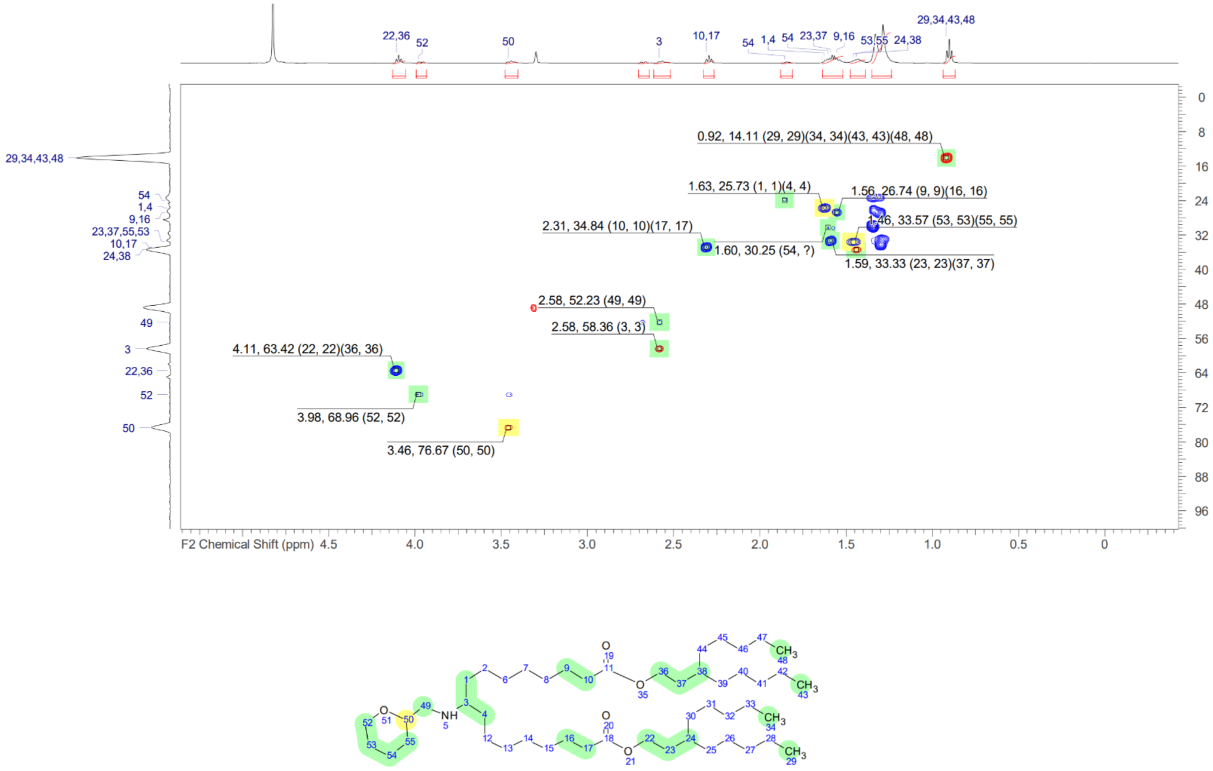

^{1}H NMR (400 MHz, Methanol-*d*4) of **28**

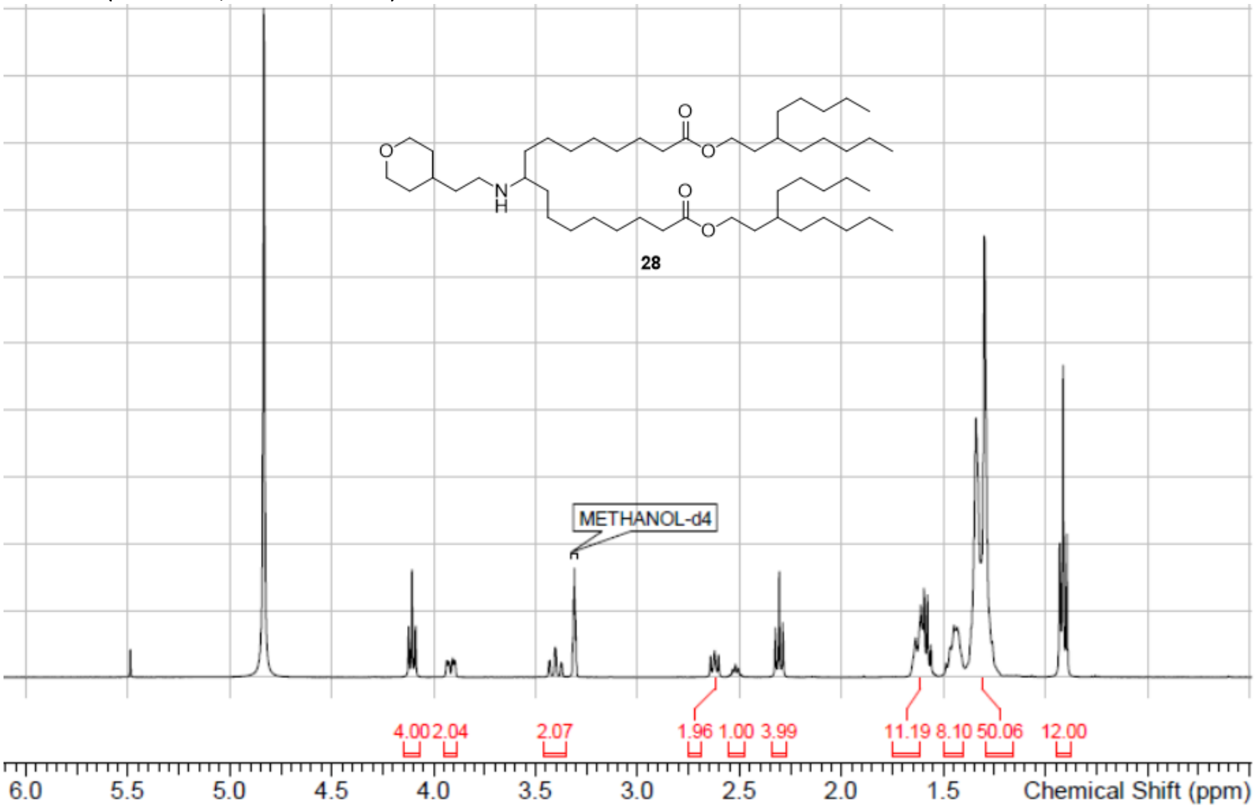

HSQC (400 MHz, Methanol-*d*4) of **28**

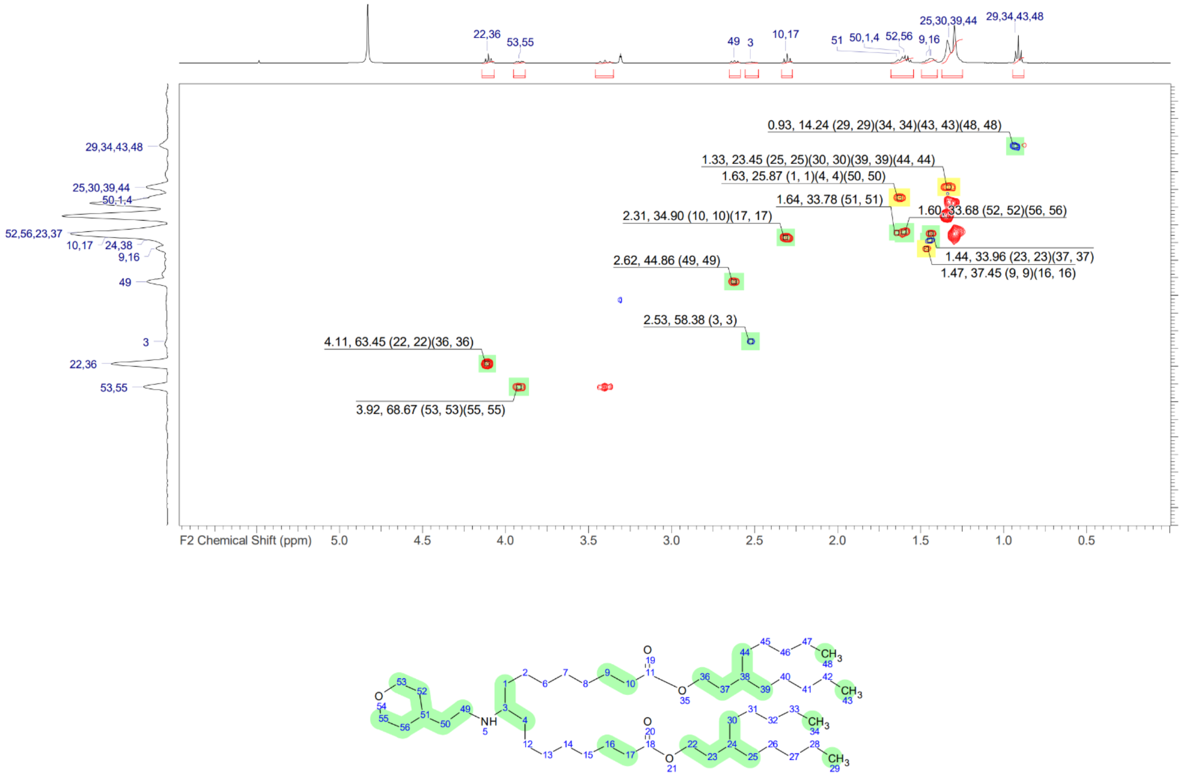

Supplement: MD-016-D5MD00115C-s001 [file MD-016-D5MD00115C-s001.pdf]
